# Stage-specific evolutionary dynamics in pediatric leukemia inferred using Bayesian stochastic modeling

**DOI:** 10.1371/journal.pone.0357755

**Published:** 2026-09-11

**Authors:** Seung-Hwan Kim

**Affiliations:** 1 Department of Biology, Fisher College, Boston, Massachusetts, United States of America; 2 Department of Pediatric Oncology, Dana-Farber Cancer Institute, Boston, Massachusetts, United States of America; Hirosaki University Graduate School of Medicine, JAPAN

## Abstract

Tumor evolution in pediatric leukemia is shaped by stochastic fluctuations and constraints imposed by developmental programs and therapeutic pressure. However, quantitative characterization of these dynamics remains challenging because of sparse longitudinal sampling and measurement noise in clinical data. In this study, we applied Bayesian calibration of stochastic Ornstein–Uhlenbeck (OU) models to characterize patient-specific tumor evolution trajectories in pediatric leukemia. Using a longitudinal cohort of KMT2A-rearranged leukemias (52 patients, 311 longitudinal observations corresponding to 294 unique patient-time points), we estimated parameters governing mean reversion and stochastic variability and assessed the stability and uncertainty of these estimates through simulation-based validation under empirically derived sampling conditions. A representative single-patient trajectory further illustrated patient-level evolutionary dynamics. Across the cohort, inferred OU parameters showed stage-associated tendencies, including higher average diffusion in refractory and very-early disease groups, although posterior intervals overlapped substantially and did not support sharply separated dynamical regimes. Simulation analyses indicated that Bayesian calibration provided stable posterior computation and interpretable uncertainty quantification under sparse sampling, although recovery remained parameter- and schedule-dependent and predictive performance was limited. These findings show that Bayesian-calibrated stochastic models provide a useful framework for interpreting longitudinal tumor evolution in pediatric leukemia. While not intended for clinical prediction, this approach offers a quantitative perspective on stage-dependent disease dynamics and highlights the importance of uncertainty-aware modeling in sparse clinical datasets.

## Introduction

Pediatric leukemia remains a leading cause of cancer-related mortality in children despite substantial advances in molecular characterization and therapy. Relapse continues to occur in a significant fraction of patients and is often driven by dynamic processes of clonal evolution, phenotypic plasticity, and therapeutic selection [1–5]. Understanding how these processes unfold over time within individual patients is a central challenge in modern oncology.

Longitudinal measurements of leukemia burden, including genomic and phenotypic readouts, provide a window into tumor evolution. However, such data are typically sparse, irregularly sampled, and noisy, limiting the ability of conventional statistical approaches to capture underlying biological dynamics. Most existing analyses rely on descriptive summaries or static comparisons and therefore do not explicitly model the stochastic and time-dependent nature of tumor evolution [1,6–10].

Stochastic processes offer a natural framework for modeling these dynamics. In particular, the Ornstein–Uhlenbeck process provides a principled representation of systems that fluctuate while tending toward a stabilizing state [11–14]. In the context of leukemia, this can be interpreted as a balance between forces that constrain phenotypic states, such as developmental programs or therapy, and stochastic fluctuations arising from intrinsic variability and clonal diversification [2,4,5,15].

However, applying such models to clinical data presents practical challenges. Sparse sampling limits parameter identifiability, and measurement noise complicates inference. In addition, uncertainty quantification is critical when interpreting patient-level trajectories but is often not addressed explicitly in standard estimation approaches [16–20].

Here, we apply Bayesian calibration of OU-based stochastic models to longitudinal pediatric leukemia data to address these challenges directly. Using a KMT2A-rearranged leukemia cohort and a representative individual trajectory, we aimed to characterize patient-specific evolutionary dynamics, quantify uncertainty in inferred parameters, and evaluate the robustness of inference under sparse sampling through simulation-based validation. We further examine whether inferred dynamical parameters exhibited consistent patterns across clinical stages [1,11,16,18,21].

**Fig 1:**
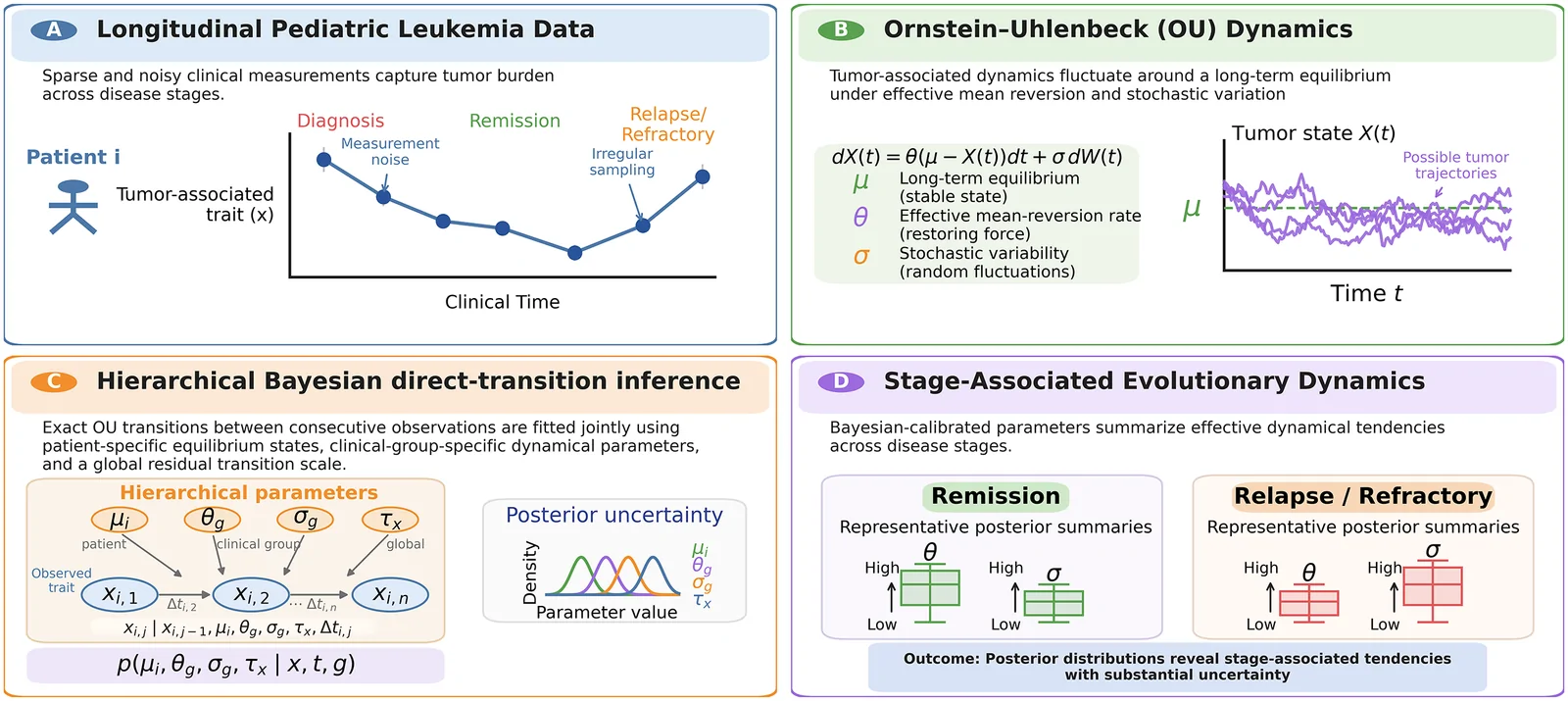
Overview of the hierarchical Bayesian stochastic modeling framework for stage-associated evolutionary dynamics in pediatric leukemia. (A) Sparse and irregular longitudinal measurements were converted into an annotation-derived tumor-associated trait 𝓍, observed across clinical disease stages. (B) Trait dynamics were represented using an Ornstein–Uhlenbeck process characterized by an equilibrium state μ, effective mean-reversion rate θ, and diffusion scale σ. (C) Exact OU transitions between consecutive observations were fitted jointly using a hierarchical Bayesian direct-transition model with patient-specific equilibrium states $\mu_{\text{𝒾}}$, clinical-group-specific mean-reversion and diffusion parameters $\theta_{g}$ and $\sigma_{g}$, and a global residual transition scale $\tau_{\text{𝓍}}$. (D) Posterior distributions were summarized across clinical stages to characterize stage-associated tendencies in effective mean reversion and stochastic variability while retaining substantial posterior uncertainty.

By treating stochastic modeling as a tool for biological interpretation rather than as a primary methodological contribution, this study provides a quantitative perspective on stage-specific tumor evolution in pediatric leukemia [21,22]. An overview of the study design, modeling framework, and biological interpretation is shown in Fig 1.

## Methods

### Study overview

This study developed a hierarchical Bayesian stochastic modeling framework to characterize longitudinal tumor-associated dynamics in pediatric leukemia under sparse and irregular clinical sampling (Fig 1). The analytical workflow consisted of five components: (i) preprocessing and normalization of longitudinal tumor measurements and construction of direct patient-level transitions; (ii) formulation of an Ornstein–Uhlenbeck (OU) stochastic process using its exact transition distribution for irregular observation intervals; (iii) joint hierarchical Bayesian estimation of patient-specific equilibrium states and clinical-group-specific dynamical parameters using the No-U-Turn Sampler; (iv) simulation-based evaluation of OU parameter recovery and estimator robustness under clinically realistic sampling schedules; and (v) posterior predictive evaluation and comparison of effective dynamical properties across clinical disease stages.

The primary objective was not to develop or validate a clinical prediction model. Rather, the framework was designed to provide uncertainty-aware statistical summaries of patient-level longitudinal dynamics and to evaluate whether effective mean-reversion and stochastic-variability parameters differed across predefined clinical disease stages. The principal mathematical notation used throughout the model formulation is summarized in Table 1.

**Table 1:** Summary of notation used throughout the hierarchical OU model.

Symbol	Meaning	Interpretation
X(t)	Latent longitudinal tumor-associated trait	Modeled stochastic state
x	Observed annotation-derived trait	Longitudinal measurement
t	Time	Years from diagnosis
Δt	Observation interval	Time between measurements
$\mu_{\text{𝒾}}$	Patient-specific equilibrium	Long-term effective state
$\theta_{g}$	Group mean-reversion rate	Strength of return toward equilibrium
$\sigma_{g}$	Diffusion scale	Stochastic variability
$\tau_{x}$	Residual transition SD	Residual uncertainty
W(t)	Wiener process	Brownian noise

### Data source and longitudinal trait construction

Longitudinal measurements were obtained from the publicly available pediatric KMT2A-rearranged leukemia cohort reported by Ahlgren et al. [1]. The complete dataset comprised 52 patients and 311 longitudinal observations spanning diagnosis, treatment, remission, and relapse- or refractory-associated clinical stages.

For each sample, observation time was converted from days to years as


$$\begin{array}{c} t=\frac{\text{Day}}{\text{3}\text{6}\text{5}.\text{2}\text{5}}\text{.} \end{array}$$

Two rule-based longitudinal variables were then derived from the available clinical and molecular annotations: a continuous tumor-state trait, denoted 𝓍, and an annotation-derived ordinal burden surrogate, denoted 𝓃.

The 𝓍 value was assigned hierarchically according to the following rules. When the *ClinicalData* field contained an MRD percentage, the percentage was extracted and converted to a proportion between 0 and 1. In the absence of an explicit MRD value, diagnosis and relapse samples were assigned 𝓍=1.00, complete-remission samples were assigned 𝓍=0.05, samples with detected KMT2A fusion were assigned 𝓍=0.50, and other longitudinal samples were assigned 𝓍=0.10. Records lacking sufficient information for assignment were left undefined.

The 𝓃 variable was constructed as an ordinal surrogate of detectable leukemic or clonal burden. Samples with detected KMT2A fusion were assigned 𝓃=2. Samples with an explicit MRD value or leukemic cells detected at complete remission were assigned 𝓃=1. Samples without these indicators were assigned 𝓃=0. Thus, 𝓃 represented three ordered annotation-derived states rather than a direct molecular count of independently reconstructed clones.

For each patient-time observation, the inferred 𝓍 and 𝓃 values were written in tidy long format with columns *Patient_ID, series*, t, and *value*, where series identified either the 𝓍 or 𝓃 trajectory. The resulting table was manually reviewed and stored in the “Series” worksheet of *kmt2a_longitudinal_clean.xlsx*, which served as the canonical direct-transition input.

A deterministic preprocessing and quality-control workflow was subsequently applied to this worksheet. Patient identifiers and series labels were stripped of extraneous whitespace, series labels were converted to lowercase, and only records labeled 𝓍 or 𝓃 were retained. Observation times and values were converted to numeric format, incomplete rows were excluded, and records were sorted reproducibly by patient, series, and time before export to *series_auto.csv*. No missing intermediate observations were imputed, and no additional within-patient standardization or rescaling was applied during this export step.

The 𝓍 series served as the observed longitudinal input to the OU model. The 𝓃 series was used for descriptive visualization of annotation-derived burden dynamics and for examining its association with the modeled 𝓍 trait. Both variables were interpreted as reproducible, rule-based summaries of the available clinical annotations rather than direct measurements of a single biological mechanism.

### Clinical-stage assignment

Patients were assigned to the predefined clinical groups reported in the original KMT2A-rearranged leukemia cohort of Ahlgren et al. [1]. The present study did not redefine or independently reconstruct these clinical categories. The six modeled groups were Early, Early/refractory, Late, Remission, Very early, and Very early/refractory. Each modeled patient retained the single clinical-group label provided in the source dataset for hierarchical Bayesian inference. Patient-to-group assignments are provided in S4 Data, and group-specific sample sizes are summarized in S4 Table.

### Sampling characteristics

Longitudinal sampling characteristics were summarized before OU model fitting using all 52 patients in the descriptive cohort. For each patient, we calculated the total number of longitudinal observations, the number of unique patient-time sampling points, follow-up duration, and the intervals between consecutive unique sampling times. Follow-up duration was defined as the difference between the latest and earliest observation times. After unique sampling times were ordered chronologically, consecutive intervals were calculated as


$$\begin{array}{c} \Delta t_{\text{𝒾},\text{𝒿}}=t_{\text{𝒾},\text{𝒿}}-t_{\text{𝒾},\text{𝒿}-\text{1}}\text{,} \end{array}$$

and only strictly positive intervals were retained.

Patient-level sampling irregularity was quantified as the coefficient of variation of positive consecutive intervals,


$$\begin{array}{c} {\text{CV}}_{\Delta t,\text{\{𝒾}}\}=\frac{\text{SD}\left(\Delta t_{\text{\{𝒾}}\}\right)}{\text{mean}\left(\Delta t_{\text{\{𝒾}}\}\right)}\text{.} \end{array}$$

This measure was calculated only for patients with at least three unique sampling timepoints, corresponding to at least two positive consecutive intervals. Patients with fewer than three unique timepoints were retained in the overall sampling summaries but were not assigned an irregularity index.

Across the cohort, 311 longitudinal observations corresponded to 294 unique patient-time sampling points. The median number of unique timepoints per patient was 5 (range, 1–18), the median follow-up duration was 1.077 years, and the median positive consecutive sampling interval was 0.088 years. Among the 30 patients with sufficient sampling intervals, the median patient-level irregularity index was 0.956. Complete cohort-level and patient-level sampling summaries are provided in S1 Table and S1 Fig.

### OU process and exact transition density

Longitudinal dynamics of the annotation-derived tumor-associated trait were modeled using an OU stochastic differential equation [11]– [14], [23],


$$\begin{array}{c} dX_{t}=\theta \left[\mu -X(t)\right]dt+\sigma dW(t), \end{array}$$

where X(t) denotes the modeled longitudinal state, μ is the equilibrium or mean-reverting level, θ>0 is the effective mean-reversion rate, σ>0 is the diffusion scale, and W(t) is a standard Wiener process.

The drift term,


$$\begin{array}{c} \theta \left[\mu -X(t)\right], \end{array}$$

describes the tendency of the process to return toward μ, whereas the diffusion term,


$$\begin{array}{c} \sigma dW(t), \end{array}$$

represents stochastic variation around the deterministic mean-reverting trajectory. Under this model, larger θ indicates more rapid effective return toward equilibrium, larger σ indicates greater stochastic transition variability, and μ represents the long-term equilibrium level of the modeled trait.

These parameters were interpreted as effective statistical summaries under the assumed OU process. They were not interpreted as direct measurements of biological selection, regulatory mechanisms, or causal evolutionary forces.

Because clinical observations occurred at sparse and unequal intervals, inference used the exact OU conditional transition density rather than numerical discretization of the stochastic differential equation or interpolation of unobserved intermediate states.

**Hierarchical direct-transition model**

For patient 𝒾, longitudinal trait dynamics were modeled as


$$\begin{array}{c} dX_{\text{\{𝒾}}\}(t)=\theta_{g\left(\text{\{𝒾}\}\right)}\left[\mu_{\text{\{𝒾}}\}-X_{\text{\{𝒾}}\}(t)\right]dt+\sigma_{g\left(\text{\{𝒾}\}\right)}dW_{\text{\{𝒾}}\}(t), \end{array}$$

where $X_{\text{𝒾}}(t)$ denotes the modeled tumor-associated trait for patient 𝒾, $\mu_{\text{𝒾}}$ is the patient-specific equilibrium state, and g(𝒾) identifies the patient’s predefined clinical group. The parameters $\theta_{g\left(\text{𝒾}\right)}$ and $\sigma_{g\left(\text{𝒾}\right)}$ denote the mean-reversion rate and diffusion scale shared by patients within clinical group g(𝒾), respectively. A global residual transition-scale parameter $\tau_{x}$ was estimated across the modeled cohort to capture variation not explained by the OU process, including residual measurement, annotation, and model uncertainty.

For consecutive observations separated by an interval $\Delta t_{i,j}$, the likelihood was


$$\begin{array}{c} \;X_{\text{\{𝒿}}\;\}|\;X_{\text{\{𝒿}}-\text{1}\},\mu_{\text{\{𝒾}}\},\theta_{g(\text{\{𝒾}})\},\sigma_{g\left(\text{\{𝒾}\}\right)},\tau_{x}\;\sim \;N\left(m_{\text{\{𝒾}},\text{\{𝒿}\}\},\sqrt{\upsilon_{\text{\{𝒾}},\text{\{𝒿}}\}+\tau_{x}^{\text{2}}\}\right), \end{array}$$

where $m_{\text{𝒾},\text{𝒿}}$ is the exact OU conditional mean and


$$\begin{array}{c} \upsilon_{\text{\{𝒾}},\text{\{𝒿}\}\}=\frac{{\sigma_{g(\text{\{𝒾}})}^{\text{2}}}{\}}\text{2}\theta_{g(\text{\{𝒾}})\}\left[\text{1}-\text{exp}\left(-\text{2}\theta_{g\left(\text{\{𝒾}\}\right)}\Delta t_{\text{\{𝒾}},\text{\{𝒿}\}\}\right)\right], \end{array}$$

is the exact OU process variance. Thus, the total transition standard deviation was


$$\begin{array}{c} \sqrt{\upsilon_{\text{\{𝒾}},\text{\{𝒿}}\}+\tau_{x}^{\text{2}}\}. \end{array}$$

This formulation directly accounts for unequal observation intervals and avoids numerical time discretization between clinical measurements. The model was fitted jointly to the 47 patients with at least one valid positive-time longitudinal transition. Patient-specific equilibrium states $\mu_{\text{𝒾}}$ were modeled hierarchically, whereas $\theta_{g}$ and $\sigma_{g}$ were estimated at the clinical-group level and shared by patients assigned to the same group.

The posterior estimate of $\mu_{\text{𝒾}}$ represents the patient-specific equilibrium level of the modeled trait. The parameter $\theta_{g}$ summarizes the effective rate at which deviations from equilibrium tend to decay within clinical group g, whereas $\sigma_{g}$ summarizes stochastic transition variability. These quantities characterize the behavior of the fitted statistical process and should be interpreted as effective statistical summaries under the assumed OU model rather than as direct measurements of biological mechanisms or causal selective forces.

### Prior distributions

Weakly informative priors were used to regularize inference under sparse and irregular longitudinal sampling. Clinical-group-specific mean-reversion and diffusion parameters were defined through unconstrained latent variables,

$\theta_{g}^{\text{raw}}\sim N(\text{0},\text{1})$, $\sigma_{g}^{\text{raw}}\sim N(\text{0},\text{1})$,

and transformed to positive values as


$$\begin{array}{c} \theta_{g}=\text{0}.\text{0}\text{5}+softplus\left(\theta_{g}^{\text{raw}}\right), \end{array}$$

and


$$\begin{array}{c} \sigma_{g}=\text{0}.\text{0}\text{2}+\text{0}.\text{5}\text{0}\;softplus\left(\sigma_{g}^{\text{raw}}\right), \end{array}$$

where $softplus\left(\text{𝓏}\right)=log\left(\text{1}+e^{\text{𝓏}}\right)$. The additive constants imposed lower bounds of 0.05 for $\theta_{g}$ and 0.02 for $\sigma_{g}$, preventing numerically unstable values arbitrarily close to zero.

The global residual transition scale was assigned the prior


$$\begin{array}{c} \tau_{x}\;\sim \;HalfNormal(\text{0}.\text{3}\text{0})\text{.} \end{array}$$

Patient-specific equilibrium states were modeled hierarchically using the non-centered parameterization

$\mu_{\mu }\;\sim \;N(\text{0},\text{1})$, $\mu_{\sigma }\;\sim \;HalfNormal(\text{1})$, ${\text{𝓏}}_{\text{𝒾}}\;\sim \;N(\text{0},\text{1})$,

with


$$\begin{array}{c} \mu_{\text{\{i}}\}=\mu_{\mu }+\mu_{\sigma }{\text{𝓏}}_{\text{𝒾}}. \end{array}$$

This formulation allowed patient-specific equilibrium estimates to be partially pooled through the cohort-level location and scale parameters while improving sampling efficiency under sparse patient-level observations. A constant $\varepsilon ={\text{1}\text{0}}^{-\text{8}}$ was added to the total transition variance solely for numerical stability.

### Posterior computation and convergence

Posterior inference was performed in PyMC 5.24.1 using the No-U-Turn Sampler (NUTS), an adaptive Hamiltonian Monte Carlo algorithm [16,17,19,24,25]. Four independent Markov chains were initialized using the *jitter+adapt_diag* strategy. Each chain used 2,500 tuning iterations followed by 2,000 retained posterior draws, yielding 8,000 retained draws across chains. The target acceptance probability was set to 0.97, and the random seed was fixed at 13. The default maximum tree depth was used.

Posterior computation was performed in a dedicated Conda environment (plos-ou) using Python 3.12.13, ArviZ 0.22.0, NumPy 2.5.1, SciPy 1.18.0, pandas 3.0.3, and Matplotlib 3.11.0. The analyses were run on an Apple silicon system using macOS/Darwin 25.5.0 on the arm64 architecture. The complete environment specification and package inventory were archived as *environment.yml* and *conda_package_versions.txt*, respectively. The uploaded package inventory confirms the principal software versions and the Conda environment configuration.

Convergence was evaluated using multiple complementary diagnostics, including the potential scale reduction statistic ($\hat{R}$), bulk and tail effective sample sizes (ESS), visual inspection of Markov-chain trace plots, the number of divergent transitions, and maximum tree-depth events. Posterior summaries were considered acceptable only after chains demonstrated stable mixing, $\hat{R}$ values close to 1.00, adequate effective sample sizes, and no material sampling pathologies. Complete convergence statistics are provided in *ou_direct_transition_convergence_summary.csv*, and the full sampling configuration and run metadata are reported in *ou_direct_transition_run_metadata.csv*. All posterior summaries were interpreted in conjunction with the reported convergence diagnostics, and no results were retained without first evaluating chain mixing, $\hat{R}$, effective sample sizes, divergent transitions, and tree-depth behavior.

### Simulation-based calibration benchmark

Because clinical longitudinal datasets are typically sparse and irregularly sampled, simulation studies were performed to evaluate OU parameter recovery and compare the robustness of alternative estimation methods under realistic sampling conditions.

Synthetic OU trajectories were generated over a range of equilibrium states (μ), mean-reversion rates (θ), and diffusion scales (σ) representative of the clinical cohort. Observation schedules were sampled from the empirical distribution of patient-specific sampling times to reproduce the irregular longitudinal structure of the KMT2A-rearranged leukemia dataset. For each simulated dataset, the same direct-transition OU formulation used in the clinical analysis was fitted using four estimation approaches: Bayesian inference with NUTS, maximum-likelihood estimation (MLE), the method of moments (MoM), and an imputed method-of-moments approach for sparse trajectories.

Parameter recovery was evaluated by comparing estimated and true parameter values for μ, θ, and σ. Performance metrics included parameter recovery bias, root mean squared error (RMSE), posterior or estimator uncertainty, interval coverage where applicable, and method-specific reliability. These analyses assessed statistical identifiability and estimator robustness under clinically realistic sampling conditions rather than validating biological mechanisms.

Method reliability was evaluated using prespecified criteria. Bayesian estimates were considered reliable only when posterior sampling completed successfully, finite parameter estimates were obtained, no boundary constraints were encountered, and predefined Markov chain Monte Carlo convergence diagnostics were satisfied. MLE estimates were considered reliable when optimization converged successfully, finite estimates were obtained, and no parameter reached a numerical boundary. Direct method-of-moments estimates were considered reliable when finite, identifiable parameter estimates were obtained without boundary violations. The imputed method-of-moments approach was considered reliable when finite parameter estimates were obtained after imputation without numerical boundary contact.

The simulation benchmark therefore quantified not only parameter recovery accuracy but also the practical reliability of each estimation approach under sparse and irregular longitudinal sampling.

### Patient-level and group-level summaries

A hierarchical direct-transition OU model was fitted jointly to all eligible patients. The equilibrium state, $\mu_{\text{𝒾}}$, was estimated for each patient using a hierarchical prior, whereas the mean-reversion and diffusion parameters, $\theta_{g}$ and $\sigma_{g}$, were estimated at the clinical-group level and shared among patients assigned to the same group. A global residual transition-scale parameter, $\tau_{x}$, was shared across the modeled cohort.

Patient-level posterior summaries included the patient-specific equilibrium state $\mu_{\text{𝒾}}$, the clinical-group-specific $\theta_{g}$ and $\sigma_{g}$ values applicable to that patient, posterior mean and median trajectories, 95% posterior predictive intervals, and patient-level posterior predictive coverage. The global residual transition scale $\tau_{x}$ was summarized at the cohort level rather than estimated separately for each patient.

To evaluate broader differences among predefined clinical stages, posterior samples of the group-specific parameters were summarized directly for each clinical group. Group-level summaries included posterior means and 95% highest-density intervals for $\theta_{g}$, $\sigma_{g}$, and derived OU quantities. Pairwise posterior probabilities were additionally calculated to quantify the probability that a parameter or derived quantity was larger in one clinical group than in another.

Two derived quantities were calculated from posterior samples. The quantity


$$\begin{array}{c} \frac{\sigma_{g}^{\text{2}}}{\theta_{g}}, \end{array}$$

shown in Fig 3C and used for pairwise group comparisons, was termed the drift-to-noise ratio. The conventional OU stationary variance,


$$\begin{array}{c} \frac{\sigma_{g}^{\text{2}}}{\text{2}\theta_{g}}, \end{array}$$

was additionally reported in S4 Table. Because the drift-to-noise ratio is exactly twice the stationary variance, both quantities preserve the same ordering among clinical groups but differ in scale.

### Posterior predictive evaluation

Model adequacy was evaluated using posterior predictive simulations generated from the fitted hierarchical direct-transition OU posterior. For each modeled patient, posterior draws of the patient-specific equilibrium state, $\mu_{\text{𝒾}}$, the corresponding clinical-group-specific parameters, $\theta_{g(i)}$ and $\sigma_{g(i)}$, and the global residual transition scale, $\tau_{x}$, were used to generate predictive longitudinal trajectories at the observed sampling times. These trajectories were compared with the observed annotation-derived 𝓍 values.

Patient-level model performance was summarized using four complementary diagnostics. Root mean squared error was calculated as


$$\begin{array}{c} {\text{RMSE}}_{\text{\{𝒾}}\}=\sqrt{\frac{\text{1}}{n_{\text{\{𝒾}}}}\sum_{\text{\{𝒿}}=\text{1}{\}}^{n_{\text{\{𝒾}}}\}{\left(x_{ij}-{\hat{x}}_{ij}\right)}^{\text{2}}\}, \end{array}$$

where $x_{i,j}$ is the observed trait value and ${\overbrace{x}}_{i,j}$ is the posterior-mean predicted value for patient 𝒾 at observation 𝒿.

Posterior predictive coverage was defined as the proportion of observed trait values falling within the corresponding patient-level 95% posterior predictive intervals.

Predictive $R^{\text{2}}$ was calculated as


$$\begin{array}{c} R_{\text{pred},\text{\{𝒾}}{\}}^{\text{2}}=\text{1}-{\frac{\sum_{\text{\{𝒿}}}{\left(x_{ij}-{\hat{x}}_{ij}\right)}}^{\text{2}}\}\sum_{\text{\{𝒿}}{\left(x_{ij}-{\overset{―}{x}}_{\text{\{𝒾}}\}\right)}^{\text{2}}\}, \end{array}$$

where ${\overset{―}{x}}_{\text{𝒾}}$ is the patient-specific mean observed trait value. Negative predictive $R^{\text{2}}$ values indicate that the posterior-mean trajectory performed worse than the patient-specific observed mean as a reference predictor.

Mean transition log-likelihood was calculated as the posterior expected Gaussian log-likelihood averaged across the valid observed direct transitions for each patient.

These diagnostics evaluate distinct aspects of model adequacy, including average prediction error, uncertainty calibration, explanatory performance relative to a patient-specific mean baseline, and transition-level probabilistic fit. They were used for descriptive model assessment and should not be interpreted as measures of prospective clinical predictive performance.

### Computational implementation and reproducibility

All analyses were conducted using open-source Python software. Bayesian inference was implemented in PyMC 5.24.1, with ArviZ 0.22.0 used for posterior summaries and convergence diagnostics. Numerical and statistical computations used NumPy 2.5.1, SciPy 1.18.0, and pandas 3.0.3, and figures were generated using Matplotlib 3.11.0. Analyses were run under Python 3.12.13 in a dedicated Conda environment named plos-ou on macOS/Darwin 25.5.0 with arm64 architecture.

The complete computational workflow includes scripts for rule-based construction of the longitudinal 𝓍 and 𝓃 series, deterministic preprocessing and quality control, simulation-based calibration benchmarking, hierarchical OU posterior inference, posterior predictive evaluation, model diagnostics, supplementary-table generation, and figure production.

All source code, documentation, input specifications, and executable workflows are openly available through the project GitHub repository:

https://github.com/shkim9391/Bayesian_Hybrid_OU_Branching_Precision_Medicine

A permanent archived release of the analysis pipeline is available through Zenodo:

https://zenodo.org/records/21447281

The archived materials include the analysis scripts, processed model inputs, posterior summaries, convergence and run metadata, software environment specification (environment.yml), and complete Conda package inventory (conda_package_versions.txt). Together, these resources provide the information required to reproduce the preprocessing, model fitting, simulation benchmarking, posterior predictive analyses, and figures reported in the manuscript.

### Ethics statement

This study analyzed previously published, publicly available data from the pediatric KMT2A-rearranged leukemia cohort reported by Ahlgren et al. No new human participants were recruited, no identifiable private information was accessed, and no new biological specimens were collected. All analyses were performed using de-identified data released by the original investigators in accordance with their institutional approvals and applicable ethical guidelines. Therefore, no additional institutional review board (IRB) approval or informed consent was required for this secondary analysis.

## Results

### Simulation-based evaluation of estimator performance

**Fig 2:**
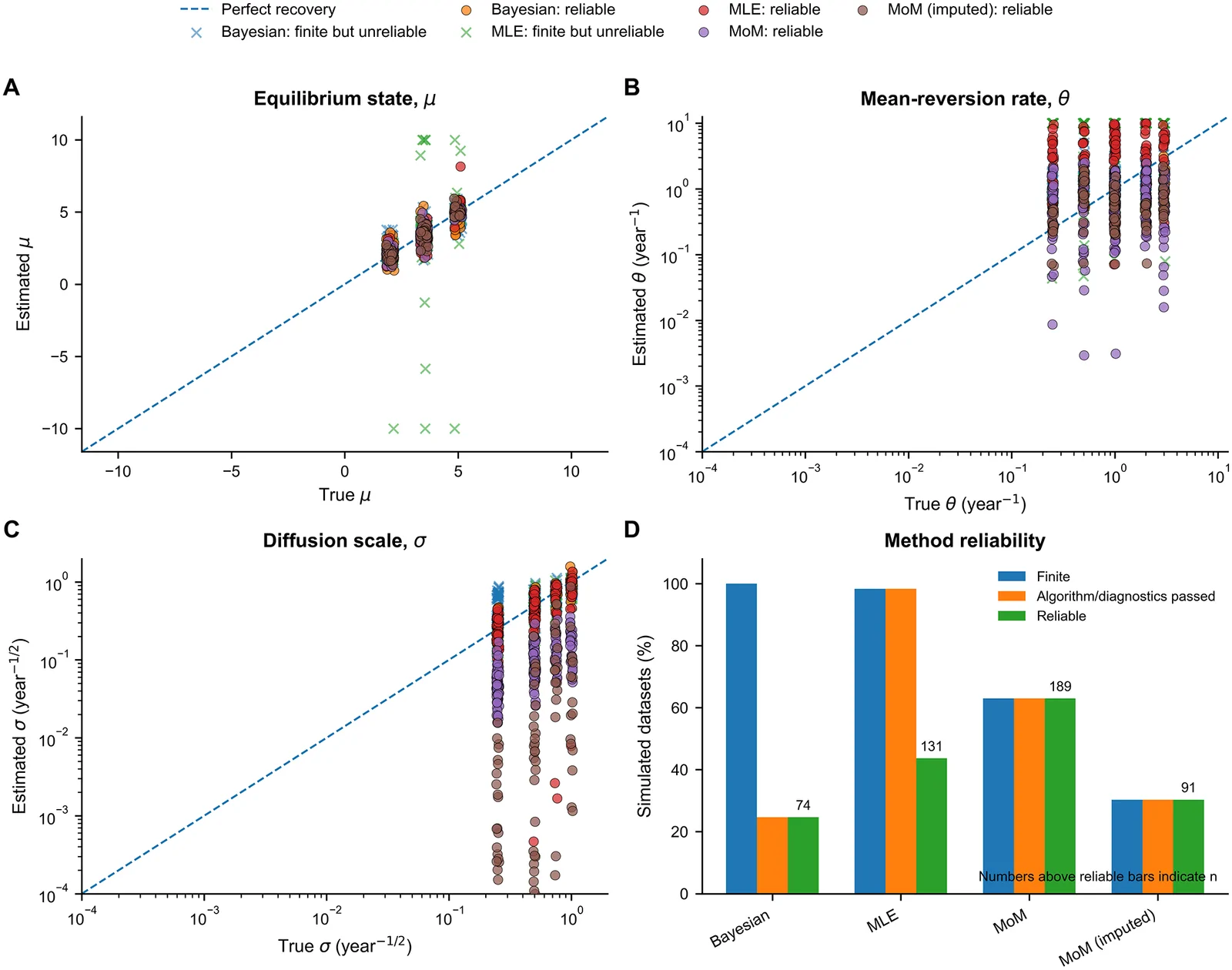
Simulation-based evaluation of OU parameter recovery under empirical KMT2A sampling schedules. Synthetic longitudinal datasets were generated using the observed sampling schedules from the pediatric KMT2A cohort with known generating parameters. (A) Recovery of the equilibrium state (μ). (B) Recovery of the mean-reversion rate (θ). (C) Recovery of the diffusion scale (σ). The dashed line indicates perfect parameter recovery. Bayesian estimates are classified according to posterior finiteness and diagnostic reliability, maximum-likelihood estimates according to optimizer success and boundary diagnostics, and method-of-moments (MoM) estimates according to identifiability criteria, with separate reporting for direct and imputed MoM estimation. (D) Percentage of simulated datasets yielding finite estimates, passing algorithm-specific diagnostic criteria, and meeting predefined reliability criteria. Numbers above the green bars indicate the number of reliable datasets. Quantitative parameter-recovery results and scenario-stratified estimator-reliability summaries are provided in S2 and S3 Tables.

The simulation benchmark evaluated recovery of μ, θ, and σ under empirical KMT2A sampling schedules using known generating parameters (Fig 2; S2 and S3 Tables). Recovery of μ was comparatively robust across estimation methods, whereas θ was difficult to identify under short and irregular schedules. Bayesian estimates of θ showed relatively small aggregate bias but weak correspondence between true and estimated values across scenarios, while maximum-likelihood estimates tended to show upward bias and method-of-moments estimates tended to show downward bias. Bayesian estimation provided the most favorable overall recovery of σ, whereas method-of-moments approaches frequently underestimated the diffusion scale.

Bayesian 95% credible-interval coverage was close to or above the nominal level for the principal parameters across the evaluated simulations, although interval widths increased under sparse sampling. Scenario-stratified results further showed that estimator reliability depended on observation number, temporal spacing, and the generating parameter combination rather than only on algorithmic convergence (S3 Table). These findings indicate that sparse clinical schedules support uncertainty-aware inference of some OU features, particularly μ, while providing weaker identification of θ and, in some settings, σ.

### Clinical-stage-associated OU parameter tendencies

The hierarchical OU direct-transition model was fitted to 47 patients contributing 242 positive-time transitions. Sampling was performed using four NUTS chains, and convergence was strong, with a maximum $\hat{R}$ of 1.003, minimum bulk and tail effective sample sizes exceeding 4,100, no divergent transitions, and no maximum tree-depth events. Group-level posterior summaries nevertheless showed broad uncertainty, indicating that computational convergence did not eliminate the limited identifiability imposed by sparse and uneven longitudinal sampling.

**Fig 3:**
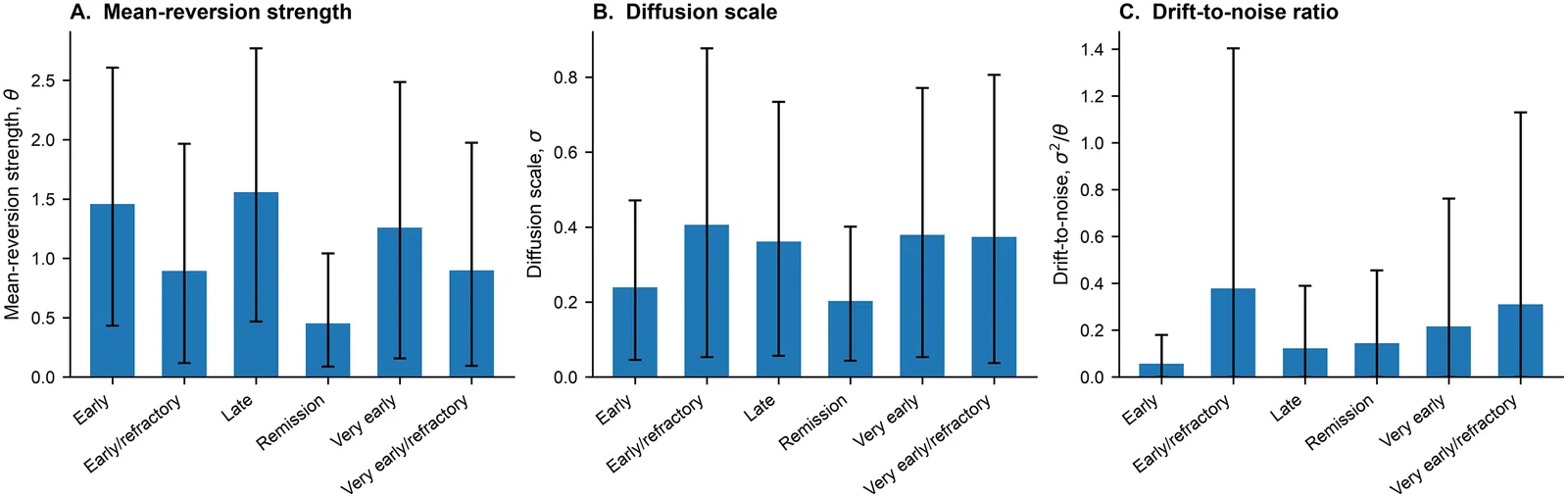
Group-level posterior summaries from the hierarchical OU direct-transition model. Bars represent posterior means and error bars indicate exact 95% highest-density intervals (HDIs). (A) Mean-reversion strength (θ). (B) Diffusion scale (σ). (C) Drift-to-noise ratio ($\sigma^{\text{2}}/\theta$). Posterior summaries were estimated jointly across all patients using a hierarchical Bayesian model with patient-specific equilibrium states and clinical-group-specific OU parameters. Broadly overlapping HDIs indicate substantial uncertainty in between-group differences under sparse longitudinal sampling. Numerical posterior summaries and pairwise posterior probabilities are provided in S4–S7 Tables.

Posterior means differed across clinical groups, but the 95% highest-density intervals overlapped substantially (Fig 3; S4–S7 Tables). Mean-reversion strength, θ, was highest on average in the Early and Late groups and lowest in Remission (Fig 3A). Diffusion scale, σ, was lower on average in Early and Remission and higher in the refractory and very-early groups (Fig 3B). The drift-to-noise ratio, $\sigma^{\text{2}}/\theta$, was largest on average in Early/refractory and Very early/refractory disease, but these groups also showed wide posterior intervals and contained relatively few patients (Fig 3C). Accordingly, the results support descriptive stage-associated tendencies rather than sharply separated dynamical regimes.

Pairwise posterior probabilities provided additional quantitative comparisons among clinical groups (S5–S7 Tables), but the broad overlap among posterior distributions indicated limited evidence for definitive group ordering. The inferred parameters should therefore be interpreted as effective statistical summaries of mean reversion and stochastic variability under the OU model rather than as direct evidence of stabilizing selection or specific biological mechanisms.

### Patient-specific evolutionary trajectories

**Fig 4:**
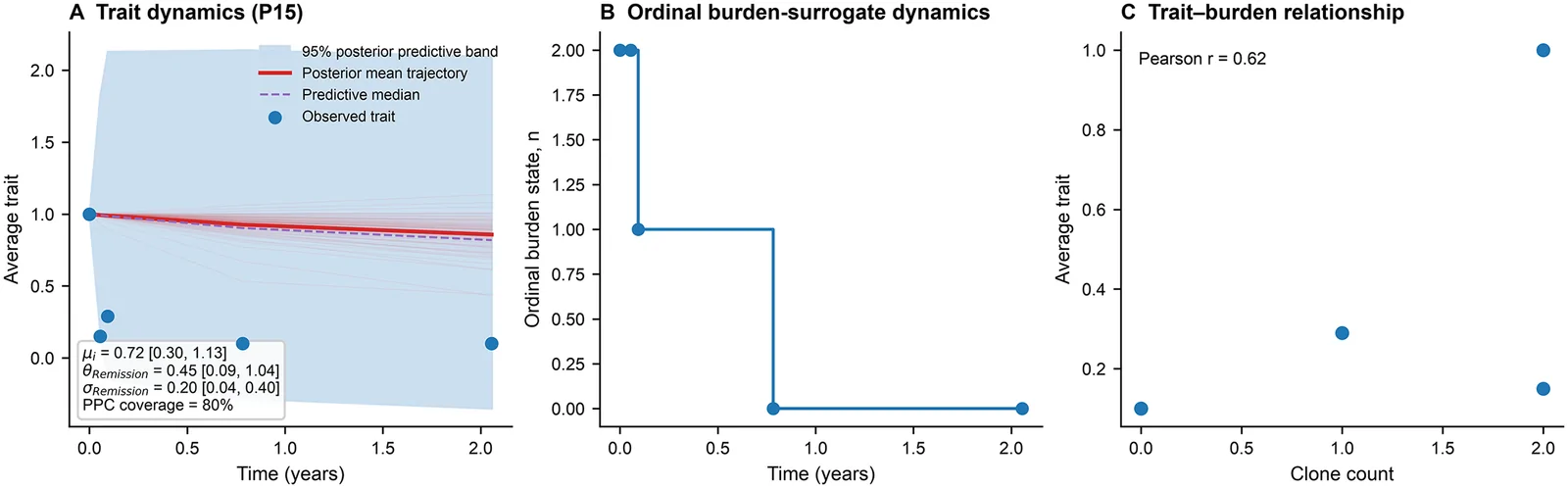
Patient-specific posterior predictive reconstruction for an exemplar patient (P15). (A) Longitudinal average trait values (blue circles) together with the posterior mean trajectory (solid red line), posterior predictive median (dashed purple line), and 95% posterior predictive interval (blue shading) generated from the hierarchical OU direct-transition model. The inset summarizes the patient-specific posterior equilibrium state ($\mu_{\text{𝒾}}$), group-level mean-reversion strength (θ), group-level diffusion scale (σ), and posterior predictive coverage. (B) Longitudinal dynamics of the annotation-derived ordinal burden surrogate, 𝓃. (C) Exploratory relationship between the ordinal burden surrogate and average trait values evaluated at corresponding observation times. Corresponding posterior predictive figures for all modeled patients are provided in S2 Fig, and patient-level predictive summaries are reported in S9 Table.

Patient-specific equilibrium states $\mu_{\text{𝒾}}$ were estimated hierarchically, whereas $\theta_{g}$ and $\sigma_{g}$ were shared among patients within each clinical group. This structure allowed individual trajectories to borrow information from the cohort while retaining patient-specific posterior uncertainty (S8 Table). Fig 4 illustrates the fitted dynamics for patient P15, a remission-group patient with five longitudinal trait observations. The posterior mean equilibrium state was approximately 0.72 (95% HDI, 0.30–1.13). The corresponding remission-group posterior estimates were θ=0.45 (95% HDI, 0.09–1.04) and σ=0.20 (95% HDI, 0.04–0.40).

The posterior mean trajectory changed gradually from the initial observation, whereas the posterior predictive interval remained broad because it incorporated uncertainty in the patient-specific equilibrium state, the group-level OU parameters, the process variance, and the residual transition scale (Fig 4A). Four of the five observed trait values fell within the corresponding 95% posterior predictive intervals, yielding patient-level coverage of 80%. The annotation-derived burden surrogate declined from 𝓃=2 to 𝓃=1 and subsequently to 𝓃=0 over the observed period (Fig 4B). The relationship between the ordinal burden surrogate and average trait was positive in this example, although it was based on only a small number of paired observations and was treated as exploratory (Fig 4C).

Corresponding posterior predictive figures for all 47 modeled patients are provided in S2 Fig. These analyses revealed substantial heterogeneity in the observed trajectories, even among patients sharing the same group-level $\theta_{g}$ and $\sigma_{g}$. Patient-level posterior summaries and predictive performance measures are reported in S8–S10 Tables.

### Cohort-level posterior predictive diagnostics

**Fig 5:**
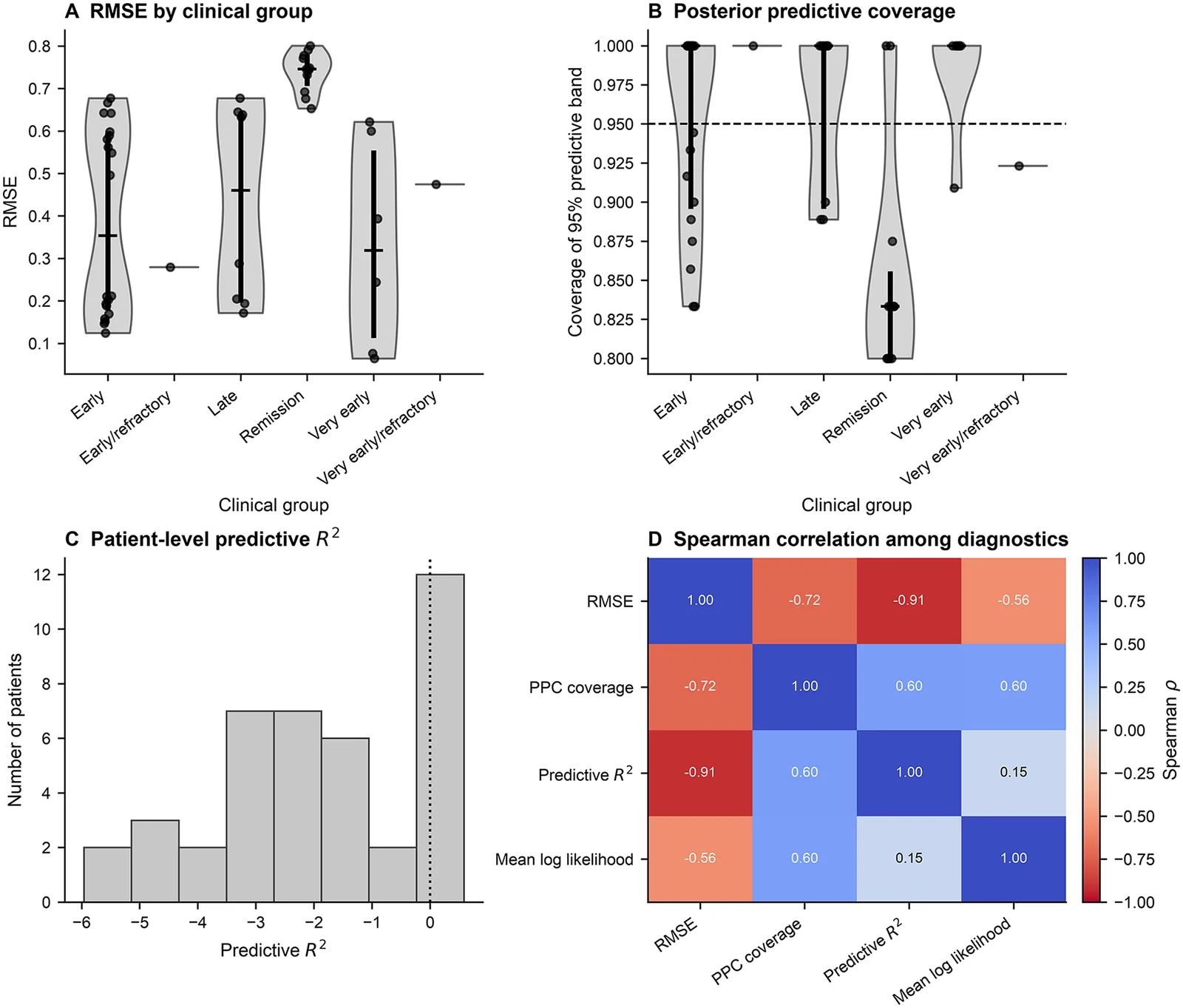
Cohort-level diagnostic evaluation of the hierarchical OU direct-transition model. (A) Distribution of patient-level root mean squared error (RMSE) stratified by clinical group. (B) Patient-level coverage of the nominal 95% posterior predictive interval by clinical group; the dashed horizontal line denotes the nominal 95% coverage level. (C) Distribution of patient-level predictive $R^{\text{2}}$ values computed from posterior-mean deterministic trajectories. Negative predictive $R^{\text{2}}$ values indicate that posterior-mean trajectories predicted individual patient trajectories less accurately than the patient-specific observed mean and should not be interpreted as evidence of poor posterior convergence or invalid parameter inference. (D) Spearman rank correlations among diagnostic measures, including RMSE, posterior predictive coverage, predictive $R^{\text{2}}$, and mean transition log-likelihood. Detailed patient-level, group-level, and correlation-based diagnostic summaries are reported in S10–S12 Tables.

Patient-level posterior predictive evaluation showed generally high uncertainty-aware coverage across the cohort (Fig 5; S10–S12 Tables). Mean coverage of the nominal 95% posterior predictive interval was approximately 94%, and the median patient-level coverage was 100%. Coverage varied by clinical group and was lowest in Remission, which also exhibited comparatively high root mean squared error in the posterior-mean trajectory reconstruction (Fig 5A,B). The Early/refractory and Very early/refractory groups each contained only one modeled patient, and their displayed values should therefore not be interpreted as group-level distributions.

Predictive $R^{\text{2}}$, calculated from the posterior-mean deterministic trajectory, was evaluable for 41 patients; it was undefined for six patients with no within-patient variation in the observed trait. Predictive $R^{\text{2}}$ was negative for many evaluable patients (Fig 5C). These negative values indicate that the posterior-mean trajectory reconstructed the observed patient series less accurately than a constant model based on that patient’s observed mean. They do not indicate failed MCMC convergence or invalidate posterior inference for $\mu_{\text{𝒾}}$, $\theta_{g}$, or $\sigma_{g}$. Instead, they demonstrate that accurate point reconstruction of short, irregular trajectories is more difficult than obtaining broad posterior predictive coverage.

The diagnostic measures captured related but distinct aspects of model behavior (Fig 5D). RMSE was strongly negatively correlated with predictive $R^{\text{2}}$ and posterior predictive coverage, whereas predictive $R^{\text{2}}$ was only weakly associated with mean transition log-likelihood. Together, these findings distinguish three separate properties of the analysis: posterior parameter inference, uncertainty-aware predictive coverage, and point-trajectory reconstruction. The model provided stable posterior inference and broadly calibrated predictive intervals, but its posterior-mean trajectories did not reproduce all short-timescale fluctuations in individual patients. This distinction is consistent with the primary purpose of the framework: estimation of effective dynamical characteristics and their uncertainty, rather than precise forecasting of future patient trajectories.

## Discussion

This study applied Bayesian-calibrated stochastic modeling to characterize sparse longitudinal tumor trajectories in pediatric leukemia. Rather than introducing a new stochastic process, the primary objective was to evaluate whether an interpretable Ornstein–Uhlenbeck (OU) framework could provide useful uncertainty-aware summaries of effective mean reversion, equilibrium state, and stochastic variability across clinically defined disease stages. The revised analyses show that this framework can yield stable posterior inference and broadly calibrated predictive intervals, while also making clear the limits imposed by short, irregularly sampled patient trajectories.

The group-level posterior summaries suggested stage-associated differences in effective OU dynamics, but these differences were accompanied by substantial uncertainty. Mean-reversion strength was higher on average in the Early and Late groups and lower in Remission, whereas diffusion and drift-to-noise tended to be greater in refractory and very-early groups. However, the 95% highest-density intervals overlapped broadly across clinical categories, and some refractory groups contained very few modeled patients. The results therefore do not support sharply separated stage-specific dynamical regimes or direct claims about restoration or loss of biological constraints. A more appropriate interpretation is that the clinical groups exhibited different posterior tendencies in effective mean reversion and stochastic variability under the fitted OU model.

The OU process is particularly suitable for the present application because pediatric leukemia progression is not expected to evolve as unrestricted random diffusion. Instead, disease dynamics are influenced by developmental programs, therapeutic intervention, immune surveillance, and other regulatory processes that constrain trajectories toward transient effective equilibrium states. Compared with Brownian motion, which assumes unconstrained variance growth over time, the OU process provides a parsimonious representation of stochastic fluctuations around such constrained dynamics while remaining identifiable under sparse longitudinal sampling.

This distinction is especially important for biological interpretation. The OU parameters are statistical summaries of the observed longitudinal process. A larger θ indicates stronger effective return toward an inferred equilibrium state, whereas a larger σ indicates greater inferred stochastic variability. These quantities may be compatible with biological processes such as treatment-associated stabilization, fluctuating clonal composition, or heterogeneous selective pressures, but they do not by themselves identify the mechanisms responsible for those patterns. Accordingly, expressions such as “stabilizing constraint,” “evolutionary instability,” or “phenotypic dispersion” should be treated as hypotheses motivated by the fitted dynamics rather than direct mechanistic conclusions.

Bayesian calibration was particularly valuable because uncertainty was substantial even when computational convergence was excellent. The hierarchical direct-transition model achieved $\hat{R}$ values close to 1, high effective sample sizes, and no divergent transitions, yet several group-level parameters retained wide posterior intervals. This contrast demonstrates that sampler convergence and parameter identifiability are separate issues. The posterior computation was stable, but the available data did not always contain enough longitudinal information to distinguish precisely among plausible values of θ, σ, and their derived ratios. Reporting the full posterior distributions therefore provides a more accurate representation of the evidence than reporting point estimates alone.

The hierarchical formulation also allowed patient-specific equilibrium states to be estimated while sharing information about θ and σ within clinical groups. This partial pooling is appropriate for sparse clinical data because separate patient-level fits would be highly unstable for individuals with only a few observations. At the same time, shared group-level parameters do not imply that all patients within a category followed the same trajectory. The all-patient posterior predictive analyses showed marked heterogeneity in observed dynamics, including substantial variation among patients assigned to the same clinical group. The model should therefore be viewed as estimating group-associated dynamical tendencies while retaining patient-specific uncertainty, not as defining a single deterministic trajectory for each stage.

### Prediction, trajectory reconstruction, and dynamical inference

The revised diagnostics clarify three concepts that should not be conflated: posterior predictive coverage, point-trajectory reconstruction, and inference about effective dynamical parameters. Posterior predictive coverage was generally high across the cohort, with mean coverage close to the nominal 95% level. This indicates that the model’s predictive distributions were usually broad enough to encompass the observed trajectories. In contrast, predictive $R^{\text{2}}$ values based on posterior-mean trajectories were negative for many patients, showing that a single posterior-mean trajectory often reproduced short, irregular patient series less accurately than the patient-specific observed mean.

These findings are not contradictory. Posterior predictive intervals incorporate process variability, residual transition variability, and posterior uncertainty in the model parameters, whereas predictive $R^{\text{2}}$ evaluates the accuracy of a single point trajectory. A model can therefore provide reasonable uncertainty-aware coverage while showing limited ability to reconstruct each observed fluctuation using its posterior mean. Negative predictive $R^{\text{2}}$ values do not indicate invalid posterior inference or poor MCMC convergence. Rather, they emphasize that sparse stochastic trajectories are difficult to reproduce precisely and that the primary contribution of the framework is uncertainty-aware inference of effective dynamical characteristics, not deterministic forecasting.

This distinction also defines what can and cannot be inferred from the present analysis. The model can estimate posterior distributions for patient-specific equilibrium states, clinical-group mean-reversion strength, diffusion scale, and derived quantities such as drift-to-noise. It can reconstruct broad trajectory tendencies and quantify the range of plausible future or replicated observations under the fitted model. It cannot establish a unique biological mechanism, determine the direction of causality between clinical stage and inferred dynamics, or provide sufficiently precise individual forecasts for direct clinical decision-making.

### Simulation-based validation and identifiability

The simulation benchmark further demonstrated that identifiability depended strongly on both the parameter and the sampling schedule. The equilibrium state μ was generally recovered more consistently than θ, while recovery of σ varied across methods and scenarios. Mean-reversion strength was particularly difficult to estimate from short or irregular series because several combinations of μ, θ, and σ can produce similar transitions over limited follow-up. Bayesian inference reduced some forms of instability and supplied credible intervals, but it did not eliminate the information limits inherent in sparse data.

The comparison with maximum-likelihood and method-of-moments estimation also showed that no method was uniformly superior. Maximum-likelihood estimates were often finite but could approach parameter boundaries or show substantial bias, particularly for θ. Method-of-moments estimates performed well when identifiable but were not available for all sparse schedules and tended to underestimate some parameters. Bayesian estimates remained finite and supported explicit uncertainty quantification, although only a subset met the strict reliability criteria under the most difficult scenarios. These results support the use of Bayesian calibration for this application while discouraging claims that it guarantees precise recovery under all sampling conditions.

### Alternative modeling approaches

The OU process was selected because it provides an interpretable balance between directional pull toward an equilibrium and stochastic variation while requiring relatively few parameters. This simplicity is advantageous for sparse longitudinal data, but alternative models could capture features that the OU process cannot. Brownian motion would represent unconstrained diffusion and may be appropriate when mean reversion is unsupported. Switching OU or hidden Markov models could represent transitions among latent dynamical states. Nonlinear stochastic differential equations could capture state-dependent drift or diffusion, and jump or Lévy processes could represent abrupt changes. Explicit branching models could more directly describe clonal expansion and extinction when sufficiently detailed lineage data are available.

These alternatives generally introduce additional parameters or latent structures that may be difficult to identify with the present sampling density. The OU framework should therefore be viewed as a parsimonious first-order representation rather than a complete model of leukemia evolution. Future model comparison should assess whether the additional flexibility of switching, nonlinear, or branching formulations produces meaningfully improved predictive performance and biological interpretability without overwhelming the available information.

These approaches should therefore be regarded as complementary rather than competing methodologies. The OU framework provides an interpretable baseline representation of continuous constrained dynamics that can be extended when richer longitudinal information becomes available. For example, abrupt evolutionary transitions may be represented through Lévy-type jump processes, lineage diversification through branching processes, and discrete disease-state transitions through hidden Markov models. The choice among these models should be guided by biological questions, data availability, and model identifiability rather than by any single universally optimal stochastic formulation.

### Generalizability

Although the present study focused on pediatric KMT2A-rearranged leukemia, the statistical framework itself is not disease specific. Because inference relies only on irregular longitudinal measurements of an observed trait, the same Bayesian OU framework could be applied to other pediatric leukemias, adult hematologic malignancies, solid tumors, or other longitudinal biological datasets exhibiting constrained stochastic dynamics.

Nevertheless, successful application to other disease settings will require evaluation of the underlying modeling assumptions, sampling density, and observation process. Independent validation across multiple cohorts and experimental platforms will therefore be important for assessing the broader applicability of the framework.

### Methodological implications

From a statistical perspective, the principal contribution of this study is the demonstration that Bayesian calibration provides a practical framework for analyzing sparse and irregular longitudinal tumor trajectories using an OU process. Although the fitted model is intentionally parsimonious, the hierarchical Bayesian formulation enables stable posterior computation, explicit uncertainty quantification, and principled estimation of patient-specific equilibrium states while borrowing statistical strength across patients within predefined clinical groups. The simulation benchmark further showed that computational convergence does not necessarily imply parameter identifiability, emphasizing the importance of reporting posterior uncertainty rather than relying solely on point estimates.

The framework is intended primarily as an uncertainty-aware statistical modeling approach rather than a predictive algorithm or mechanistic model. The inferred OU parameters summarize effective longitudinal dynamics under the assumed stochastic process and should therefore be interpreted as statistical descriptors of the observed trajectories rather than direct measurements of biological regulatory mechanisms. This distinction is particularly important for sparse clinical datasets, in which uncertainty arising from limited sampling can substantially exceed uncertainty associated with numerical posterior computation.

### Biological observations

Within the pediatric KMT2A-rearranged leukemia cohort, the hierarchical OU model identified stage-associated tendencies in effective mean reversion and stochastic variability. Mean-reversion strength tended to be higher in the Early and Late groups, whereas diffusion and drift-to-noise tended to be greater in refractory and very-early disease groups. However, these differences were accompanied by broad posterior uncertainty and substantial overlap among clinical groups. Consequently, the present results do not support sharply separated dynamical regimes but instead indicate modest differences in effective statistical behavior under the fitted OU model.

These observations should be interpreted cautiously. Parameters such as the equilibrium state, mean-reversion rate, and diffusion scale provide quantitative summaries of longitudinal tumor dynamics but do not directly identify the biological mechanisms responsible for those dynamics. Multiple biological processes—including treatment response, clonal evolution, developmental constraints, immune interactions, and measurement variability—may contribute to similar statistical behavior. Accordingly, the present findings should be viewed as hypothesis-generating descriptions of longitudinal disease dynamics rather than definitive mechanistic evidence.

### Future applications

Building upon this generalizability, future methodological developments may extend the present framework through treatment-dependent covariates, nonlinear stochastic dynamics, hidden-state transitions, explicit branching processes, or jump-process formulations. Application to these settings will nevertheless require independent evaluation of the underlying modeling assumptions, sampling density, and observation process.

The OU process should also be viewed as a parsimonious first-order representation of longitudinal tumor evolution rather than a complete description of all evolutionary processes. Future extensions may incorporate treatment-dependent covariates, nonlinear stochastic dynamics, hidden-state transitions, explicit branching processes, or jump-process formulations to capture abrupt evolutionary changes when supported by richer longitudinal data. Comparative evaluation of these alternative stochastic models will help determine the appropriate balance between biological realism, statistical identifiability, and predictive performance across different experimental settings.

### Limitations

Several limitations should be acknowledged. First, the analysis used a Gaussian direct-transition model, which may not adequately represent bounded traits, multimodal state distributions, abrupt clonal shifts, or strongly nonlinear dynamics. Second, parameter identifiability remained limited for patients with few observations or short follow-up, particularly for the mean-reversion parameter (θ). Third, the predefined clinical groups were uneven in size, with some refractory categories represented by only a single modeled patient; estimates for these groups should therefore be regarded as exploratory.

Fourth, the residual transition term and the OU diffusion term can both contribute to observed variability, making their biological interpretation non-unique. Fifth, clinical-stage assignments summarize complex disease histories and do not explicitly account for treatment exposure, molecular subtype, lineage composition, or other time-varying biological covariates. Sixth, the analysis modeled derived longitudinal traits and an annotation-derived ordinal burden surrogate rather than jointly modeling the underlying genomic, transcriptomic, and clinical measurements. Finally, the exploratory associations between the modeled trait and the burden surrogate were based on relatively small numbers of paired observations and should therefore be interpreted cautiously.

## Conclusion

Bayesian-calibrated Ornstein–Uhlenbeck modeling provides a practical and interpretable framework for uncertainty-aware analysis of sparse longitudinal tumor dynamics in pediatric leukemia. The hierarchical Bayesian formulation yielded stable posterior computation, explicit uncertainty quantification, and broadly calibrated posterior predictive intervals while identifying stage-associated tendencies in effective mean reversion and stochastic variability.

The principal contribution of this study is methodological rather than predictive. Rather than providing deterministic forecasts or direct mechanistic inference, the framework offers an interpretable statistical representation of longitudinal tumor dynamics together with explicit characterization of inferential uncertainty. Broadly overlapping posterior intervals, uneven clinical-group sizes, and limited patient-level trajectory reconstruction appropriately constrain biological interpretation, while demonstrating how Bayesian stochastic modeling can distinguish identifiable dynamical features from those that remain unresolved under sparse longitudinal sampling.

## Supporting information

S1 Fig Longitudinal sampling characteristics of the KMT2A-rearranged leukemia cohort. (PDF)

S2 Fig Posterior predictive dynamics for all 47 modeled patients. (PDF)

S1 Table Cohort-level and patient-level longitudinal sampling characteristics. (XLSX)

S2 Table Simulation-level OU parameter estimates for Bayesian, MLE, MoM, and imputed MoM methods. (CSV)

S3 Table Scenario-stratified estimator robustness and overall method summaries. (XLSX)

S4 TableGroup-level posterior summaries for θ, σ, $\sigma^{2}/\theta$, and $\sigma^{2}/(2\theta )$.(CSV)

S5 TablePairwise posterior probabilities and differences for group-level θ.(CSV)

S6 TablePairwise posterior probabilities and differences for group-level σ.(CSV)

S7 TablePairwise posterior probabilities and differences for $\sigma^{2}/\theta$.(CSV)

S8 Table Patient-specific posterior summaries for equilibrium state *μ_i_*. (CSV)

S9 Table Patient-level posterior predictive summaries for all 47 modeled patients. (CSV)

S10 TablePatient-level RMSE, posterior predictive coverage, interval width, predictive $R^{2}$, and mean log-likelihood per transition.(CSV)

S11 Table Clinical-group summaries with bootstrap 95% confidence intervals. (CSV)

S12 TableSpearman correlations and associated p values among model diagnostics.(CSV)

S1 Data Cleaned longitudinal workbook used for simulation benchmarking and series preparation. (XLSX)

S2 Data Tidy direct-transition input with columns *Patient_ID*, *series*, *t*, and *value.* (CSV)

S3 Data Quality-control and metadata table from longitudinal-series preparation. (CSV)

S4 Data Patient-to-clinical-group mapping used in the hierarchical OU model. (CSV)

S5 Data Definitions and true parameter settings for all simulation scenarios. (CSV)

S6 Data Simulation performance metrics, including bias, RMSE, and interval coverage. (CSV)

S7 Data Finite-estimate, diagnostic-success, and reliability indicators. (CSV)

S8 Data ArviZ *InferenceData* object containing hierarchical OU posterior samples. (NC)

S9 Data Comprehensive posterior summary for group-level, global, and patient-specific parameters. (CSV)

S10 Data Ordered mapping between group indices and clinical-group labels. (CSV)

S11 Data Mapping between patients, groups, group indices, and posterior variable names. (CSV)

S12 DataSummary of $\hat{R}$, effective sample sizes, divergences, and tree-depth events.(CSV)

S13 Data Run configuration, cohort dimensions, software settings, and output provenance. (CSV)

## References

[1] Ahlgren L, Pilheden M, Sturesson H, Song G, Walsh MP, Yang M, et al. The genomic landscape of relapsed infant and childhood KMT2A-rearranged acute leukemia. Nat Commun. 2025;16(1):8964. doi: 10.1038/s41467-025-64190-8 41062506 PMC12508131

[2] Coorens THH, Collord G, Treger TD, Adams S, Mitchell E, Newman B, et al. Clonal origin of KMT2A wild-type lineage-switch leukemia following CAR-T cell and blinatumomab therapy. Nat Cancer. 2023;4(8):1095–101. doi: 10.1038/s43018-023-00604-0 37474833 PMC10447231

[3] Richter-Pechańska P, Kunz JB, Rausch T, Erarslan-Uysal B, Bornhauser B, Frismantas V, et al. Pediatric T-ALL type-1 and type-2 relapses develop along distinct pathways of clonal evolution. Leukemia. 2022;36(7):1759–68. doi: 10.1038/s41375-022-01587-0 35585141 PMC9252914

[4] Costea J, Rauwolf KK, Zafferani P, Rausch T, Mathioudaki A, Zaugg J, et al. Role of stem-like cells in chemotherapy resistance and relapse in pediatric T-cell acute lymphoblastic leukemia. Nat Commun. 2025;16(1):5413. doi: 10.1038/s41467-025-61222-1 40579412 PMC12205070

[5] Zhang J, Duan Y, Wu P, Chang Y, Wang Y, Hu T, et al. Clonal evolution dissection reveals that a high MSI2 level promotes chemoresistance in T-cell acute lymphoblastic leukemia. Blood. 2024;143(4):320–35. doi: 10.1182/blood.2023020490 37801708

[6] Lambo S, Trinh DL, Ries RE, Jin D, Setiadi A, Ng M, et al. A longitudinal single-cell atlas of treatment response in pediatric AML. Cancer Cell. 2023;41(12):2117-2135.e12. doi: 10.1016/j.ccell.2023.10.008 37977148

[7] Pang Y. Measuring longitudinal genome-wide clonal evolution of pediatric acute lymphoblastic leukemia at single-cell resolution. bioRxiv. 2025. doi: 10.1101/2025.03.19.644196

[8] Fabre MA, de Almeida JG, Fiorillo E, Mitchell E, Damaskou A, Rak J, et al. The longitudinal dynamics and natural history of clonal haematopoiesis. Nature. 2022;606(7913):335–42. doi: 10.1038/s41586-022-04785-z 35650444 PMC9177423

[9] Robertson NA, Latorre-Crespo E, Terradas-Terradas M, Lemos-Portela J, Purcell AC, Livesey BJ, et al. Longitudinal dynamics of clonal hematopoiesis identifies gene-specific fitness effects. Nat Med. 2022;28(7):1439–46. doi: 10.1038/s41591-022-01883-3 35788175 PMC9307482

[10] Ramazzotti D. LACE: inference of cancer evolution models from longitudinal single-cell sequencing data. J Comput Sci. 2022;58:101523. doi: 10.1016/j.jocs.2021.101523

[11] Blomberg SP, Rathnayake SI, Moreau CM. Beyond Brownian Motion and the Ornstein-Uhlenbeck Process: Stochastic Diffusion Models for the Evolution of Quantitative Characters. Am Nat. 2020;195(2):145–65. doi: 10.1086/706339 32017624

[12] de Souza Silva CC. Phenotypic evolution as an Ornstein–Uhlenbeck process. Phys Rev E. 2023;107:024417. doi: 10.1103/physreve.107.024417 36932534

[13] Pienaar J, Bartoszek K, Brahmantio B, Fierst JL, Fuentes-González JA, Hansen TF, et al. Phylogenetic comparative methods for studying adaptation: the adaptation-inertia framework. J Evol Biol. 2026;39(1):1–17. doi: 10.1093/jeb/voaf113 40990933 PMC13237619

[14] Oksendal B. Stochastic Differential Equations: An Introduction with Applications. Berlin: Springer. 2003. doi: 10.1007/978-3-642-14394-6

[15] Liu J, Jiang P, Lu Z, Yu Z, Qian P. Decoding leukemia at the single-cell level: clonal architecture, classification, microenvironment, and drug resistance. Exp Hematol Oncol. 2024;13(1):12. doi: 10.1186/s40164-024-00479-6 38291542 PMC10826069

[16] Lee ND, Bozic I. Inferring parameters of cancer evolution in chronic lymphocytic leukemia. PLoS Comput Biol. 2022;18(11):e1010677. doi: 10.1371/journal.pcbi.1010677 36331987 PMC9668150

[17] Patruno L, Milite S, Bergamin R, Calonaci N, D’Onofrio A, Anselmi F, et al. A Bayesian method to infer copy number clones from single-cell RNA and ATAC sequencing. PLoS Comput Biol. 2023;19(11):e1011557. doi: 10.1371/journal.pcbi.1011557 37917660 PMC10645363

[18] Vehtari A, Gelman A, Gabry J. Practical Bayesian model evaluation using leave-one-out cross-validation and WAIC. Stat Comput. 2017;27:1413–32.

[19] Nishio M, Arakawa A. Performance of Hamiltonian Monte Carlo and No-U-Turn Sampler for estimating genetic parameters and breeding values. Genet Sel Evol. 2019;51(1):73. doi: 10.1186/s12711-019-0515-1 31823719 PMC6902603

[20] Yu J. Bias in the estimation of the mean-reversion parameter in continuous-time models. J Econom. 2012;169:114–22. doi: 10.1016/j.jeconom.2012.01.004

[21] Kim S-H. A hybrid Ornstein-Uhlenbeck-Branching framework unifies microbial and pediatric tumor evolution. Front Oncol. 2026;16:1727973. doi: 10.3389/fonc.2026.1727973 41783443 PMC12954049

[22] Kim S-H. Modeling constrained tumor evolution through hybrid Ornstein-Uhlenbeck and branching dynamics. iScience. 2026;29(8):116970. doi: 10.1016/j.isci.2026.116970 42564904 PMC13444672

[23] Whiting FJH, Mossner M, Gabbutt C, Kimberley C, Barnes CP, Baker A-M, et al. Quantitative measurement of phenotype dynamics during cancer drug resistance evolution using genetic barcoding. Nat Commun. 2025;16(1):5282. doi: 10.1038/s41467-025-59479-7 40541962 PMC12181405

[24] Baele G, Ji X, Hassler GW, McCrone JT, Shao Y, Zhang Z, et al. BEAST X for Bayesian phylogenetic, phylogeographic and phylodynamic inference. Nat Methods. 2025;22(8):1653–6. doi: 10.1038/s41592-025-02751-x 40624354 PMC12328226

[25] Staicova D. Modern Bayesian Sampling Methods for Cosmological Inference: A Comparative Study. Universe. 2025;11(2):68. doi: 10.3390/universe11020068

