# Supplementary material for: Stage-specific evolutionary dynamics in pediatric leukemia inferred using Bayesian stochastic modeling
Source: PLoS One. 2026 Sep 11;21(9):e0357755. doi: 10.1371/journal.pone.0357755 (PMC13568502; doi:10.1371/journal.pone.0357755)

Longitudinal sampling characteristics of the KMT2A-rearranged leukemia cohort

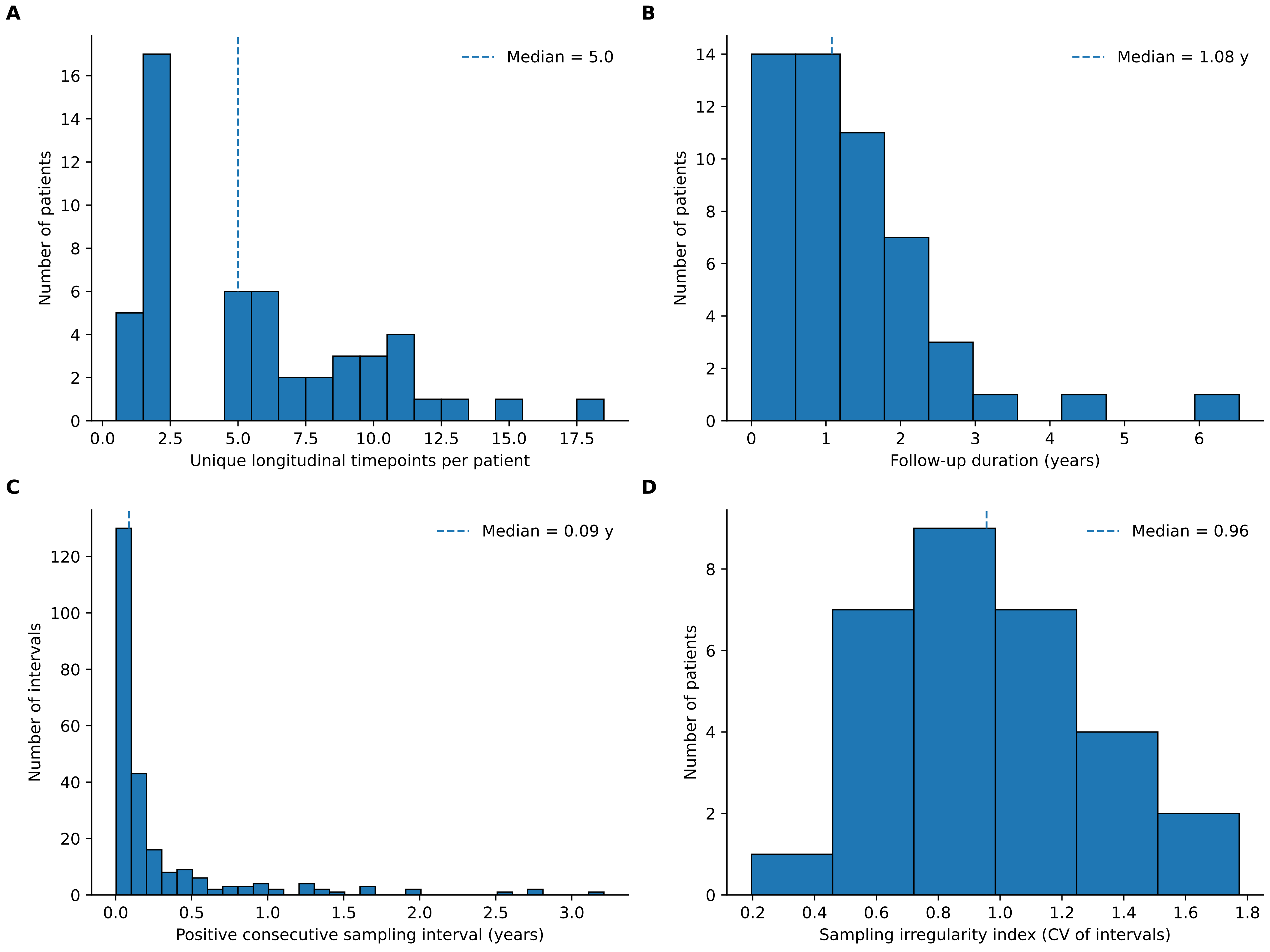

Supplement: Supplementary file 1 [file pone.0357755.s001.pdf]
