# Supplementary material for: Stage-specific evolutionary dynamics in pediatric leukemia inferred using Bayesian stochastic modeling
Source: PLoS One. 2026 Sep 11;21(9):e0357755. doi: 10.1371/journal.pone.0357755 (PMC13568502; doi:10.1371/journal.pone.0357755)
Supplement: Supplementary file 2 [file pone.0357755.s002.pdf]

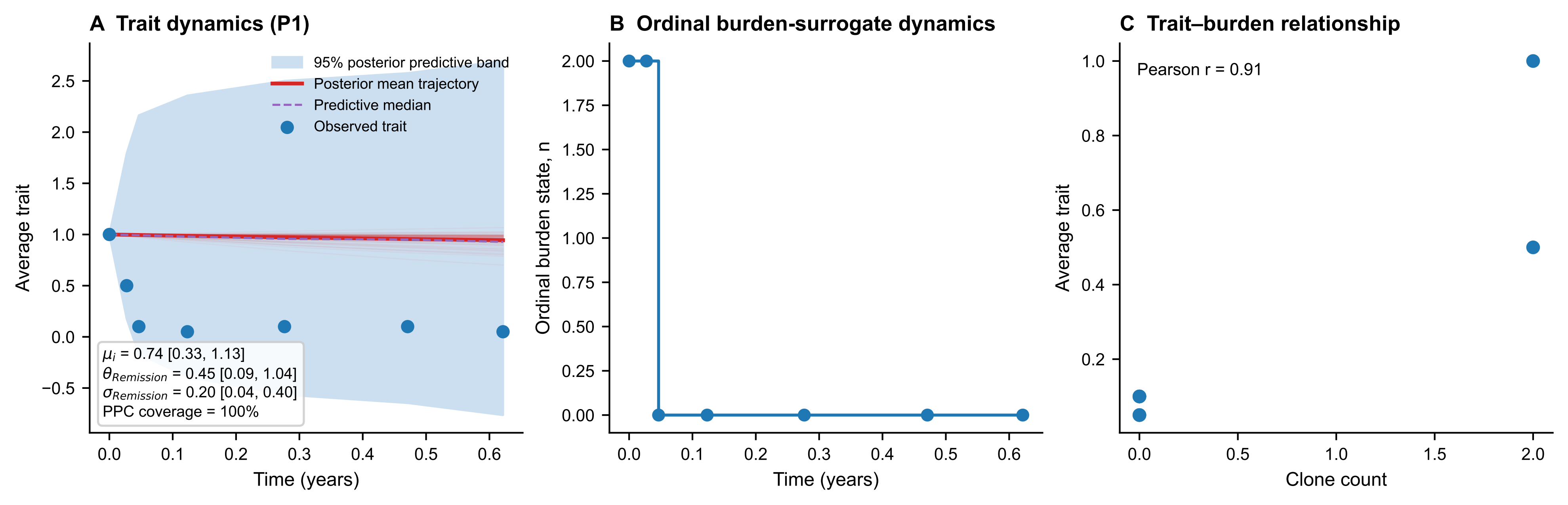

A Trait dynamics (P1)
95% posterior predictive band
Posterior mean trajectory
Predictive median
Observed trait
μ_i = 0.74 [0.33, 1.13]
$\theta_{Remission}$ = 0.45 [0.09, 1.04]
$\sigma_{Remission}$ = 0.20 [0.04, 0.40]
PPC coverage = 100%
Average trait
Time (years)
B Ordinal burden-surrogate dynamics
Ordinal burden state, n
Time (years)
C Trait–burden relationship
Pearson r = 0.91
Average trait
Clone count

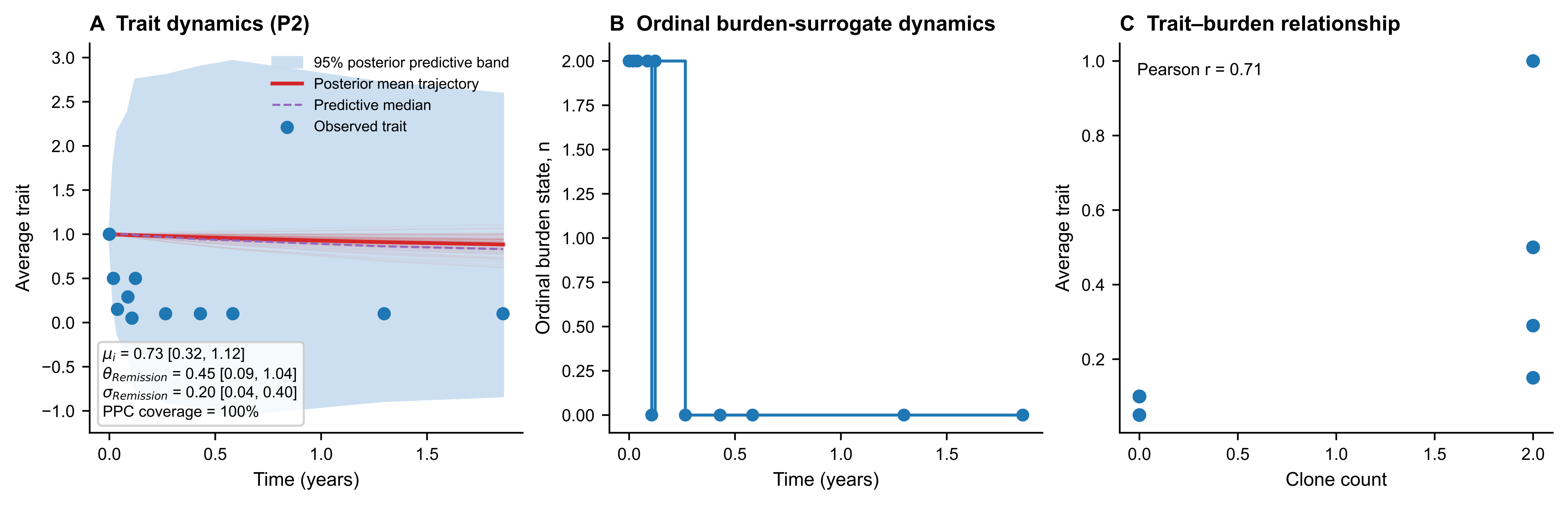

A Trait dynamics (P2)
95% posterior predictive band
Posterior mean trajectory
Predictive median
Observed trait
μ_i = 0.73 [0.32, 1.12]
$\theta_{Remission}$ = 0.45 [0.09, 1.04]
$\sigma_{Remission}$ = 0.20 [0.04, 0.40]
PPC coverage = 100%
Average trait
Time (years)
B Ordinal burden-surrogate dynamics
Ordinal burden state, n
Time (years)
C Trait–burden relationship
Pearson r = 0.71
Average trait
Clone count

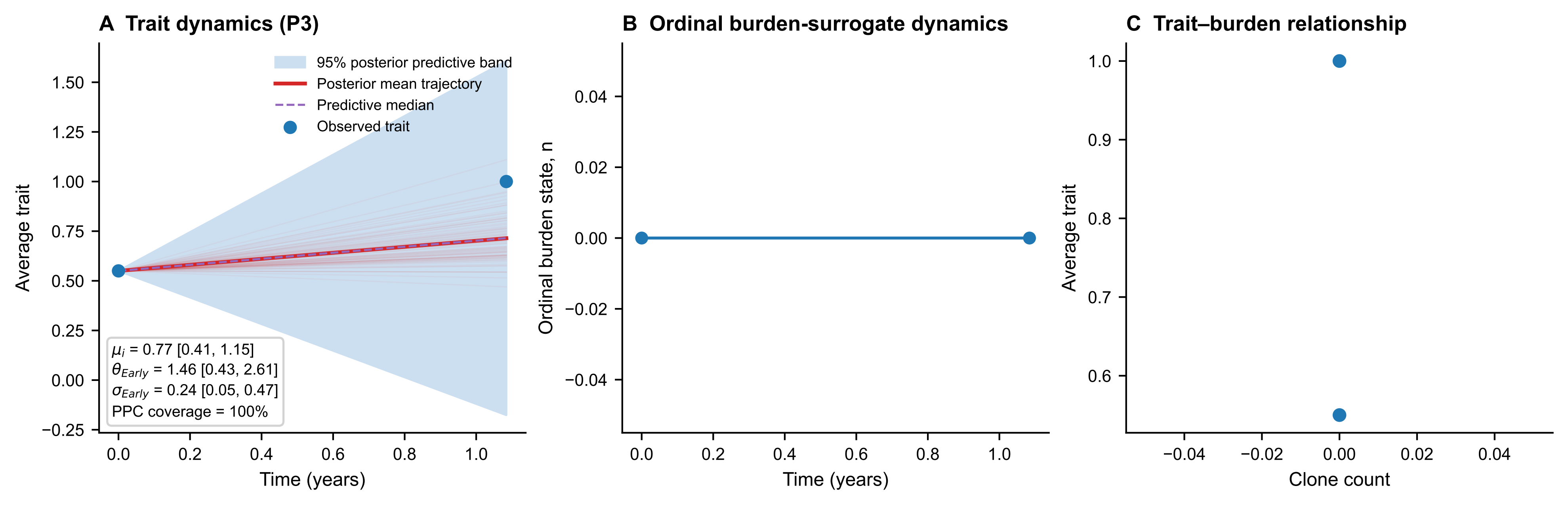

A Trait dynamics (P3)
95% posterior predictive band
Posterior mean trajectory
Predictive median
Observed trait
μ_i = 0.77 [0.41, 1.15]
θ_{Early} = 1.46 [0.43, 2.61]
σ_{Early} = 0.24 [0.05, 0.47]
PPC coverage = 100%
Average trait
Time (years)
B Ordinal burden-surrogate dynamics
Ordinal burden state, n
Time (years)
C Trait–burden relationship
Average trait
Clone count

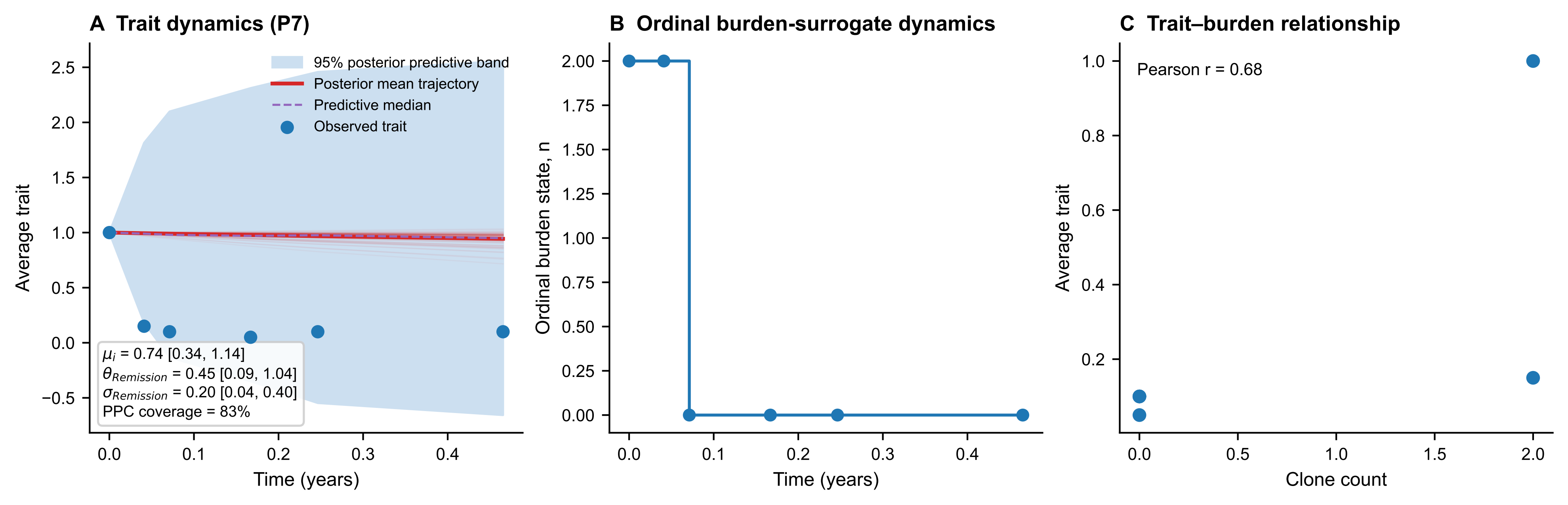

A Trait dynamics (P7)
95% posterior predictive band
Posterior mean trajectory
Predictive median
Observed trait
μ_i = 0.74 [0.34, 1.14]
$\theta_{Remission}$ = 0.45 [0.09, 1.04]
$\sigma_{Remission}$ = 0.20 [0.04, 0.40]
PPC coverage = 83%
Average trait
Time (years)
B Ordinal burden-surrogate dynamics
Ordinal burden state, n
Time (years)
C Trait–burden relationship
Pearson r = 0.68
Average trait
Clone count

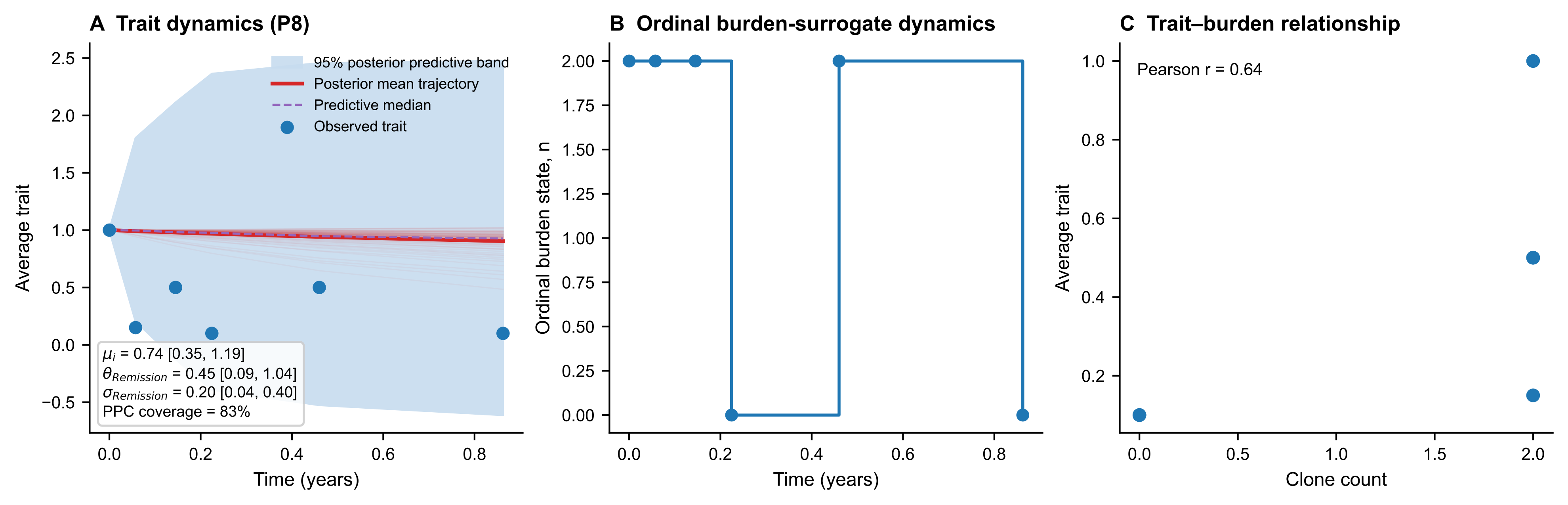

A Trait dynamics (P8)
95% posterior predictive band
Posterior mean trajectory
Predictive median
Observed trait
μ_i = 0.74 [0.35, 1.19]
$\theta_{Remission}$ = 0.45 [0.09, 1.04]
$\sigma_{Remission}$ = 0.20 [0.04, 0.40]
PPC coverage = 83%
Average trait
Time (years)
B Ordinal burden-surrogate dynamics
Ordinal burden state, n
Time (years)
C Trait–burden relationship
Pearson r = 0.64
Average trait
Clone count

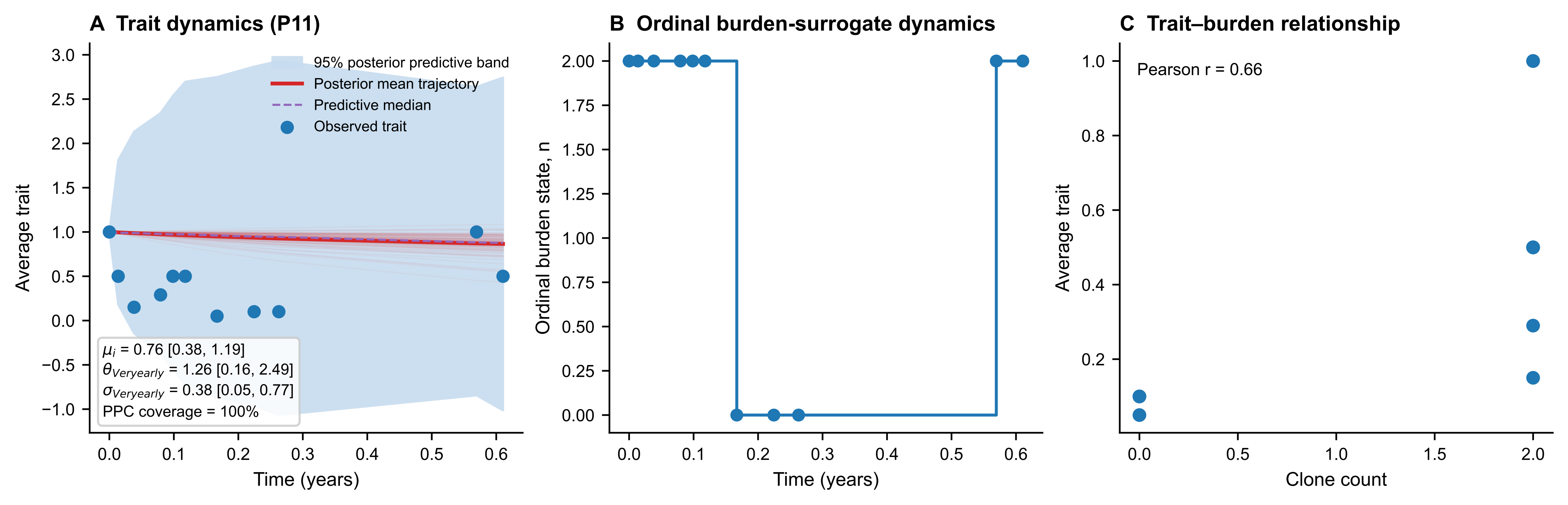

A Trait dynamics (P11)
95% posterior predictive band
Posterior mean trajectory
Predictive median
Observed trait
μ_i = 0.76 [0.38, 1.19]
$\theta_{Veryearly}$ = 1.26 [0.16, 2.49]
$\sigma_{Veryearly}$ = 0.38 [0.05, 0.77]
PPC coverage = 100%
Average trait
Time (years)
B Ordinal burden-surrogate dynamics
Ordinal burden state, n
Time (years)
C Trait–burden relationship
Pearson r = 0.66
Average trait
Clone count

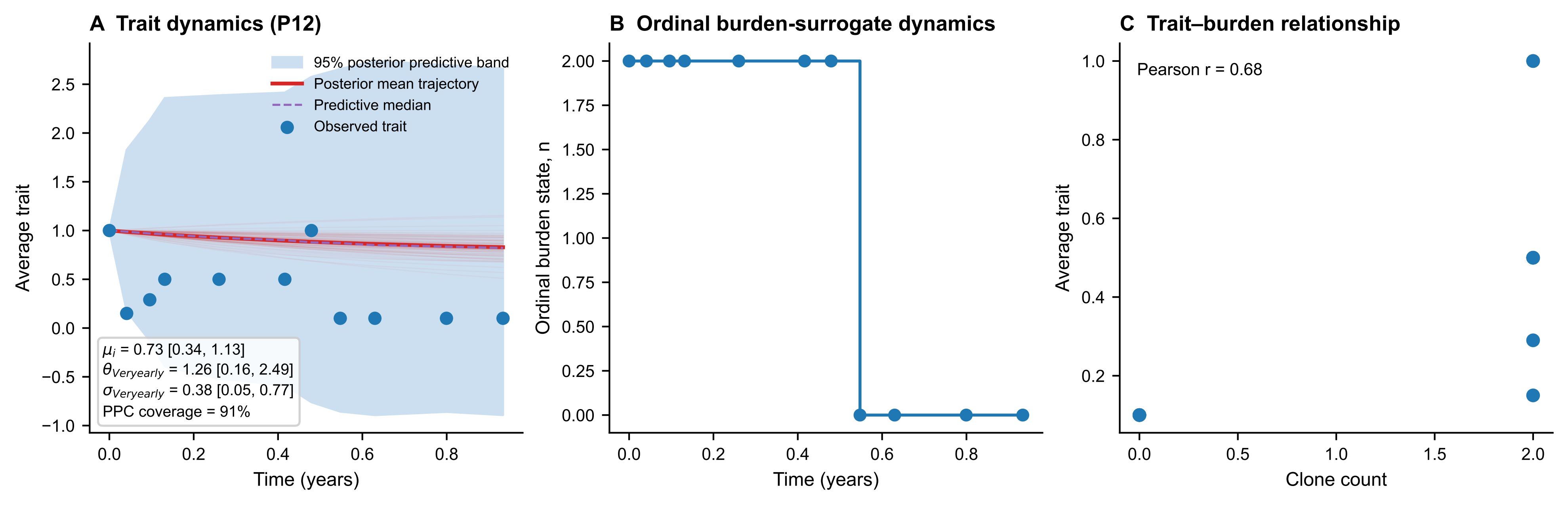

A Trait dynamics (P12)
95% posterior predictive band
Posterior mean trajectory
Predictive median
Observed trait
μ_i = 0.73 [0.34, 1.13]
$\theta_{Veryearly}$ = 1.26 [0.16, 2.49]
$\sigma_{Veryearly}$ = 0.38 [0.05, 0.77]
PPC coverage = 91%
Average trait
Time (years)
B Ordinal burden-surrogate dynamics
Ordinal burden state, n
Time (years)
C Trait–burden relationship
Pearson r = 0.68
Average trait
Clone count

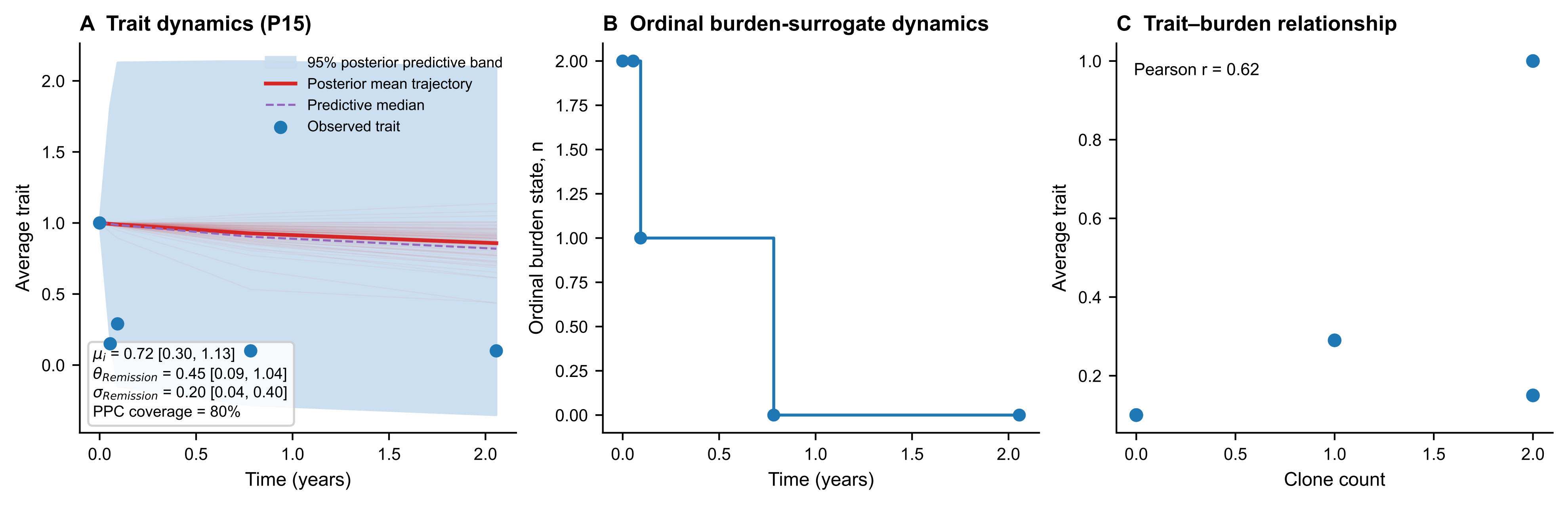
A Trait dynamics (P15)
95% posterior predictive band
Posterior mean trajectory
Predictive median
Observed trait
μ_i = 0.72 [0.30, 1.13]
$\theta_{Remission}$ = 0.45 [0.09, 1.04]
$\sigma_{Remission}$ = 0.20 [0.04, 0.40]
PPC coverage = 80%
Average trait
Time (years)
B Ordinal burden-surrogate dynamics
Ordinal burden state, n
Time (years)
C Trait–burden relationship
Pearson r = 0.62
Average trait
Clone count

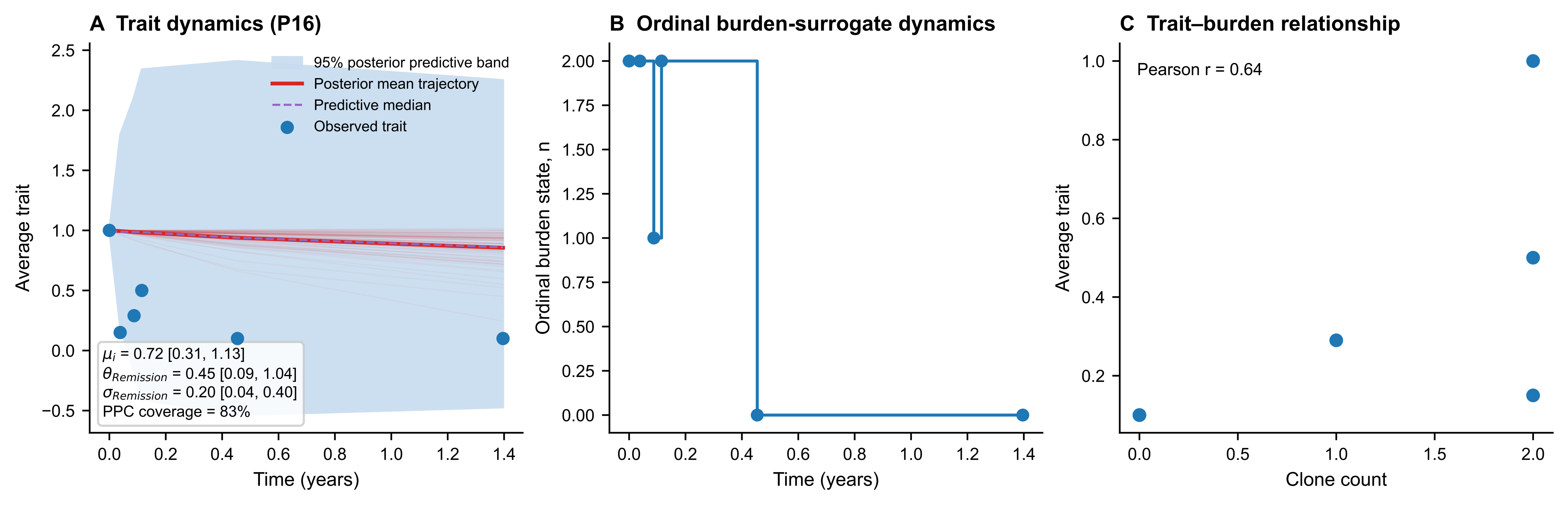

A Trait dynamics (P16)
95% posterior predictive band
Posterior mean trajectory
Predictive median
Observed trait
Average trait
Time (years)
μ_i = 0.72 [0.31, 1.13]
$\theta_{Remission}$ = 0.45 [0.09, 1.04]
$\sigma_{Remission}$ = 0.20 [0.04, 0.40]
PPC coverage = 83%
B Ordinal burden-surrogate dynamics
Ordinal burden state, n
Time (years)
C Trait–burden relationship
Pearson r = 0.64
Average trait
Clone count

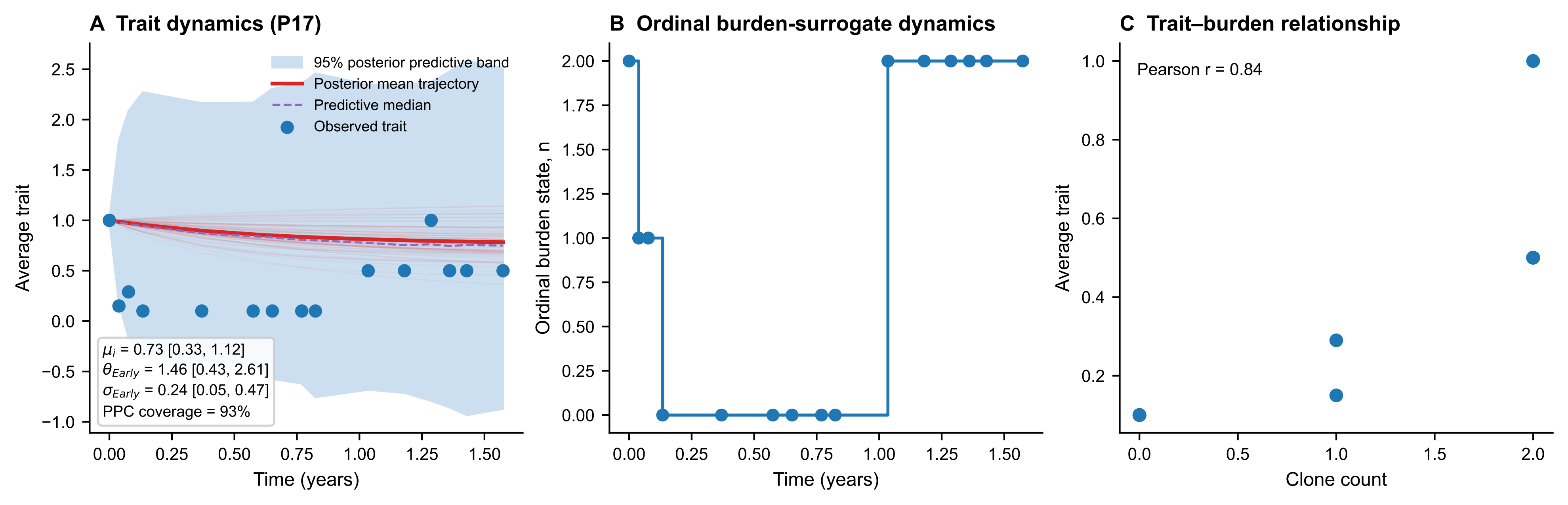
A Trait dynamics (P17)
95% posterior predictive band
Posterior mean trajectory
Predictive median
Observed trait
μ_i = 0.73 [0.33, 1.12]
θ_{Early} = 1.46 [0.43, 2.61]
σ_{Early} = 0.24 [0.05, 0.47]
PPC coverage = 93%
Average trait
Time (years)
B Ordinal burden-surrogate dynamics
Ordinal burden state, n
Time (years)
C Trait–burden relationship
Pearson r = 0.84
Average trait
Clone count

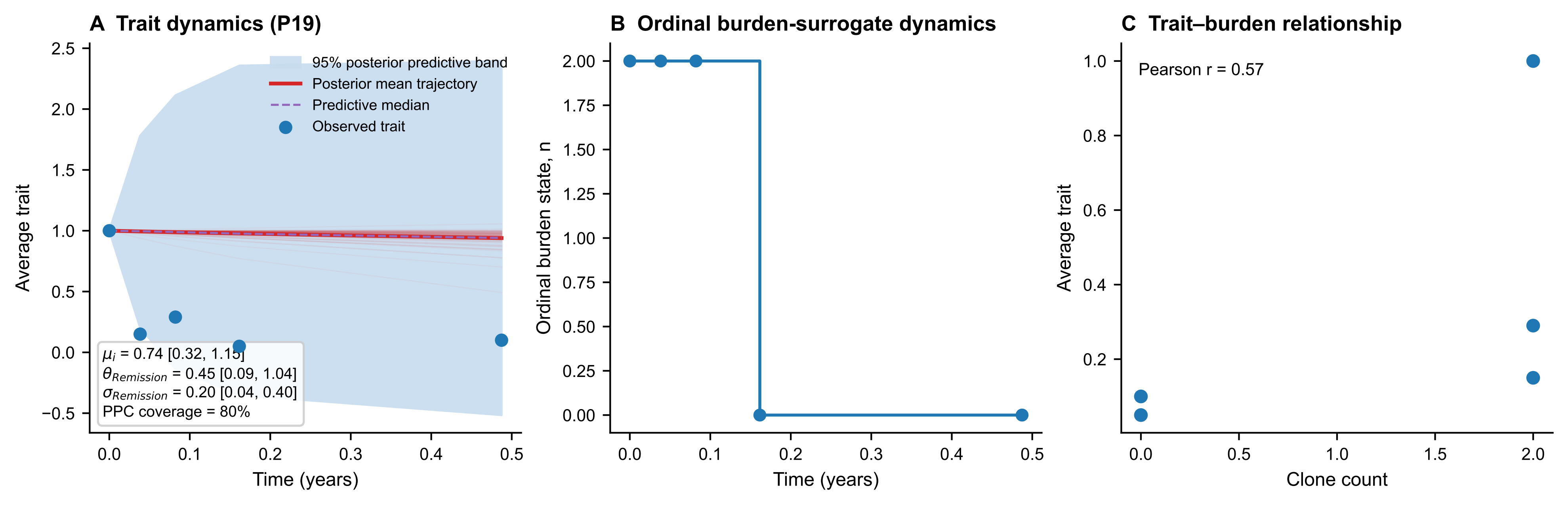
A Trait dynamics (P19)
95% posterior predictive band
Posterior mean trajectory
Predictive median
Observed trait
μ_i = 0.74 [0.32, 1.15]
$\theta_{Remission}$ = 0.45 [0.09, 1.04]
$\sigma_{Remission}$ = 0.20 [0.04, 0.40]
PPC coverage = 80%
Average trait
Time (years)
B Ordinal burden-surrogate dynamics
Ordinal burden state, n
Time (years)
C Trait–burden relationship
Pearson r = 0.57
Average trait
Clone count

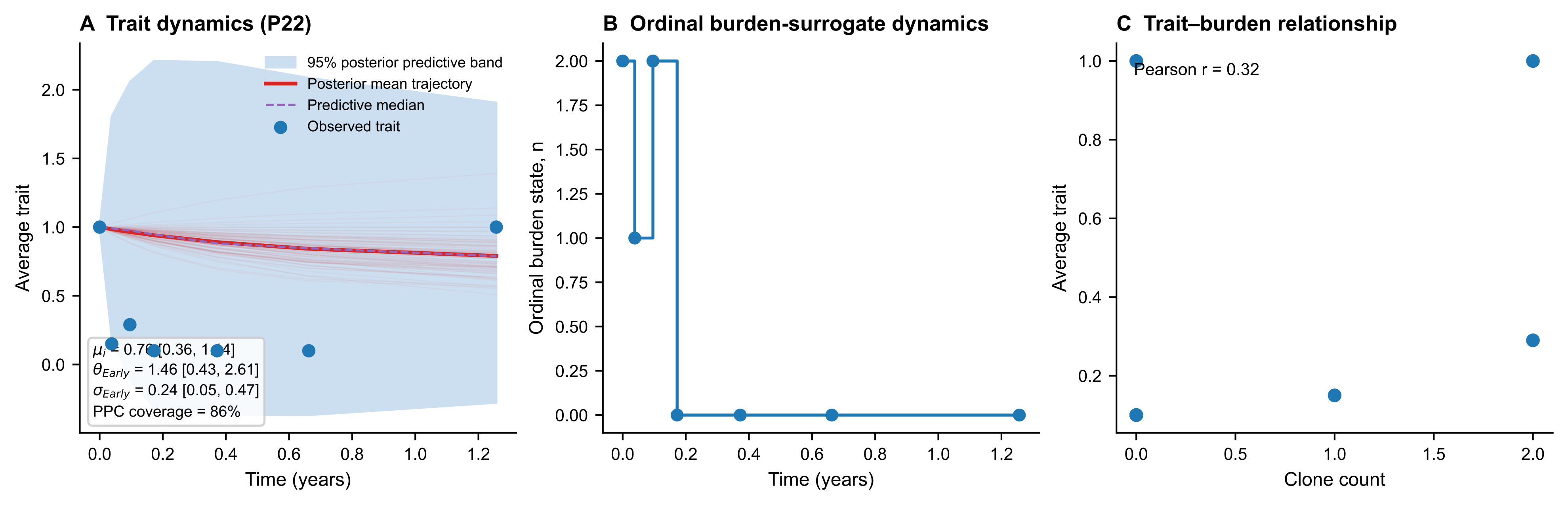

A Trait dynamics (P22)
95% posterior predictive band
Posterior mean trajectory
Predictive median
Observed trait
Average trait
Time (years)
μ_i = 0.76 [0.36, 1.14]
θ_{Early} = 1.46 [0.43, 2.61]
σ_{Early} = 0.24 [0.05, 0.47]
PPC coverage = 86%
B Ordinal burden-surrogate dynamics
Ordinal burden state, n
Time (years)
C Trait–burden relationship
Pearson r = 0.32
Average trait
Clone count

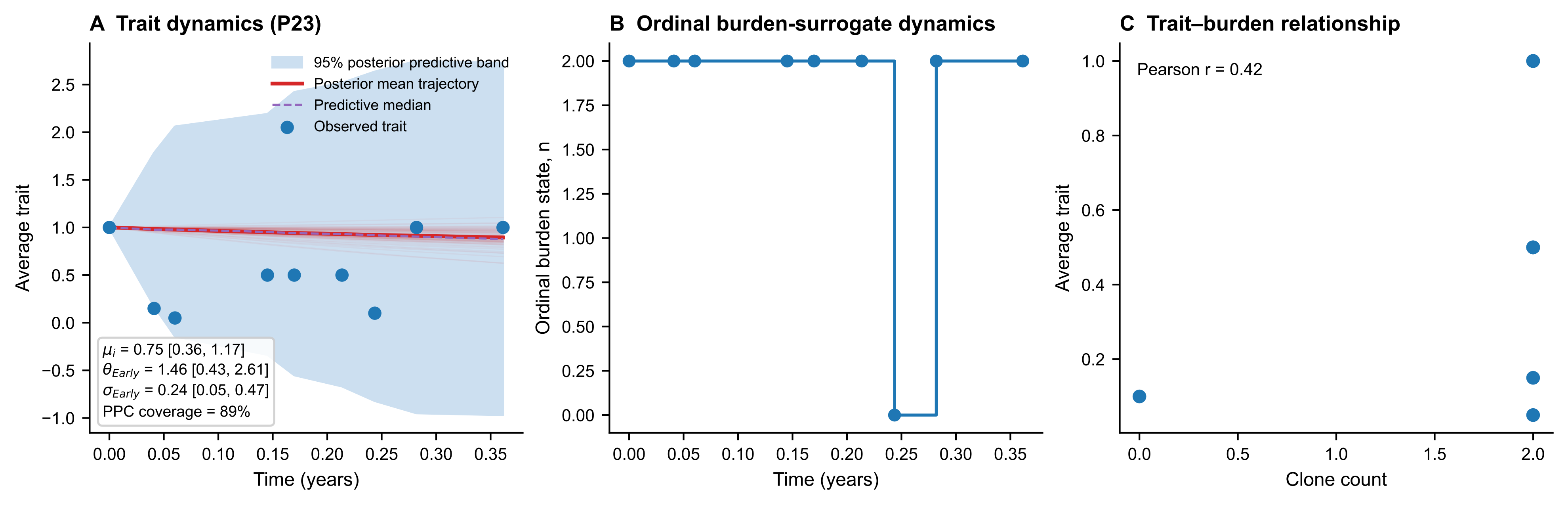

A Trait dynamics (P23)
95% posterior predictive band
Posterior mean trajectory
Predictive median
Observed trait
μ_i = 0.75 [0.36, 1.17]
θ_{Early} = 1.46 [0.43, 2.61]
σ_{Early} = 0.24 [0.05, 0.47]
PPC coverage = 89%
Average trait
Time (years)
B Ordinal burden-surrogate dynamics
Ordinal burden state, n
Time (years)
C Trait–burden relationship
Pearson r = 0.42
Average trait
Clone count

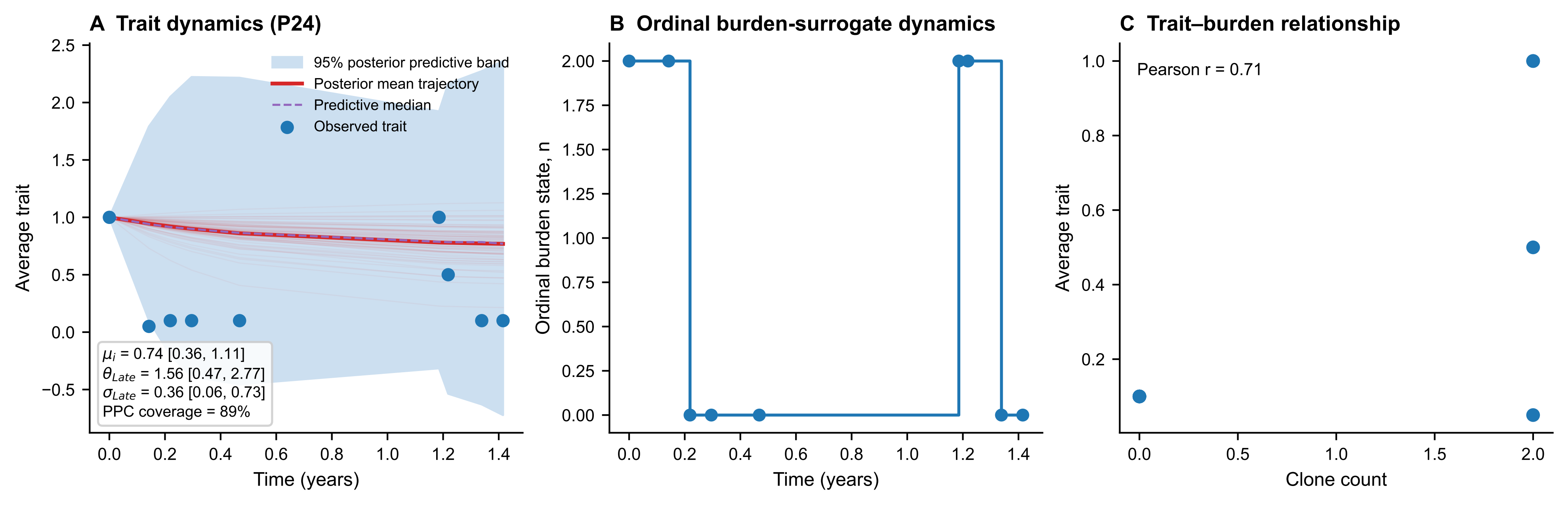

A Trait dynamics (P24)
95% posterior predictive band
Posterior mean trajectory
Predictive median
Observed trait
μ_i = 0.74 [0.36, 1.11]
θ_{Late} = 1.56 [0.47, 2.77]
σ_{Late} = 0.36 [0.06, 0.73]
PPC coverage = 89%
Average trait
Time (years)
B Ordinal burden-surrogate dynamics
Ordinal burden state, n
Time (years)
C Trait–burden relationship
Pearson r = 0.71
Average trait
Clone count

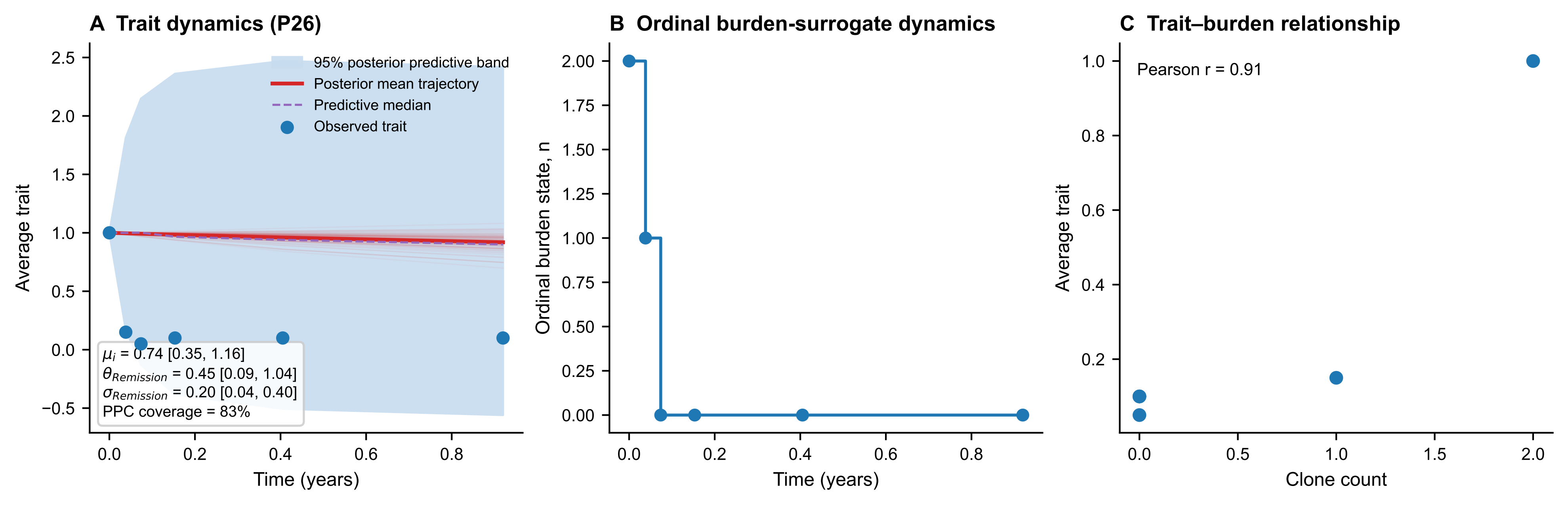
A Trait dynamics (P26)
95% posterior predictive band
Posterior mean trajectory
Predictive median
Observed trait
μ_i = 0.74 [0.35, 1.16]
$\theta_{Remission}$ = 0.45 [0.09, 1.04]
$\sigma_{Remission}$ = 0.20 [0.04, 0.40]
PPC coverage = 83%
Average trait
Time (years)
B Ordinal burden-surrogate dynamics
Ordinal burden state, n
Time (years)
C Trait–burden relationship
Pearson r = 0.91
Average trait
Clone count

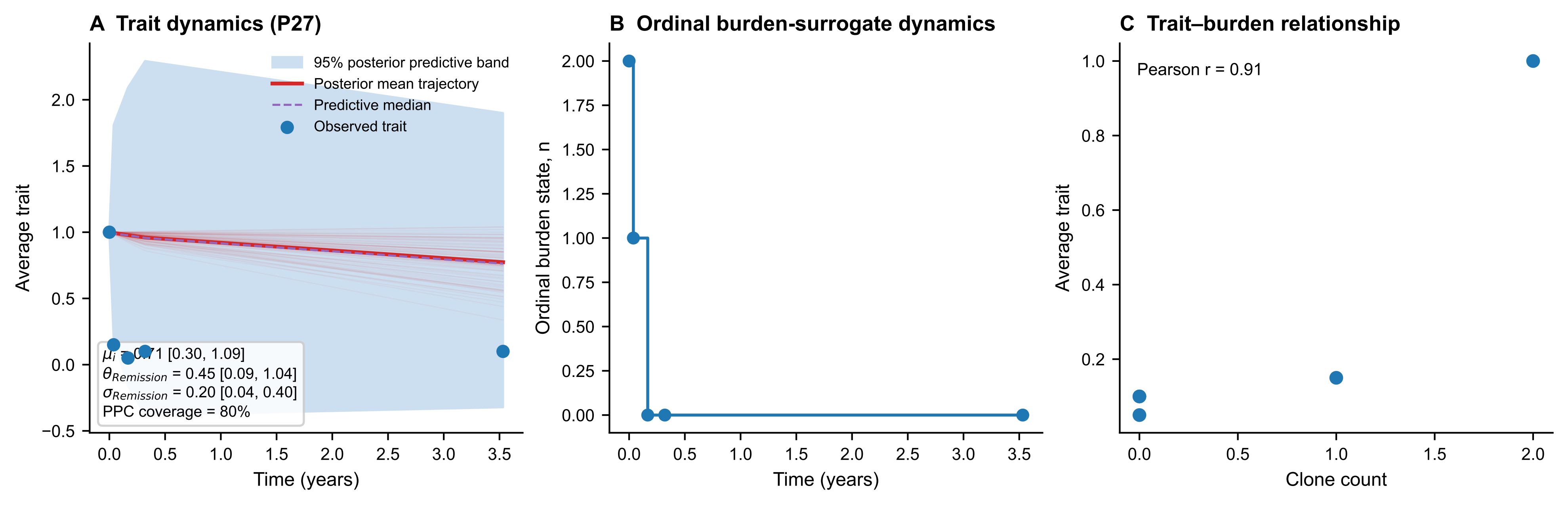

A Trait dynamics (P27)
95% posterior predictive band
Posterior mean trajectory
Predictive median
Observed trait
μ_i = 0.71 [0.30, 1.09]
$\theta_{Remission}$ = 0.45 [0.09, 1.04]
$\sigma_{Remission}$ = 0.20 [0.04, 0.40]
PPC coverage = 80%
Average trait
Time (years)
B Ordinal burden-surrogate dynamics
Ordinal burden state, n
Time (years)
C Trait–burden relationship
Pearson r = 0.91
Average trait
Clone count

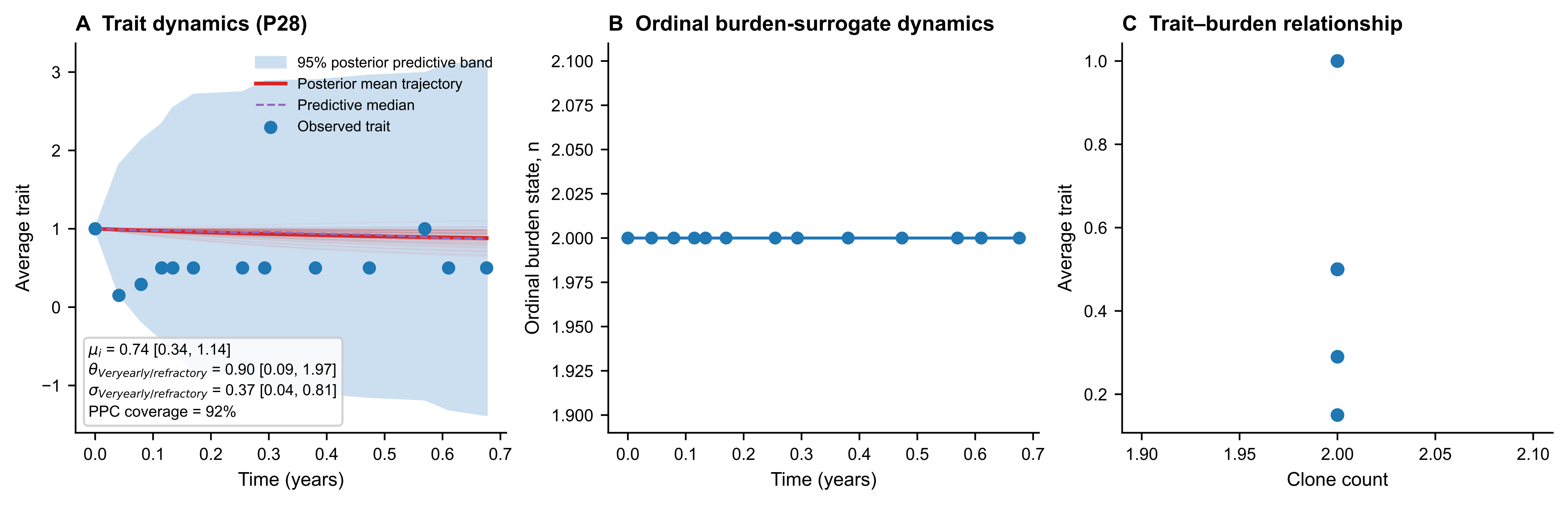

A Trait dynamics (P28)
95% posterior predictive band
Posterior mean trajectory
Predictive median
Observed trait
μ_i = 0.74 [0.34, 1.14]
$\theta_{Veryearly/refractory}$ = 0.90 [0.09, 1.97]
$\sigma_{Veryearly/refractory}$ = 0.37 [0.04, 0.81]
PPC coverage = 92%
Average trait
Time (years)
B Ordinal burden-surrogate dynamics
Ordinal burden state, n
Time (years)
C Trait–burden relationship
Average trait
Clone count

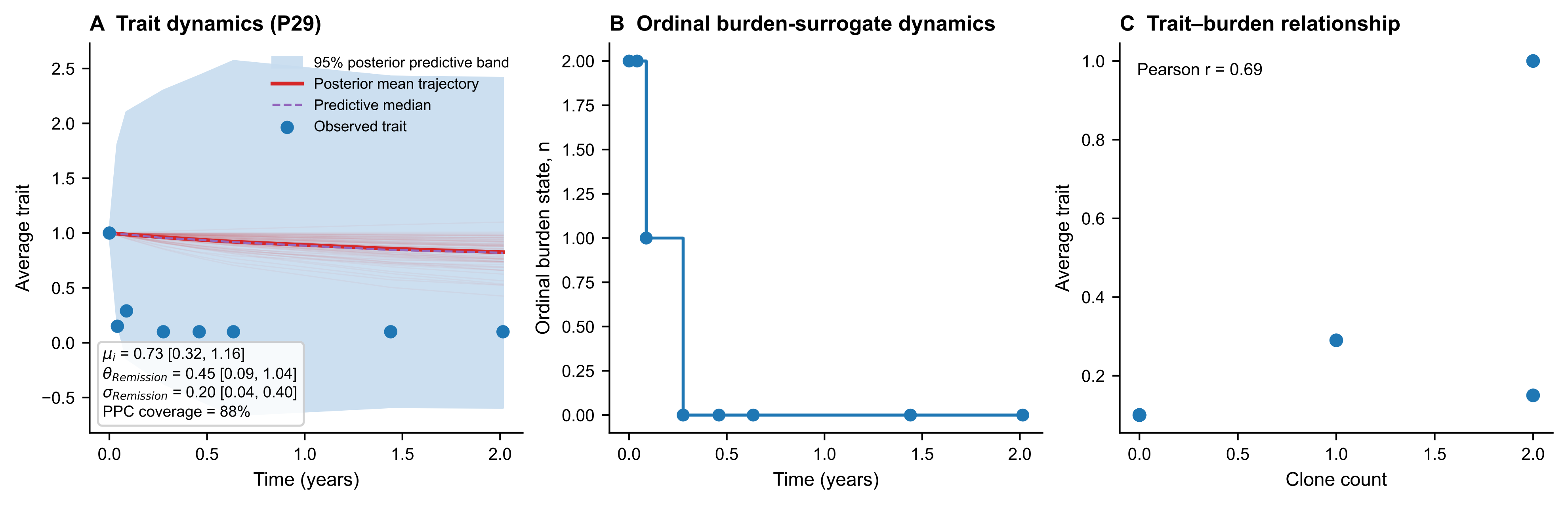
A Trait dynamics (P29)
95% posterior predictive band
Posterior mean trajectory
Predictive median
Observed trait
μ_i = 0.73 [0.32, 1.16]
$\theta_{Remission}$ = 0.45 [0.09, 1.04]
$\sigma_{Remission}$ = 0.20 [0.04, 0.40]
PPC coverage = 88%
Average trait
Time (years)
B Ordinal burden-surrogate dynamics
Ordinal burden state, n
Time (years)
C Trait–burden relationship
Pearson r = 0.69
Average trait
Clone count

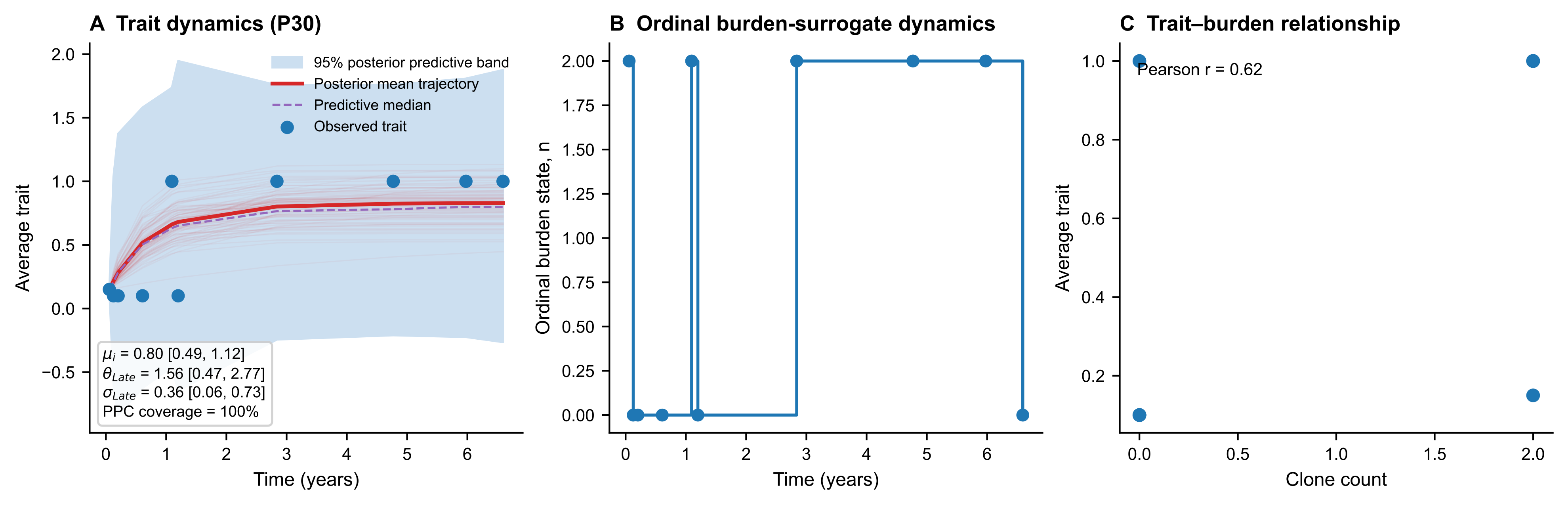
A Trait dynamics (P30)
95% posterior predictive band
Posterior mean trajectory
Predictive median
Observed trait
Average trait
Time (years)
μ_i = 0.80 [0.49, 1.12]
θ_{Late} = 1.56 [0.47, 2.77]
σ_{Late} = 0.36 [0.06, 0.73]
PPC coverage = 100%
B Ordinal burden-surrogate dynamics
Ordinal burden state, n
Time (years)
C Trait–burden relationship
Pearson r = 0.62
Average trait
Clone count

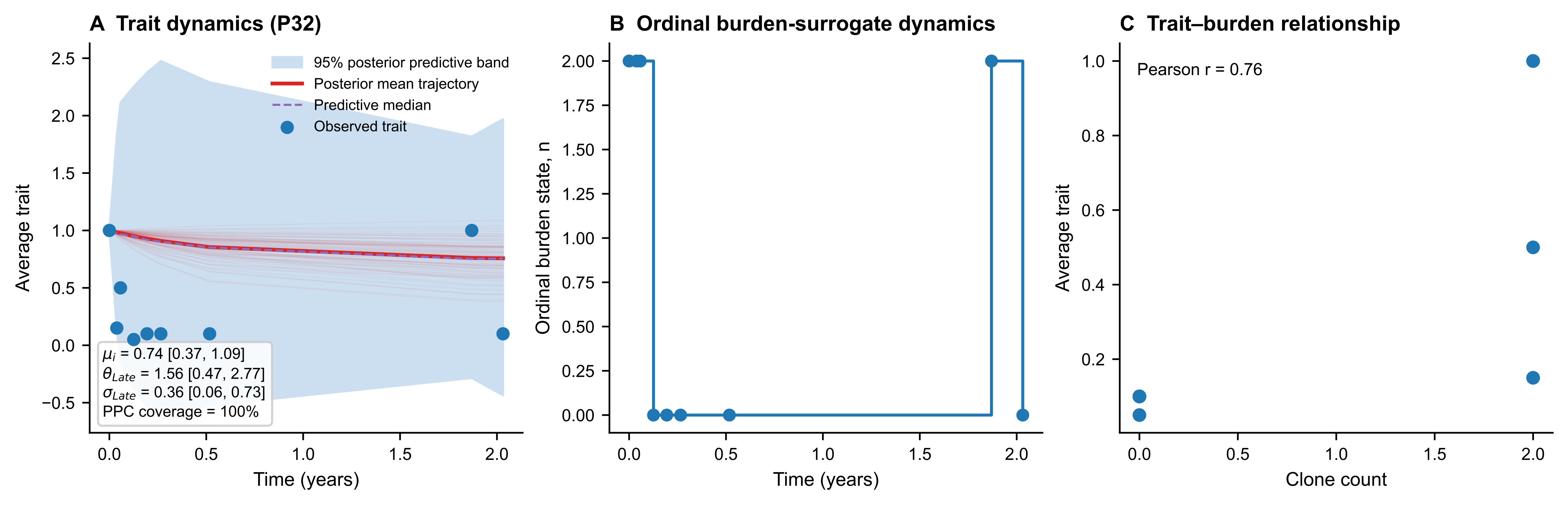

A Trait dynamics (P32)
95% posterior predictive band
Posterior mean trajectory
Predictive median
Observed trait
μ_i = 0.74 [0.37, 1.09]
θ_{Late} = 1.56 [0.47, 2.77]
σ_{Late} = 0.36 [0.06, 0.73]
PPC coverage = 100%
Average trait
Time (years)
B Ordinal burden-surrogate dynamics
Ordinal burden state, n
Time (years)
C Trait–burden relationship
Pearson r = 0.76
Average trait
Clone count

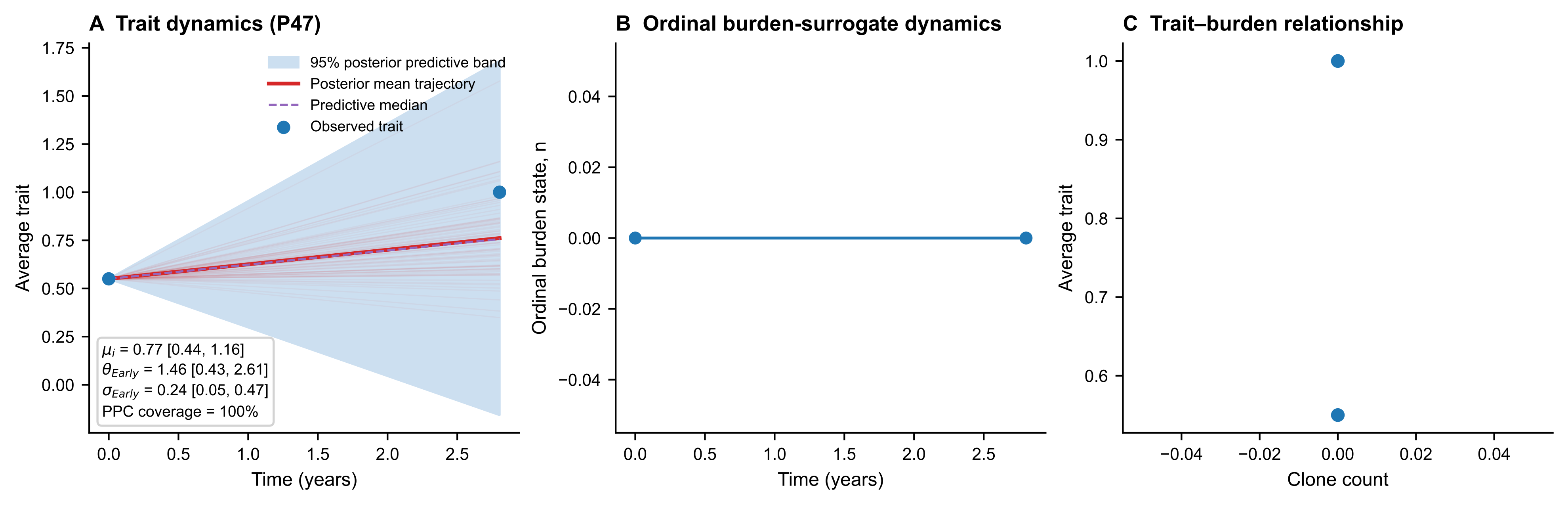

A Trait dynamics (P47)
95% posterior predictive band
Posterior mean trajectory
Predictive median
Observed trait
μ_i = 0.77 [0.44, 1.16]
θ_{Early} = 1.46 [0.43, 2.61]
σ_{Early} = 0.24 [0.05, 0.47]
PPC coverage = 100%
Average trait
Time (years)
B Ordinal burden-surrogate dynamics
Ordinal burden state, n
Time (years)
C Trait–burden relationship
Average trait
Clone count

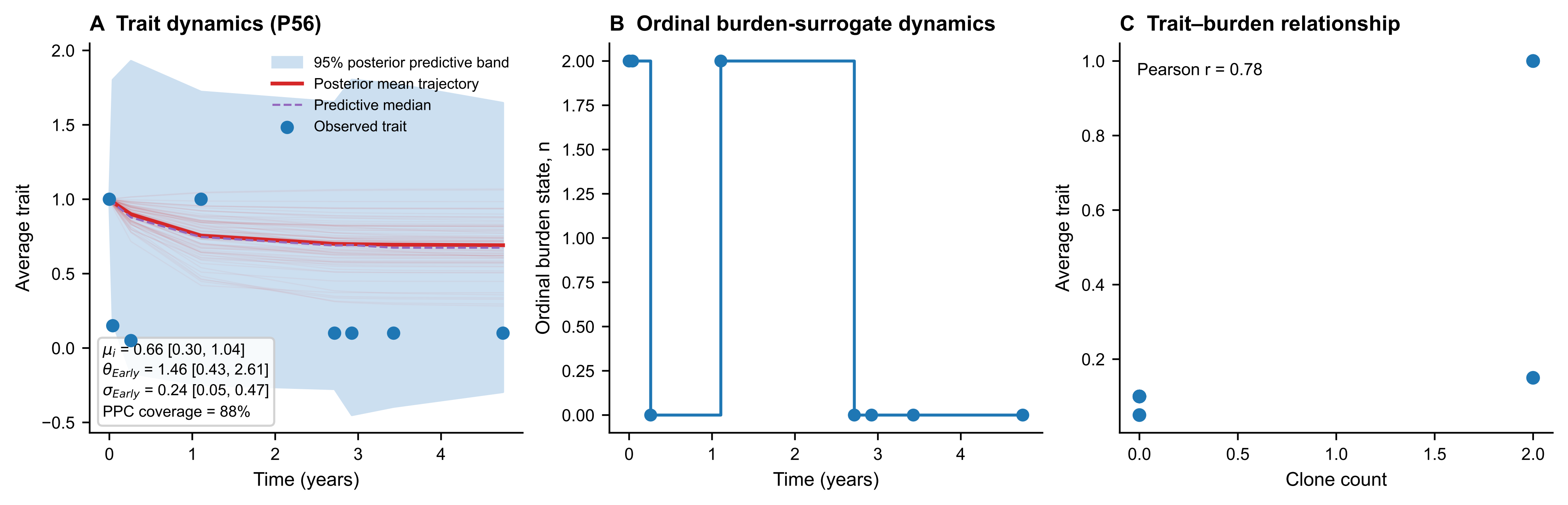
A Trait dynamics (P56)
95% posterior predictive band
Posterior mean trajectory
Predictive median
Observed trait
Average trait
Time (years)
μ_i = 0.66 [0.30, 1.04]
θ_{Early} = 1.46 [0.43, 2.61]
σ_{Early} = 0.24 [0.05, 0.47]
PPC coverage = 88%
B Ordinal burden-surrogate dynamics
Ordinal burden state, n
Time (years)
C Trait–burden relationship
Pearson r = 0.78
Average trait
Clone count

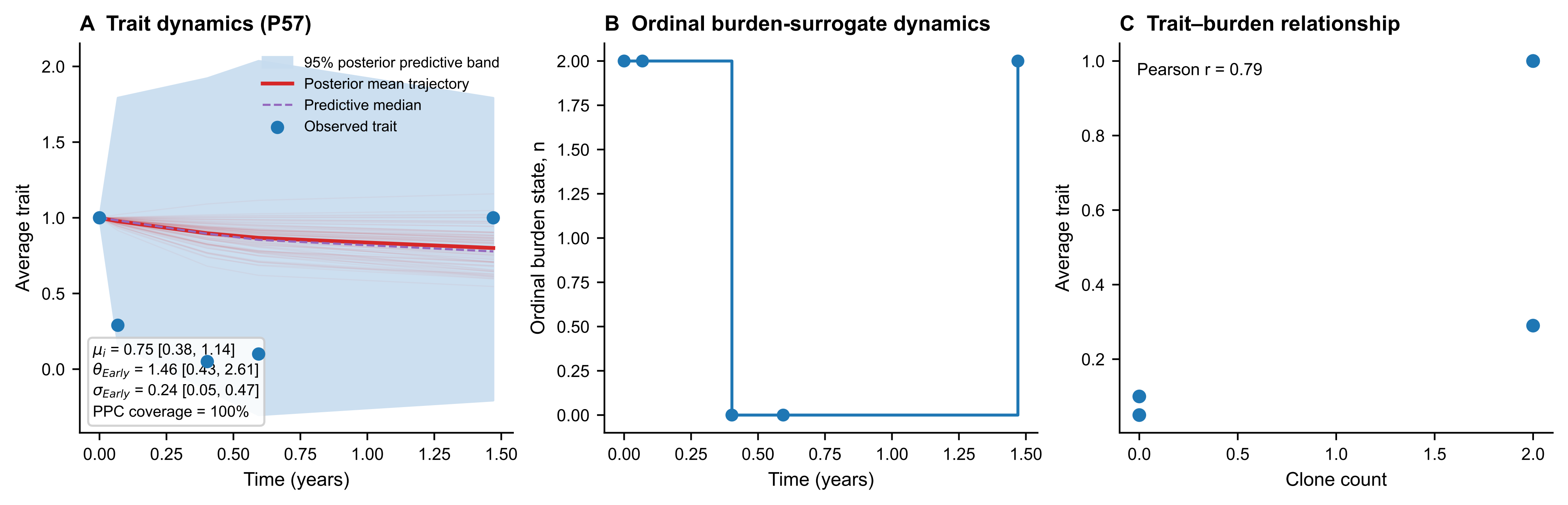

A Trait dynamics (P57)
95% posterior predictive band
Posterior mean trajectory
Predictive median
Observed trait
μ_i = 0.75 [0.38, 1.14]
θ_{Early} = 1.46 [0.43, 2.61]
σ_{Early} = 0.24 [0.05, 0.47]
PPC coverage = 100%
Average trait
Time (years)
B Ordinal burden-surrogate dynamics
Ordinal burden state, n
Time (years)
C Trait–burden relationship
Pearson r = 0.79
Average trait
Clone count

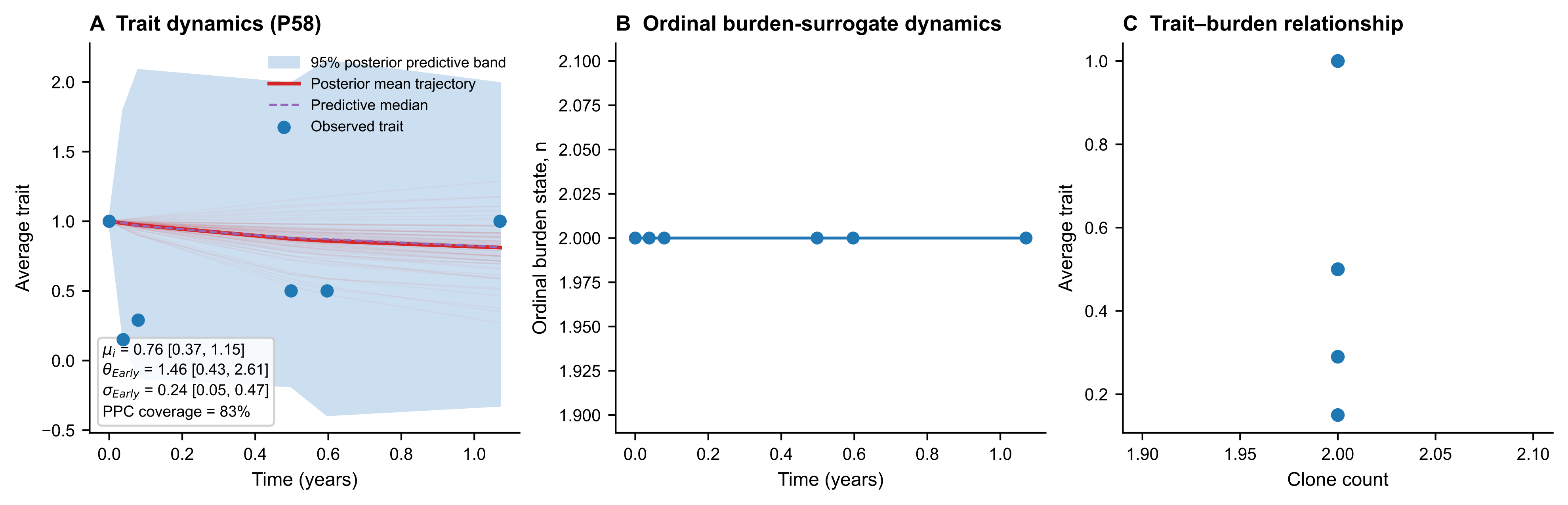

A Trait dynamics (P58)
95% posterior predictive band
Posterior mean trajectory
Predictive median
Observed trait
μ_i = 0.76 [0.37, 1.15]
θ_{Early} = 1.46 [0.43, 2.61]
σ_{Early} = 0.24 [0.05, 0.47]
PPC coverage = 83%
Average trait
Time (years)
B Ordinal burden-surrogate dynamics
Ordinal burden state, n
Time (years)
C Trait–burden relationship
Average trait
Clone count

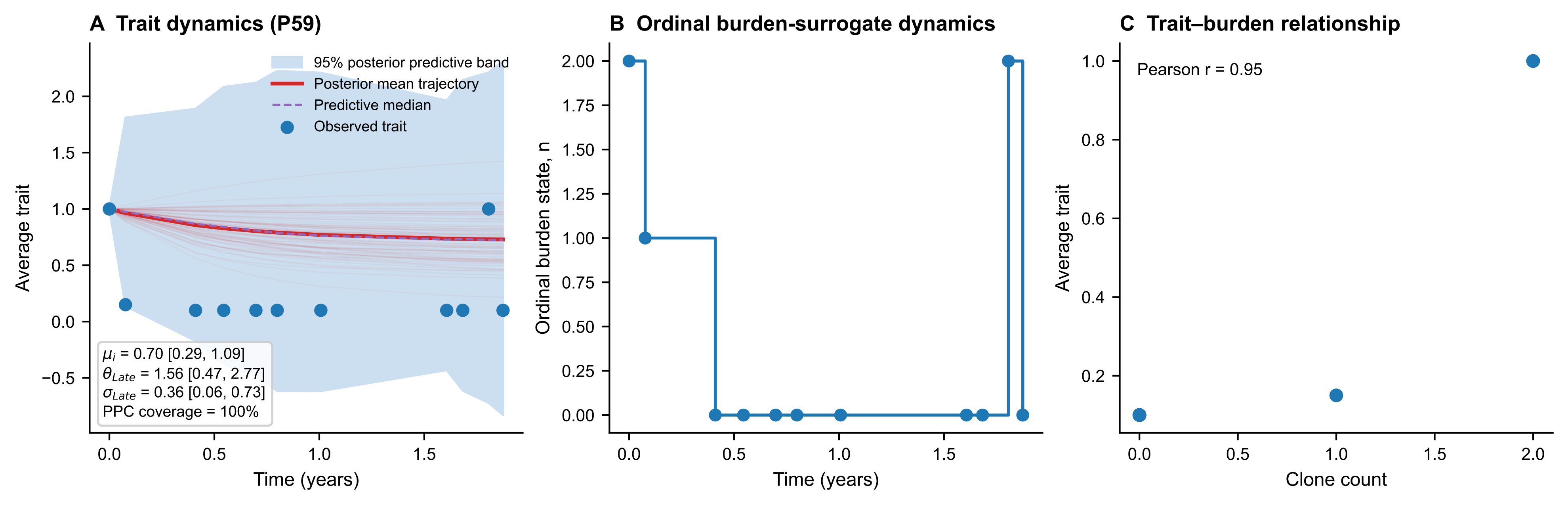
A Trait dynamics (P59)
95% posterior predictive band
Posterior mean trajectory
Predictive median
Observed trait
μ_i = 0.70 [0.29, 1.09]
θ_{Late} = 1.56 [0.47, 2.77]
σ_{Late} = 0.36 [0.06, 0.73]
PPC coverage = 100%
Average trait
Time (years)
B Ordinal burden-surrogate dynamics
Ordinal burden state, n
Time (years)
C Trait–burden relationship
Pearson r = 0.95
Average trait
Clone count

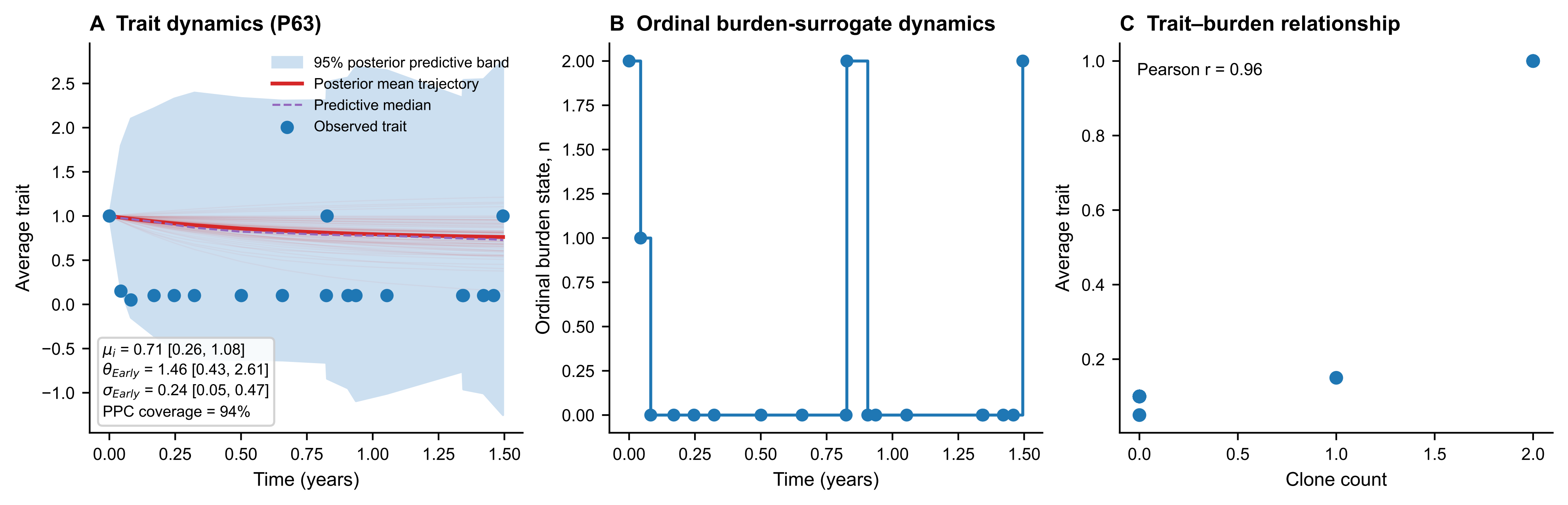

A Trait dynamics (P63)
95% posterior predictive band
Posterior mean trajectory
Predictive median
Observed trait
μ_i = 0.71 [0.26, 1.08]
θ_{Early} = 1.46 [0.43, 2.61]
σ_{Early} = 0.24 [0.05, 0.47]
PPC coverage = 94%
Average trait
Time (years)
B Ordinal burden-surrogate dynamics
Ordinal burden state, n
Time (years)
C Trait–burden relationship
Pearson r = 0.96
Average trait
Clone count

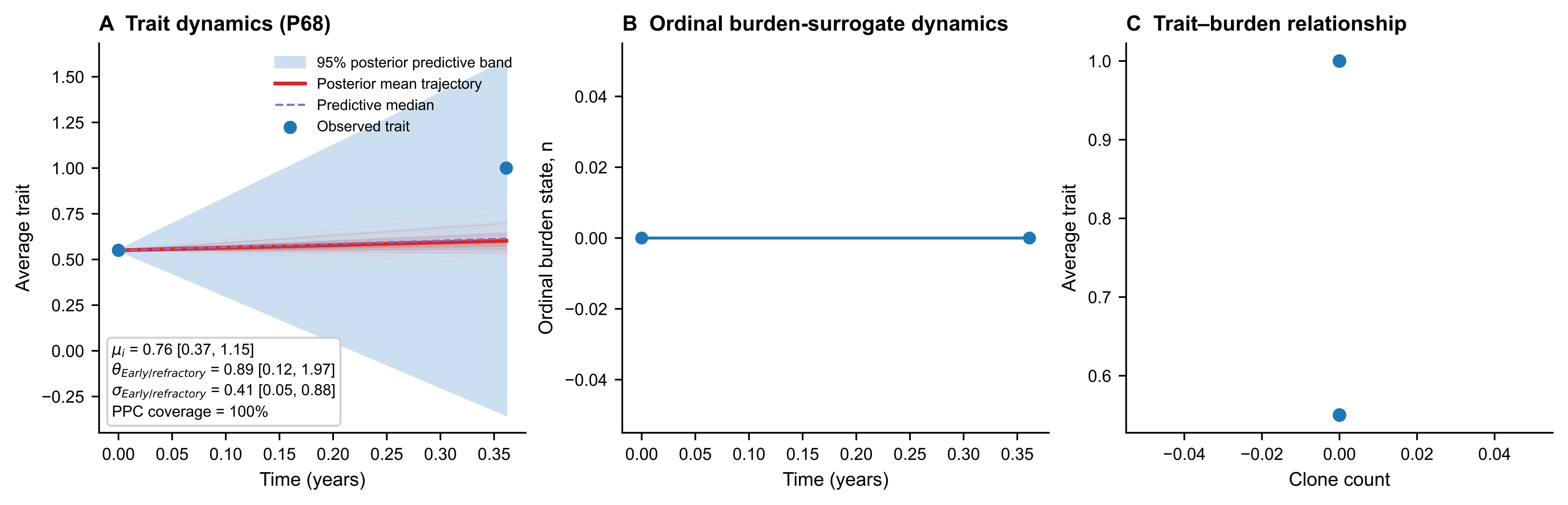
A Trait dynamics (P68)
B Ordinal burden-surrogate dynamics
C Trait–burden relationship
95% posterior predictive band
Posterior mean trajectory
Predictive median
Observed trait
μ_i = 0.76 [0.37, 1.15]
$\theta_{Early/refractory}$ = 0.89 [0.12, 1.97]
$\sigma_{Early/refractory}$ = 0.41 [0.05, 0.88]
PPC coverage = 100%
Average trait
Time (years)
Ordinal burden state, n
Clone count

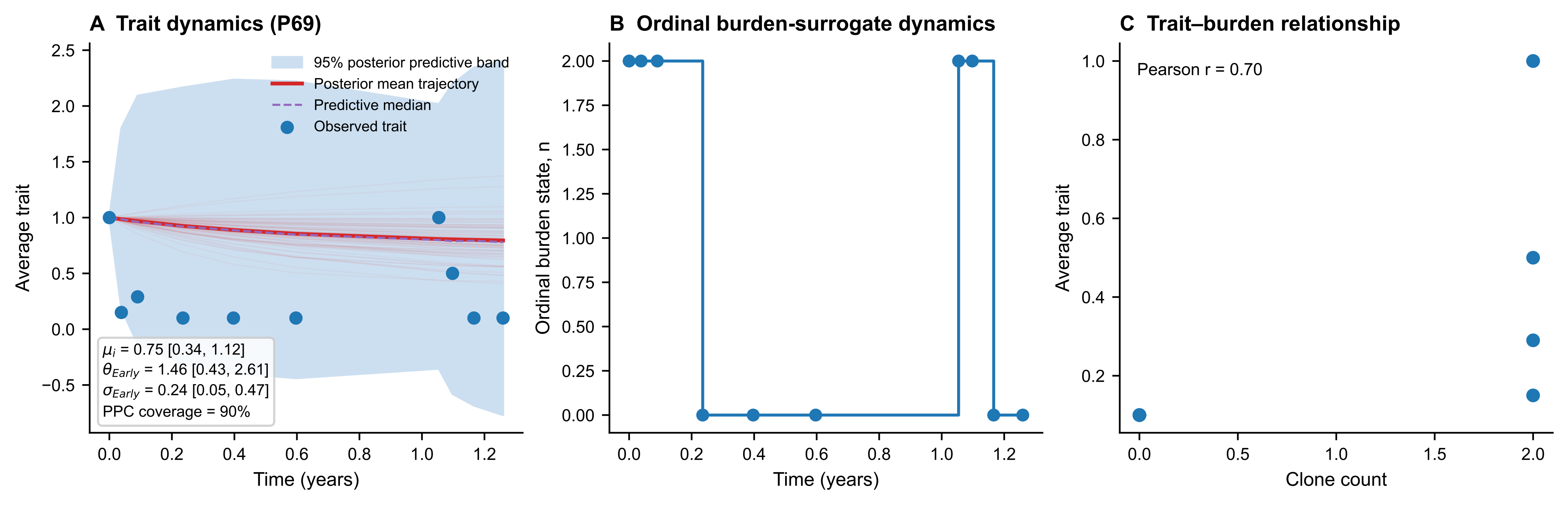

A Trait dynamics (P69)
95% posterior predictive band
Posterior mean trajectory
Predictive median
Observed trait
μ_i = 0.75 [0.34, 1.12]
θ_{Early} = 1.46 [0.43, 2.61]
σ_{Early} = 0.24 [0.05, 0.47]
PPC coverage = 90%
Average trait
Time (years)
B Ordinal burden-surrogate dynamics
Ordinal burden state, n
Time (years)
C Trait–burden relationship
Pearson r = 0.70
Average trait
Clone count

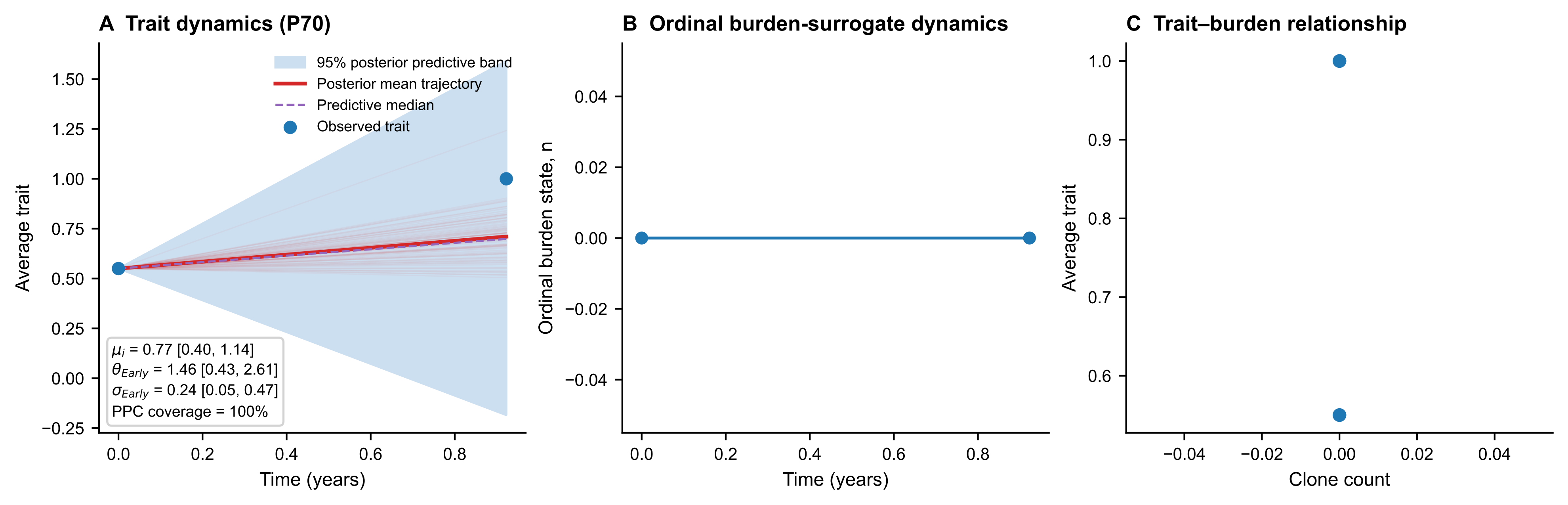

A Trait dynamics (P70)
95% posterior predictive band
Posterior mean trajectory
Predictive median
Observed trait
Average trait
Time (years)
μi = 0.77 [0.40, 1.14]
θEarly = 1.46 [0.43, 2.61]
σEarly = 0.24 [0.05, 0.47]
PPC coverage = 100%
B Ordinal burden-surrogate dynamics
Ordinal burden state, n
Time (years)
C Trait–burden relationship
Average trait
Clone count

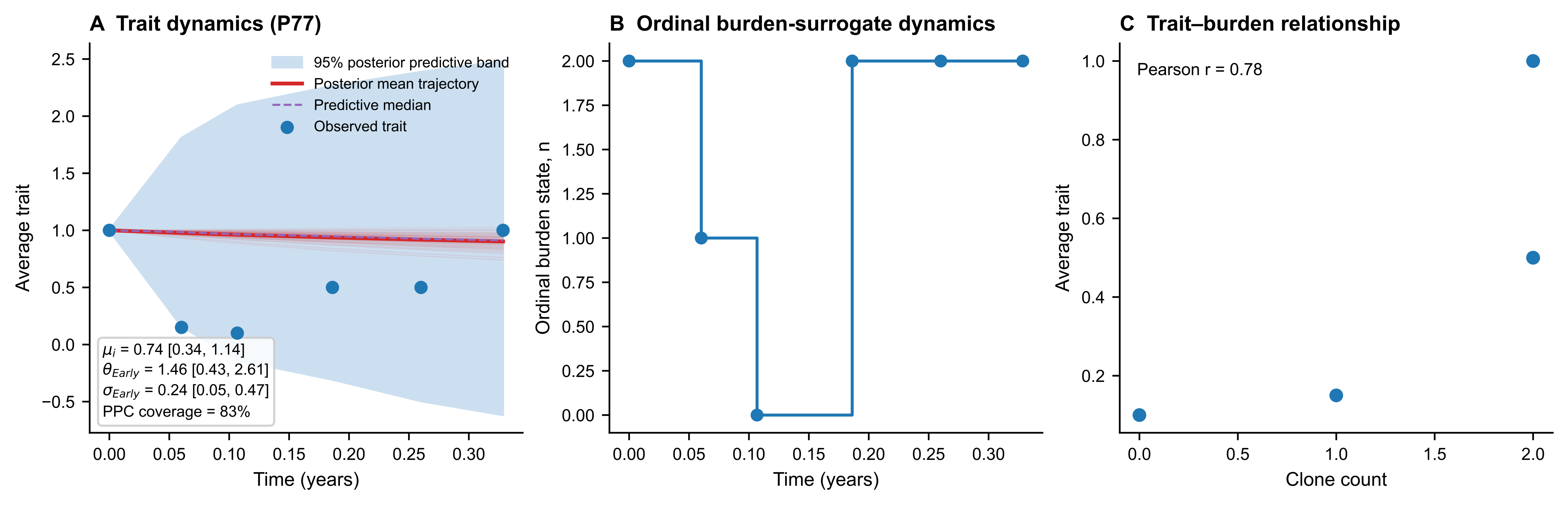
A Trait dynamics (P77)
95% posterior predictive band
Posterior mean trajectory
Predictive median
Observed trait
μ_i = 0.74 [0.34, 1.14]
θ_{Early} = 1.46 [0.43, 2.61]
σ_{Early} = 0.24 [0.05, 0.47]
PPC coverage = 83%
Average trait
Time (years)
B Ordinal burden-surrogate dynamics
Ordinal burden state, n
Time (years)
C Trait–burden relationship
Pearson r = 0.78
Average trait
Clone count

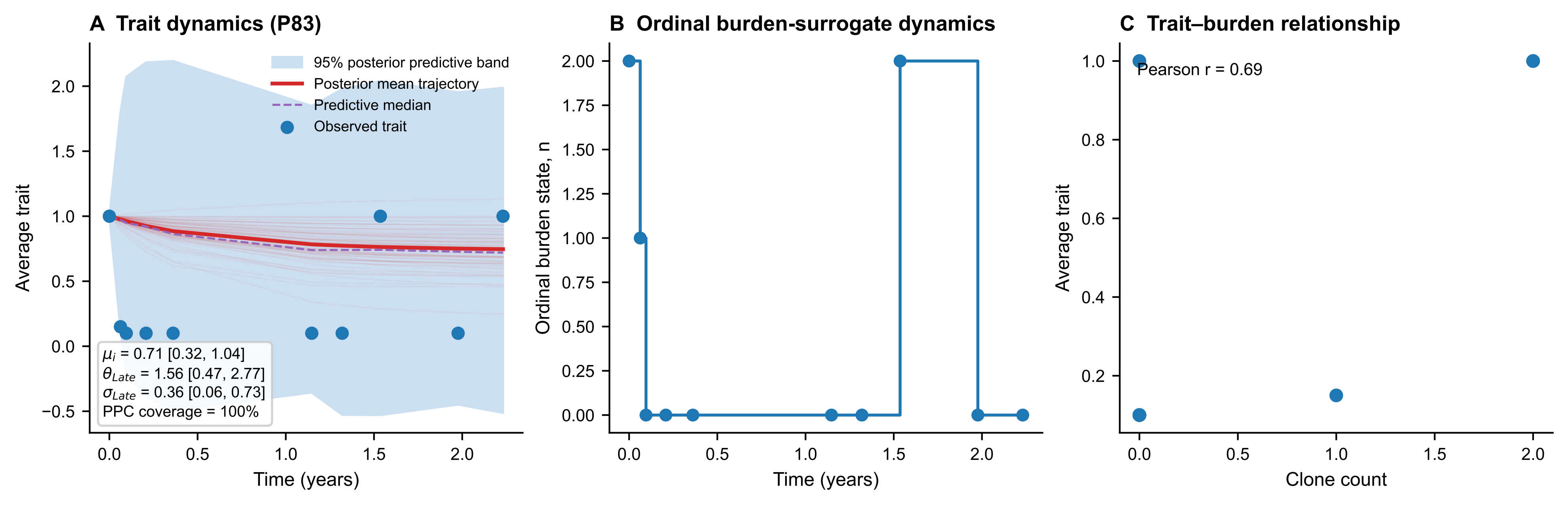

A Trait dynamics (P83)
95% posterior predictive band
Posterior mean trajectory
Predictive median
Observed trait
μ_i = 0.71 [0.32, 1.04]
θ_{Late} = 1.56 [0.47, 2.77]
σ_{Late} = 0.36 [0.06, 0.73]
PPC coverage = 100%
Average trait
Time (years)
B Ordinal burden-surrogate dynamics
Ordinal burden state, n
Time (years)
C Trait–burden relationship
Pearson r = 0.69
Average trait
Clone count

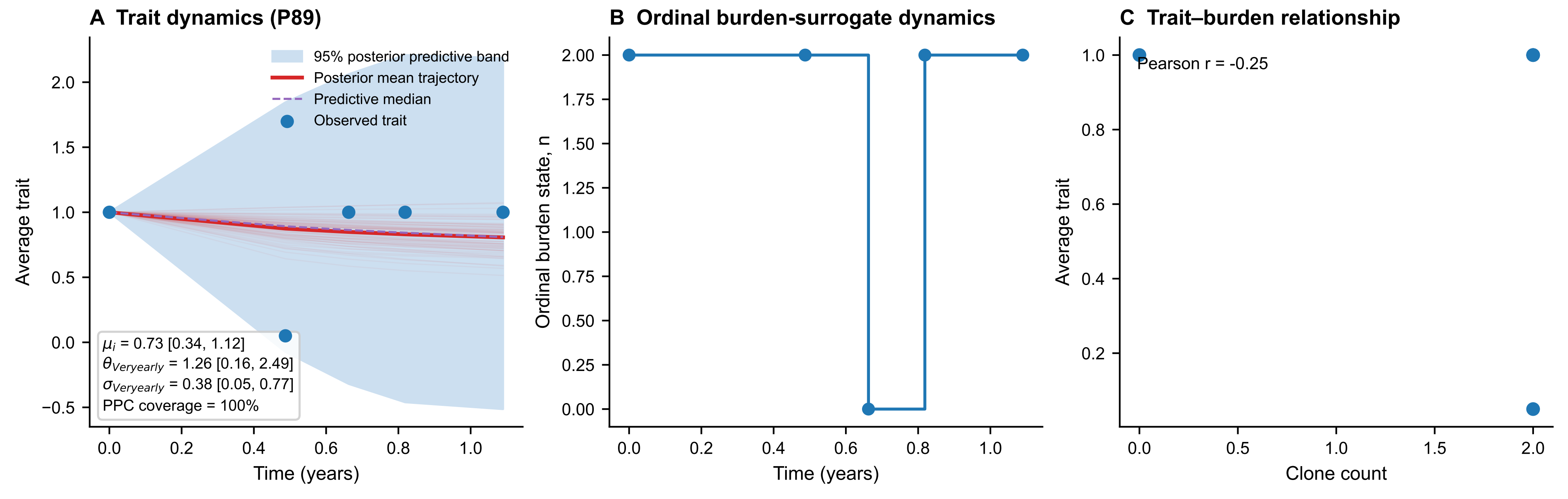

A Trait dynamics (P89)
95% posterior predictive band
Posterior mean trajectory
Predictive median
Observed trait
Average trait
Time (years)
μ_i = 0.73 [0.34, 1.12]
$\theta_{Veryearly}$ = 1.26 [0.16, 2.49]
$\sigma_{Veryearly}$ = 0.38 [0.05, 0.77]
PPC coverage = 100%
B Ordinal burden-surrogate dynamics
Ordinal burden state, n
Time (years)
C Trait–burden relationship
Pearson r = -0.25
Average trait
Clone count

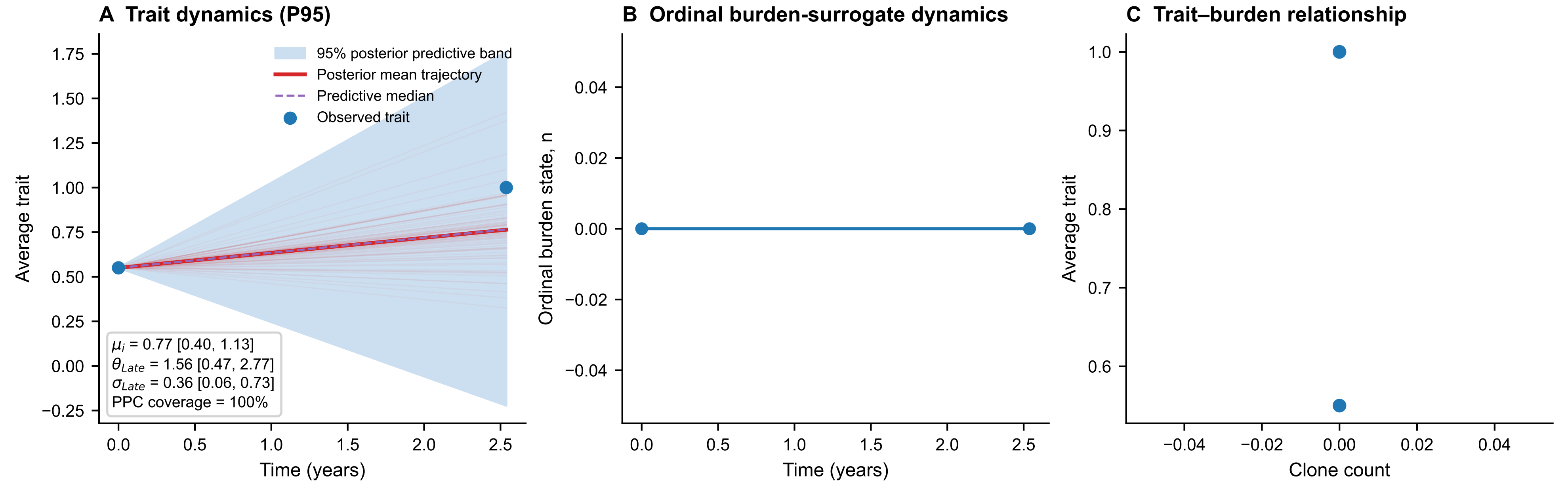
A Trait dynamics (P95)
95% posterior predictive band
Posterior mean trajectory
Predictive median
Observed trait
μ_i = 0.77 [0.40, 1.13]
θ_{Late} = 1.56 [0.47, 2.77]
σ_{Late} = 0.36 [0.06, 0.73]
PPC coverage = 100%
Average trait
Time (years)
B Ordinal burden-surrogate dynamics
Ordinal burden state, n
Time (years)
C Trait–burden relationship
Average trait
Clone count

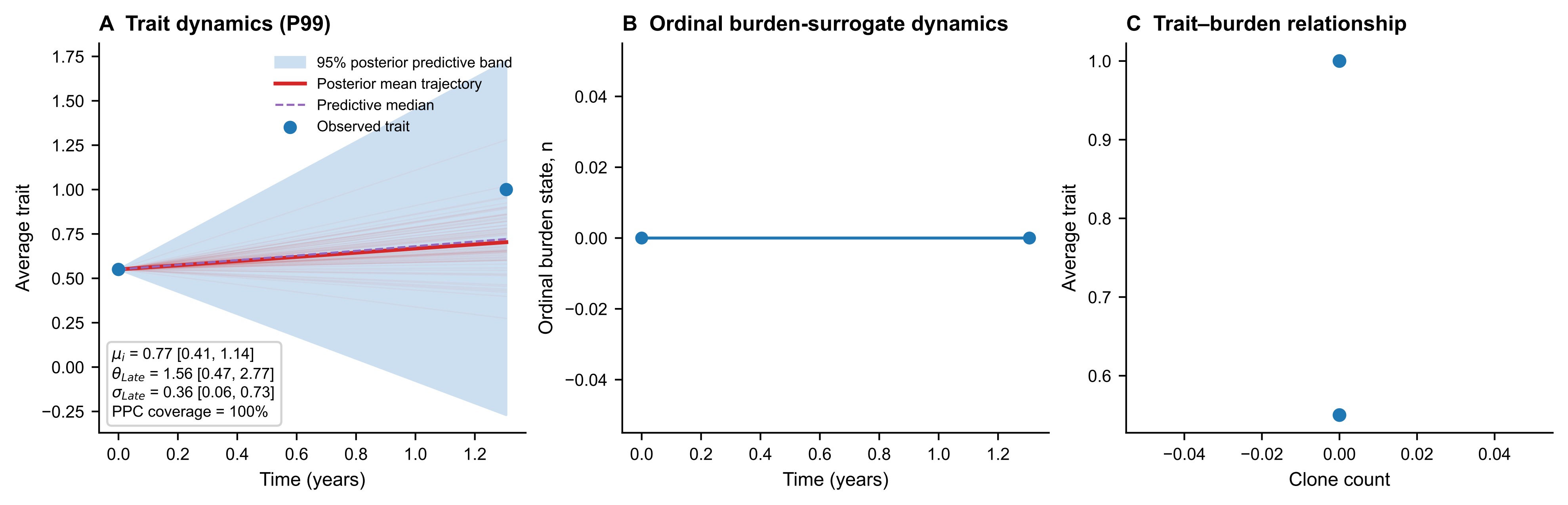

A Trait dynamics (P99)
B Ordinal burden-surrogate dynamics
C Trait–burden relationship
95% posterior predictive band
Posterior mean trajectory
Predictive median
Observed trait
μ_i = 0.77 [0.41, 1.14]
θ_{Late} = 1.56 [0.47, 2.77]
σ_{Late} = 0.36 [0.06, 0.73]
PPC coverage = 100%
Average trait
Time (years)
Ordinal burden state, n
Time (years)
Average trait
Clone count

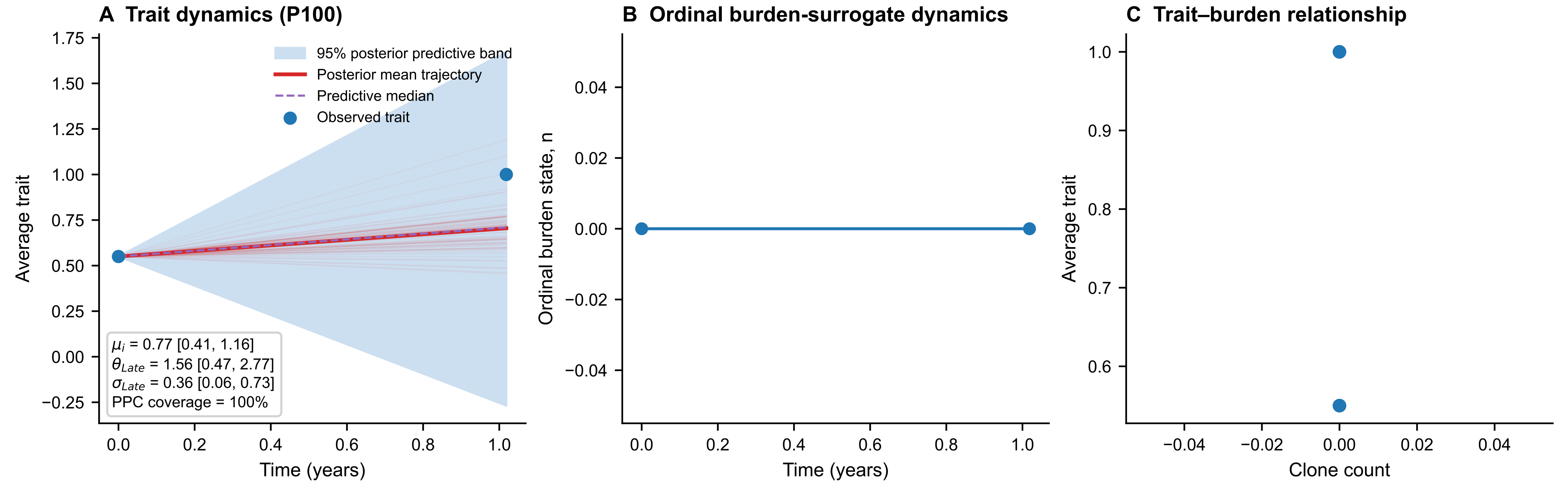

A Trait dynamics (P100)
95% posterior predictive band
Posterior mean trajectory
Predictive median
Observed trait
μ_i = 0.77 [0.41, 1.16]
θ_{Late} = 1.56 [0.47, 2.77]
σ_{Late} = 0.36 [0.06, 0.73]
PPC coverage = 100%
Average trait
Time (years)
B Ordinal burden-surrogate dynamics
Ordinal burden state, n
Time (years)
C Trait–burden relationship
Average trait
Clone count

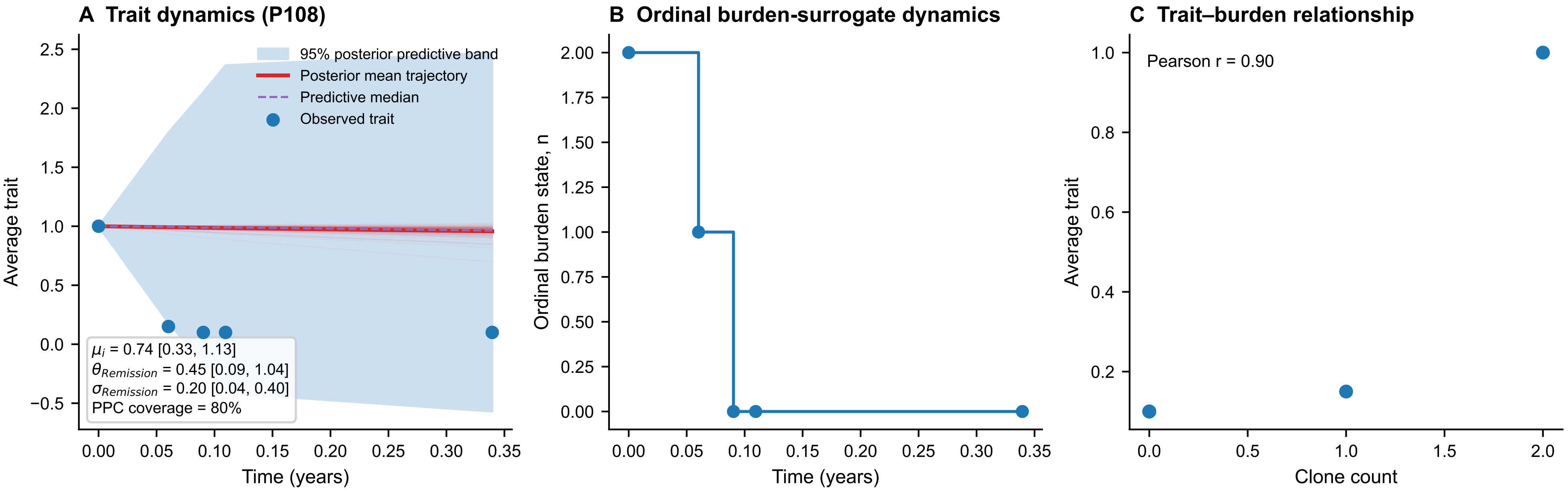

A Trait dynamics (P108)
95% posterior predictive band
Posterior mean trajectory
Predictive median
Observed trait
μ_i = 0.74 [0.33, 1.13]
$\theta_{Remission}$ = 0.45 [0.09, 1.04]
$\sigma_{Remission}$ = 0.20 [0.04, 0.40]
PPC coverage = 80%
Average trait
Time (years)
B Ordinal burden-surrogate dynamics
Ordinal burden state, n
Time (years)
C Trait–burden relationship
Pearson r = 0.90
Average trait
Clone count

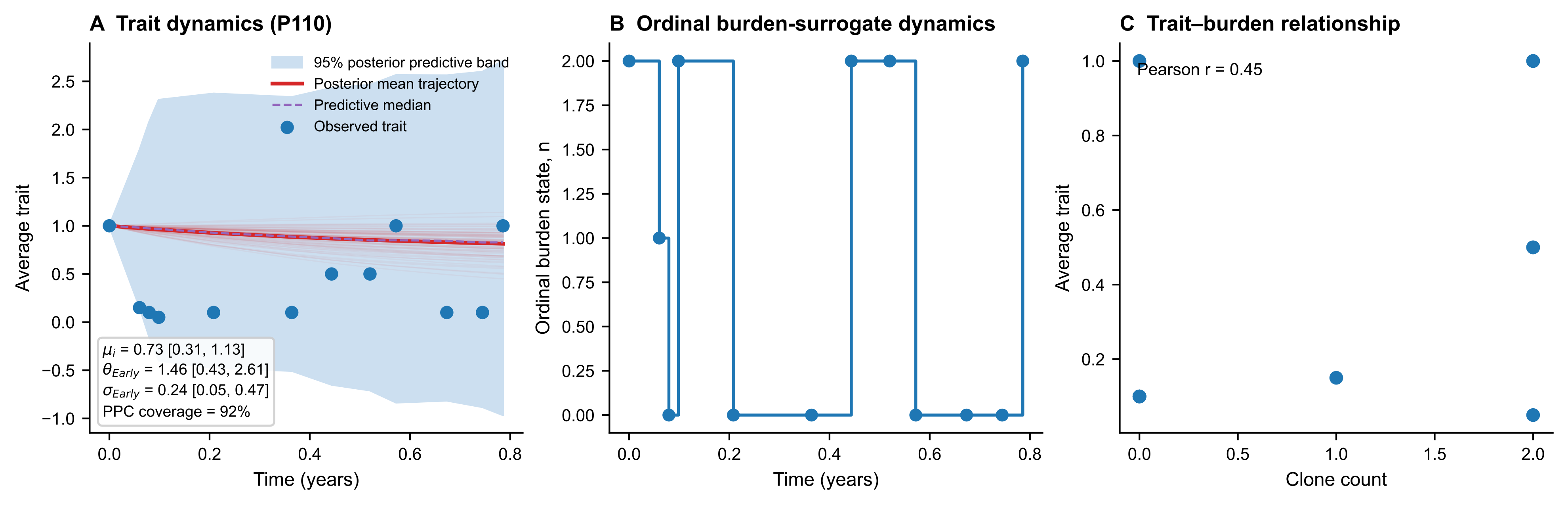

A Trait dynamics (P110)
95% posterior predictive band
Posterior mean trajectory
Predictive median
Observed trait
μ_i = 0.73 [0.31, 1.13]
θ_{Early} = 1.46 [0.43, 2.61]
σ_{Early} = 0.24 [0.05, 0.47]
PPC coverage = 92%
Average trait
Time (years)
B Ordinal burden-surrogate dynamics
Ordinal burden state, n
Time (years)
C Trait–burden relationship
Pearson r = 0.45
Average trait
Clone count

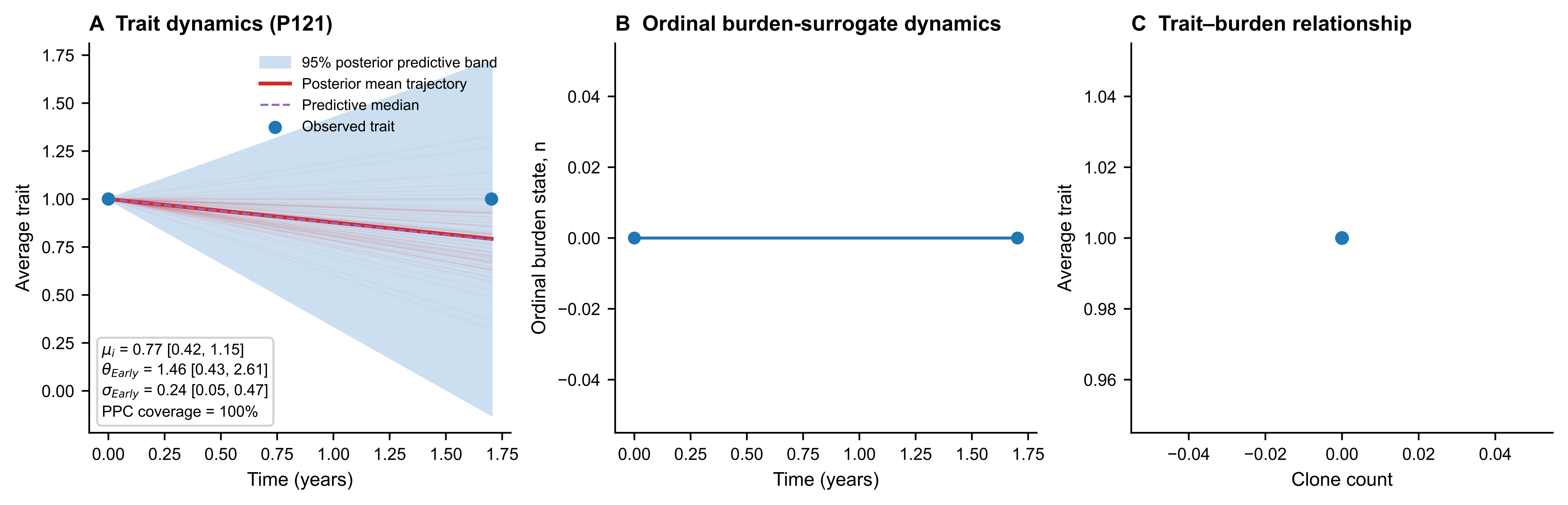
A Trait dynamics (P121)
95% posterior predictive band
Posterior mean trajectory
Predictive median
Observed trait
Average trait
Time (years)
μ_i = 0.77 [0.42, 1.15]
θ_{Early} = 1.46 [0.43, 2.61]
σ_{Early} = 0.24 [0.05, 0.47]
PPC coverage = 100%
B Ordinal burden-surrogate dynamics
Ordinal burden state, n
Time (years)
C Trait–burden relationship
Average trait
Clone count

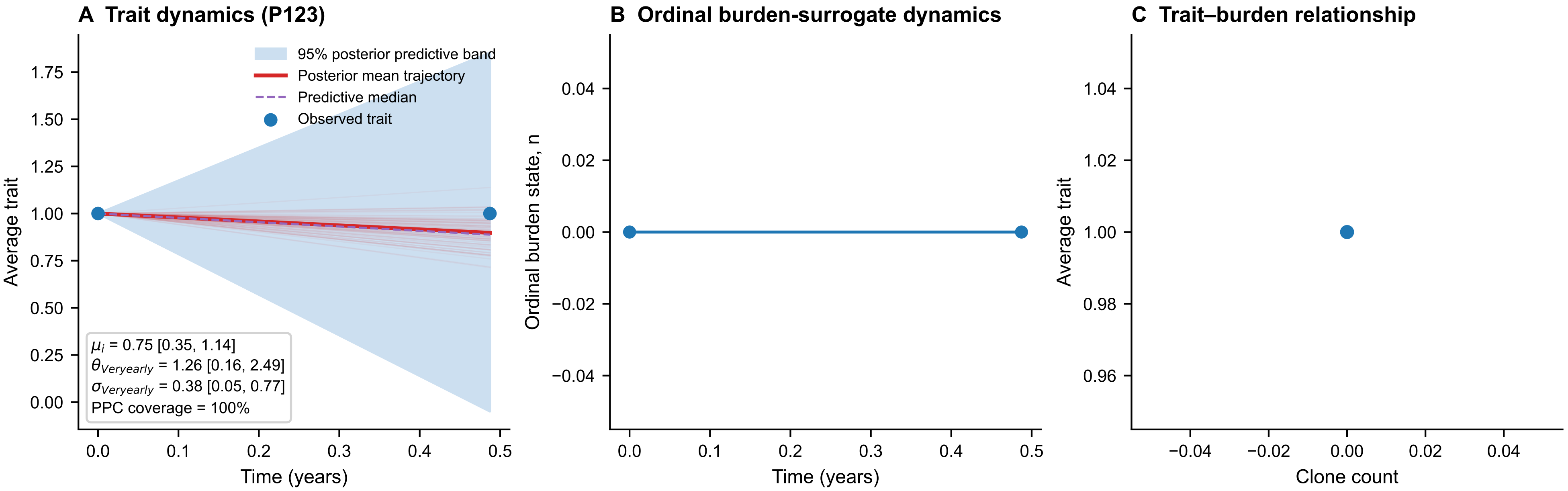
A Trait dynamics (P123)
95% posterior predictive band
Posterior mean trajectory
Predictive median
Observed trait
μ_i = 0.75 [0.35, 1.14]
$\theta_{Veryearly}$ = 1.26 [0.16, 2.49]
$\sigma_{Veryearly}$ = 0.38 [0.05, 0.77]
PPC coverage = 100%
Average trait
Time (years)
B Ordinal burden-surrogate dynamics
Ordinal burden state, n
Time (years)
C Trait–burden relationship
Average trait
Clone count

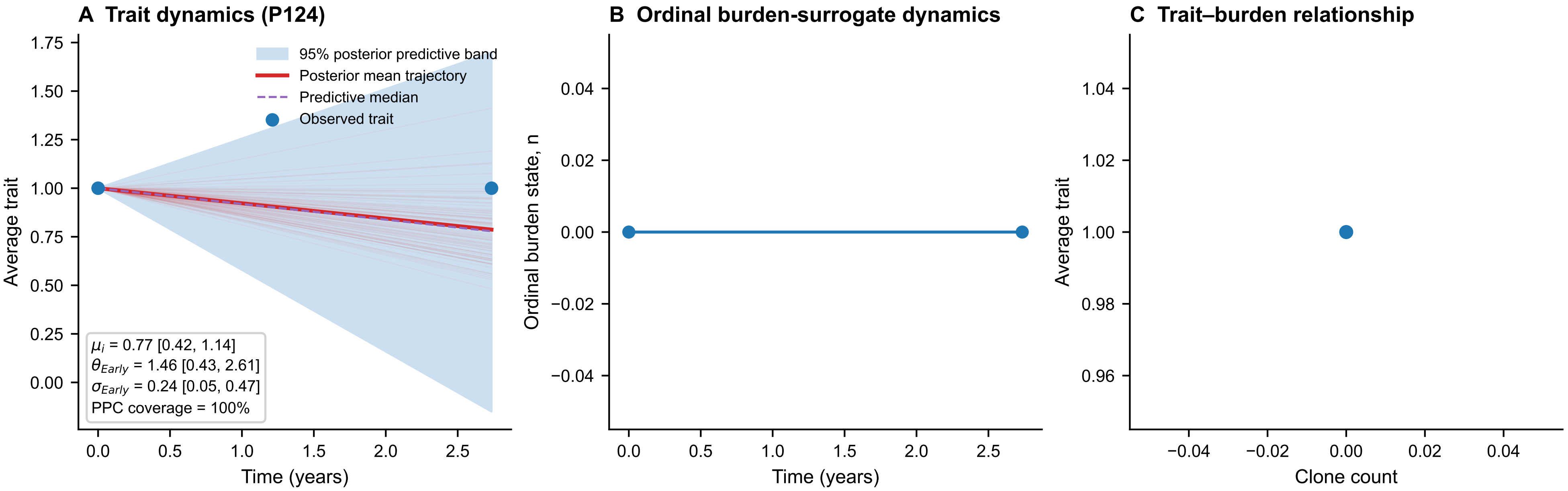

A Trait dynamics (P124)
95% posterior predictive band
Posterior mean trajectory
Predictive median
Observed trait
μ_i = 0.77 [0.42, 1.14]
θ_{Early} = 1.46 [0.43, 2.61]
σ_{Early} = 0.24 [0.05, 0.47]
PPC coverage = 100%
Average trait
Time (years)
B Ordinal burden-surrogate dynamics
Ordinal burden state, n
Time (years)
C Trait–burden relationship
Average trait
Clone count

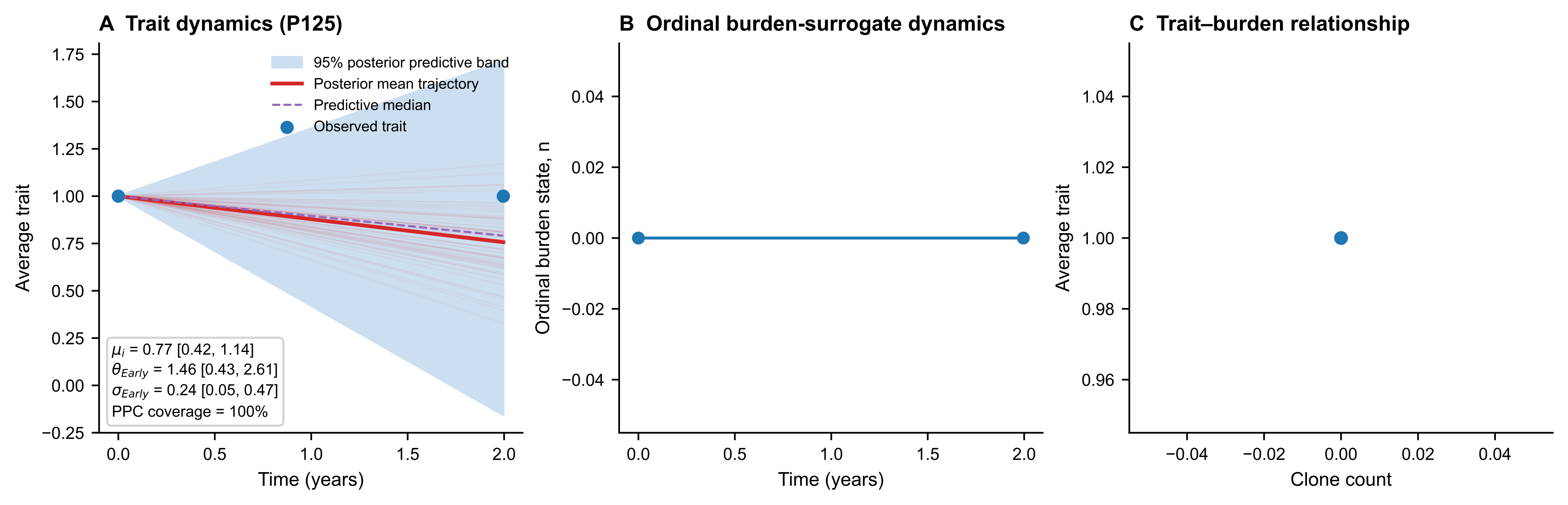
A Trait dynamics (P125)
95% posterior predictive band
Posterior mean trajectory
Predictive median
Observed trait
μ_i = 0.77 [0.42, 1.14]
θ_{Early} = 1.46 [0.43, 2.61]
σ_{Early} = 0.24 [0.05, 0.47]
PPC coverage = 100%
Average trait
Time (years)
B Ordinal burden-surrogate dynamics
Ordinal burden state, n
Time (years)
C Trait–burden relationship
Average trait
Clone count

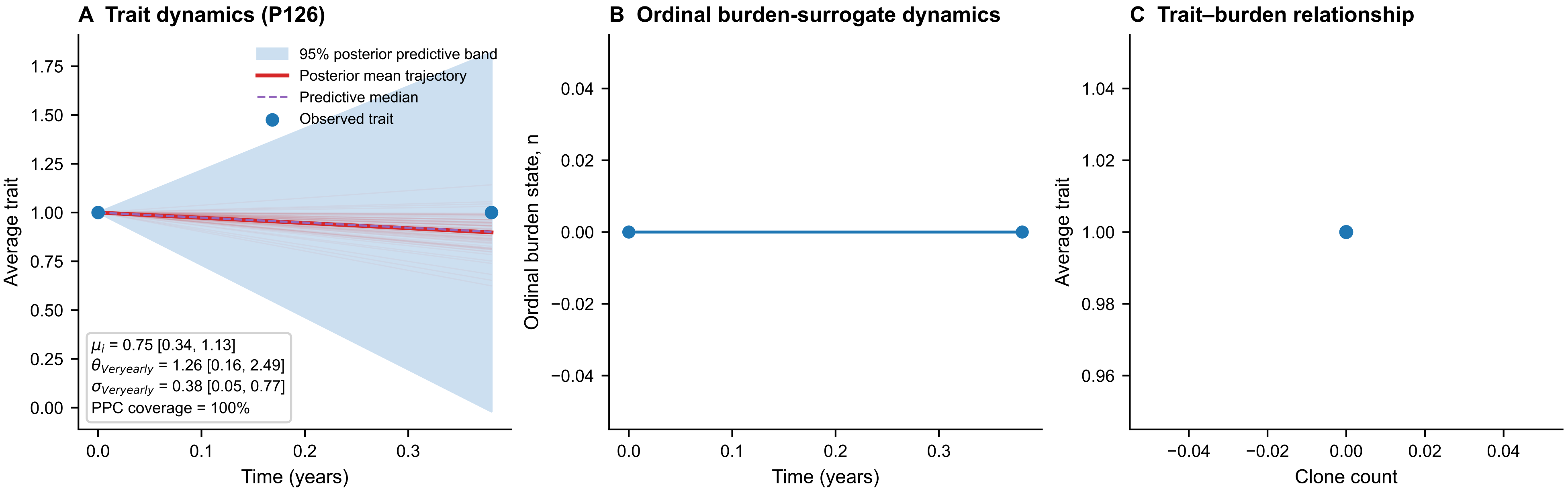

A Trait dynamics (P126)
95% posterior predictive band
Posterior mean trajectory
Predictive median
Observed trait
μ_i = 0.75 [0.34, 1.13]
$\theta_{Veryearly}$ = 1.26 [0.16, 2.49]
$\sigma_{Veryearly}$ = 0.38 [0.05, 0.77]
PPC coverage = 100%
Average trait
Time (years)
B Ordinal burden-surrogate dynamics
Ordinal burden state, n
Time (years)
C Trait–burden relationship
Average trait
Clone count

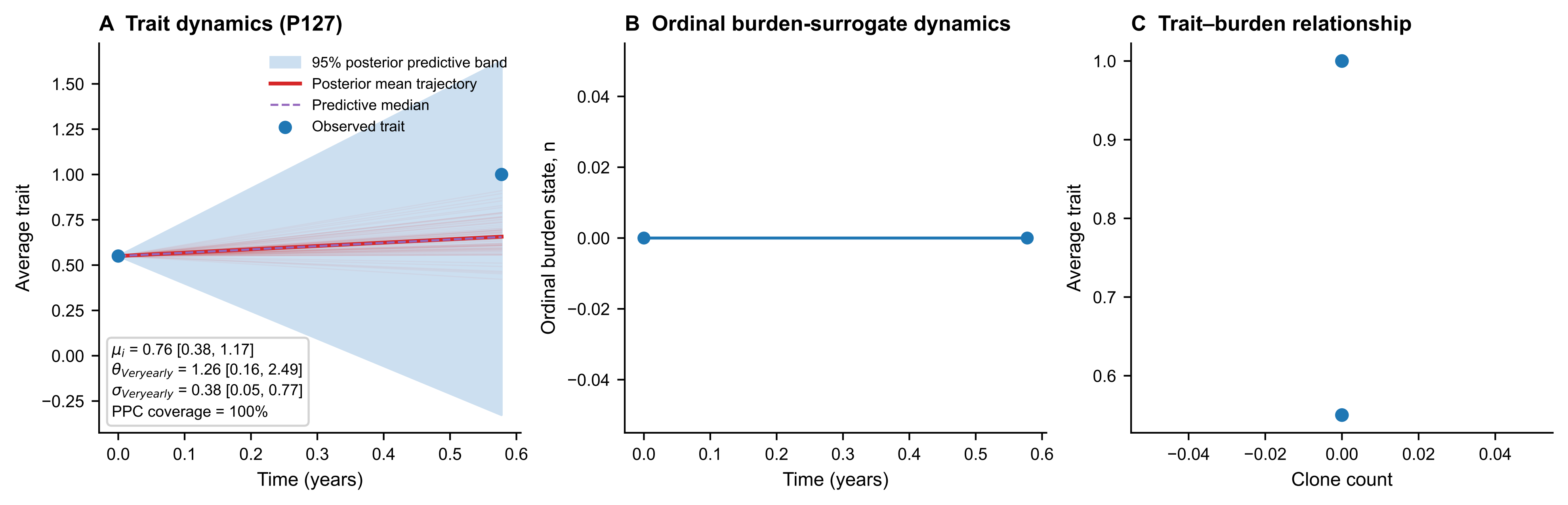

A Trait dynamics (P127)
95% posterior predictive band
Posterior mean trajectory
Predictive median
Observed trait
μ_i = 0.76 [0.38, 1.17]
$\theta_{Veryearly}$ = 1.26 [0.16, 2.49]
$\sigma_{Veryearly}$ = 0.38 [0.05, 0.77]
PPC coverage = 100%
Average trait
Time (years)
B Ordinal burden-surrogate dynamics
Ordinal burden state, n
Time (years)
C Trait–burden relationship
Average trait
Clone count

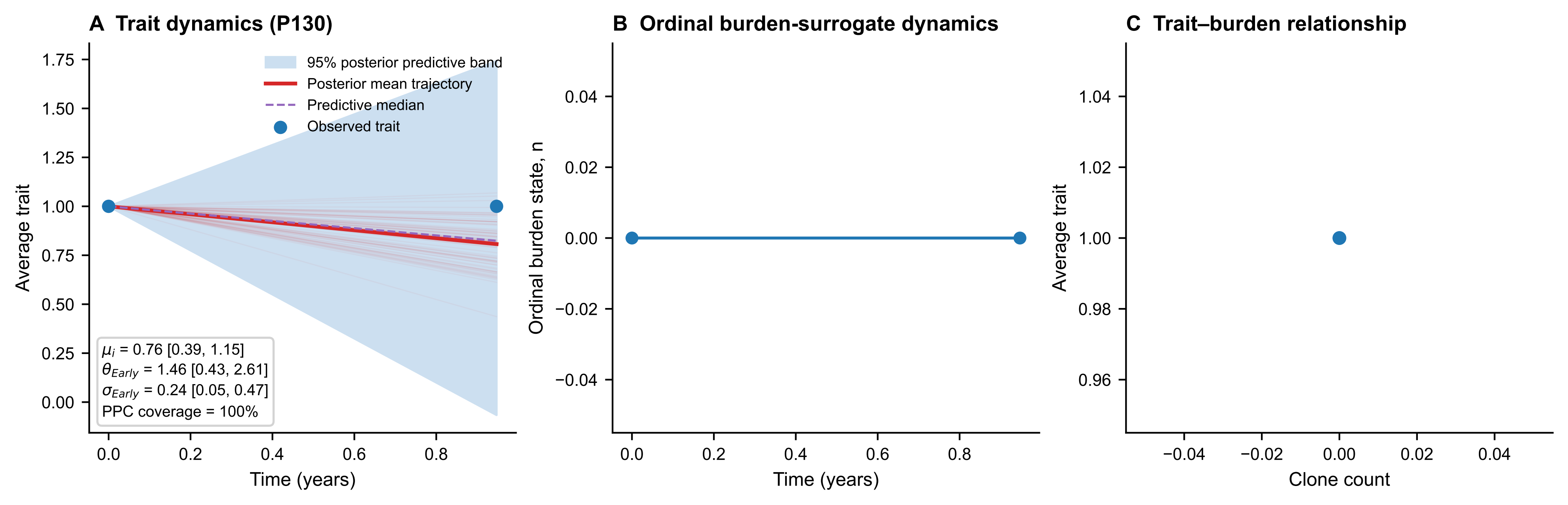
A Trait dynamics (P130)
95% posterior predictive band
Posterior mean trajectory
Predictive median
Observed trait
μ_i = 0.76 [0.39, 1.15]
θ_{Early} = 1.46 [0.43, 2.61]
σ_{Early} = 0.24 [0.05, 0.47]
PPC coverage = 100%
Average trait
Time (years)
B Ordinal burden-surrogate dynamics
Ordinal burden state, n
Time (years)
C Trait–burden relationship
Average trait
Clone count

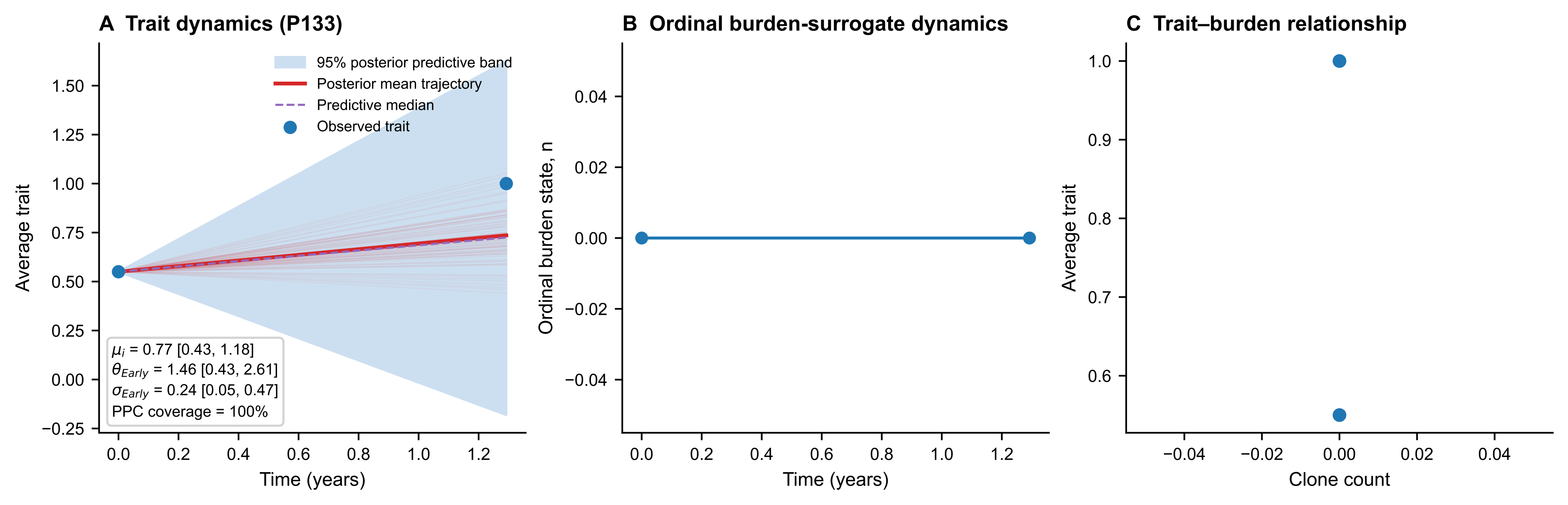

A Trait dynamics (P133)
95% posterior predictive band
Posterior mean trajectory
Predictive median
Observed trait
μ_i = 0.77 [0.43, 1.18]
θ_{Early} = 1.46 [0.43, 2.61]
σ_{Early} = 0.24 [0.05, 0.47]
PPC coverage = 100%
Average trait
Time (years)
B Ordinal burden-surrogate dynamics
Ordinal burden state, n
Time (years)
C Trait–burden relationship
Average trait
Clone count

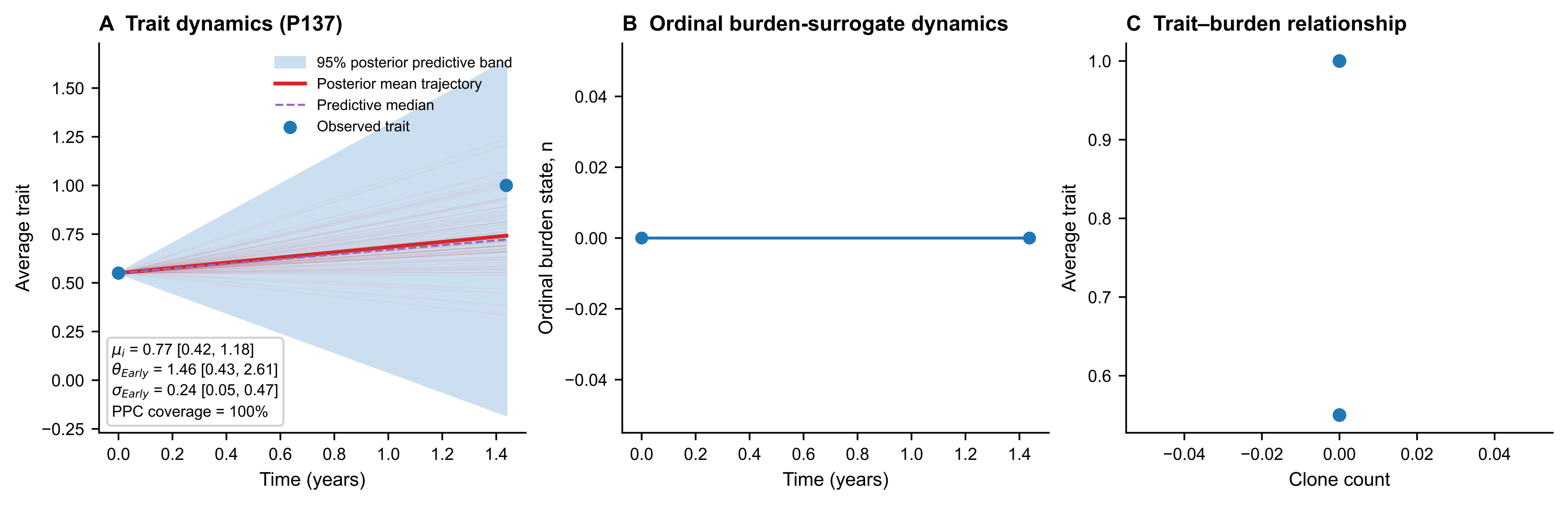

A Trait dynamics (P137)
95% posterior predictive band
Posterior mean trajectory
Predictive median
Observed trait
μ_i = 0.77 [0.42, 1.18]
θ_{Early} = 1.46 [0.43, 2.61]
σ_{Early} = 0.24 [0.05, 0.47]
PPC coverage = 100%
Average trait
Time (years)
B Ordinal burden-surrogate dynamics
Ordinal burden state, n
Time (years)
C Trait–burden relationship
Average trait
Clone count

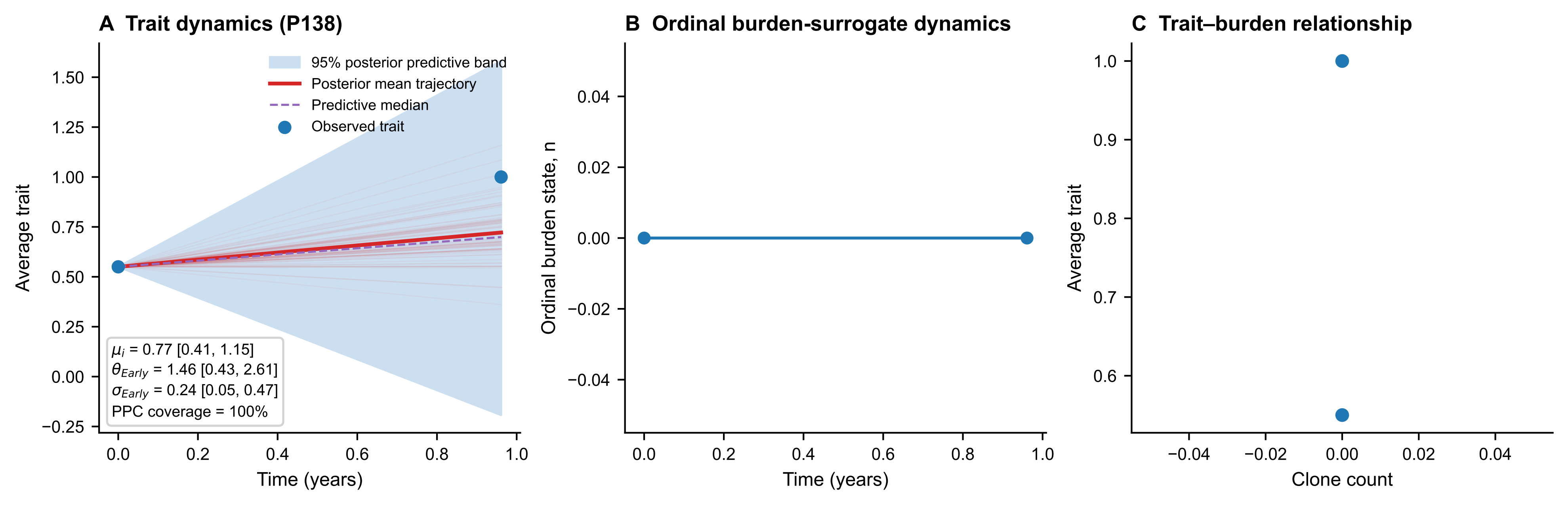

A Trait dynamics (P138)
95% posterior predictive band
Posterior mean trajectory
Predictive median
Observed trait
Average trait
Time (years)
μ_i = 0.77 [0.41, 1.15]
θ_{Early} = 1.46 [0.43, 2.61]
σ_{Early} = 0.24 [0.05, 0.47]
PPC coverage = 100%
B Ordinal burden-surrogate dynamics
Ordinal burden state, n
Time (years)
C Trait–burden relationship
Average trait
Clone count
