# Supplementary material for: Stage-specific evolutionary dynamics in pediatric leukemia inferred using Bayesian stochastic modeling
Source: PLoS One. 2026 Sep 11;21(9):e0357755. doi: 10.1371/journal.pone.0357755 (PMC13568502; doi:10.1371/journal.pone.0357755)
Supplement: Supplementary file 3 [file pone.0357755.s028.pdf]

Reviewer Comments

Manuscript Number: PONE-D-26-18451

Title: Stage-specific evolutionary dynamics in pediatric leukemia inferred using Bayesian stochastic modeling

This is an impressive and well-written manuscript. I really appreciate the thought and effort that went into developing this Bayesian framework to model tumor evolution using sparse, noisy longitudinal data. The application of the Ornstein-Uhlenbeck process is conceptually elegant and provides valuable biological interpretations regarding stage-specific disease dynamics. The validation through simulation is also greatly appreciated.

Given the clear value of your methodology, I have just a few very minor points for clarification to help round out the methods section:

- **Prior Distributions:** Could you please briefly add a sentence or two to the methods section clarifying the exact functional forms and hyperparameters used for the prior distributions of the OU parameters (μ, θ, and σ)? This will just ensure readers are fully confident in how the priors inform the results in the context of sparse data.
- **MCMC Convergence:** A quick sentence confirming your MCMC convergence metrics (for instance, just noting that R-hat values were near 1.0 and effective sample sizes were sufficient) would be a great, reassuring addition to the Bayesian calibration section.
- **Data Scaling:** In the "Data sources" section, the text mentions "within-patient scaling." Adding a brief half-sentence to specify the exact scaling method used (e.g., standardizing to mean zero and unit variance) would be a helpful detail for reproducibility.

Otherwise, fantastic work. I fully support its publication!
