# Supplementary material for: Stage-specific evolutionary dynamics in pediatric leukemia inferred using Bayesian stochastic modeling
Source: PLoS One. 2026 Sep 11;21(9):e0357755. doi: 10.1371/journal.pone.0357755 (PMC13568502; doi:10.1371/journal.pone.0357755)
Supplement: Supplementary file 4 [file pone.0357755.s030.pdf]

Response to Reviewers

Manuscript ID: PONE-D-26-18451

Title: *Stage-specific evolutionary dynamics in pediatric leukemia inferred using Bayesian stochastic modeling*

Dear Dr. Fujii,

We sincerely thank you and the reviewers for your careful evaluation of our manuscript and for the constructive comments. We greatly appreciate the opportunity to revise the manuscript. We have carefully considered every comment and substantially revised the manuscript to improve methodological transparency, reproducibility, statistical interpretation, and biological caution. Major revisions include:

- substantially expanded Methods with complete mathematical specification of the hierarchical Bayesian OU model,
- explicit presentation of the OU stochastic differential equation and exact transition density,
- complete description of prior distributions, parameter constraints, posterior computation, initialization, convergence diagnostics, and software environment,
- extensive simulation-based calibration and estimator robustness analyses,
- detailed characterization of longitudinal sampling density and irregularity,
- expanded posterior predictive diagnostics,
- substantially tempered biological interpretation of OU parameters,
- revised figures, supplementary information, and reproducibility documentation,
- public release of the complete analysis pipeline through GitHub and a permanent Zenodo archive.

All reviewer comments are reproduced below in **bold**, followed by our responses.

Reviewer #1

We sincerely thank the reviewer for the thoughtful and detailed comments. The suggestions substantially improved the clarity and transparency of the manuscript.

Major Comment 1

The statistical framework lacks mathematical and computational details necessary for rigorous evaluation and reproducibility.

Response

We appreciate this important suggestion.

The Methods section has been extensively expanded on pages 4–19, lines 80–467.

Specifically, we have now added:

- the complete Ornstein–Uhlenbeck stochastic differential equation,
- the exact OU transition density used for irregular sampling,
- the hierarchical Bayesian model formulation,
- explicit likelihood equations,
- complete prior distributions including hyperparameters,
- parameter constraints,
- residual transition-scale parameter,
- posterior computation details,
- NUTS configuration,
- initialization strategy,
- convergence diagnostics,
- software versions,
- Conda environment,
- GitHub repository,
- permanent Zenodo archive,
- complete computational workflow.

These revisions substantially improve methodological transparency and reproducibility.

Major Comment 2

Biological interpretation of OU parameters should be tempered.

Response

We fully agree.

The statistical interpretation of the OU parameters is clarified in the Methods on pages 9–11, lines 200–268, and the biological limitations are discussed on pages 25–26, lines 614–642.

Throughout the manuscript we now consistently describe

- μ as the effective equilibrium state,
- θ as an effective mean-reversion parameter,
- σ as effective stochastic variability.

We removed or substantially softened previous mechanistic language such as

- “restoration of stabilizing constraints,”
- “evolutionary instability,”
- “phenotypic dispersion.”

Instead, we now explicitly state throughout the Methods, Results, and Discussion that these parameters are **effective statistical summaries under the assumed OU model** rather than direct measurements of biological mechanisms or causal evolutionary forces.

Major Comment 3

Parameter identifiability should be demonstrated more quantitatively.

Response

We substantially expanded both the empirical sampling characterization and the simulation benchmark. The distribution of observation counts, follow-up durations, consecutive sampling intervals, and patient-level sampling irregularity is now described on pages 7–8, lines 170–198, and summarized in Fig S1 and Table S1. The simulation design, estimation methods, performance metrics, and prespecified reliability criteria are described on pages 14–15, lines 335–368. Quantitative recovery and reliability results are reported on pages 20–21, lines 471–501, in Fig 2 and Tables S2–S3. The Discussion now explicitly distinguishes computational convergence from statistical identifiability on pages 28–29, lines 693–711.

Major Comment 4

Clinical-stage definitions should be clarified.

Response

We agree that the origin and use of the clinical-stage categories required clarification. These groups were not newly defined in the present study. Rather, we adopted the predefined clinical-group labels reported in the original KMT2A-rearranged leukemia cohort of Ahlgren et al. [1]. We have now clarified this in the Methods and state explicitly that the present analysis did not redefine or independently reconstruct the Early, Early/refractory, Late, Remission, Very early, and Very early/refractory categories. Each modeled patient retained the single group label provided in the source dataset for hierarchical Bayesian inference. The complete patient-to-group mapping is provided in S4 Data, and group-specific sample sizes are summarized in Table S4 (page 7, lines 160–168).

Major Comments 5–6

Poor predictive performance and alternative stochastic processes should be discussed more carefully.

Response

We agree that the distinction among parameter inference, uncertainty calibration, and point prediction required clearer treatment. We therefore added a cohort-wide posterior predictive analysis for all 47 modeled patients. Mean coverage of the nominal 95% posterior predictive interval was approximately 94%, and median patient-level coverage was 100%, although coverage varied across groups. Predictive R^2 was negative for many patients, indicating that the posterior-mean trajectory often reconstructed the observed series less accurately than the patient-specific observed mean. We now explicitly state that this finding limits point-trajectory

reconstruction but does not imply failed MCMC convergence or invalidate the posterior distributions of the OU parameters (pages 24–25, lines 570–610, Fig 5, and Tables S10–S12).

We also added a dedicated Discussion subsection that distinguishes posterior parameter inference, posterior predictive coverage, and point-trajectory reconstruction on pages 27–28, lines 666–691. Alternative stochastic formulations—including Brownian motion, switching OU models, hidden Markov models, nonlinear stochastic differential equations, jump or Lévy processes, and explicit branching models—are discussed on pages 29-30, lines 715–730.

Minor Comment 1

Present the OU stochastic differential equation explicitly.

Response

We have added the complete OU stochastic differential equation and exact conditional transition distribution to the Methods (pages 9–11, lines 200–268).

The complete stochastic differential equation is now presented in the Methods together with its biological interpretation and exact transition density.

Minor Comment 2

Improve figure resolution.

Response

All figures were regenerated and will be submitted as separate publication-quality TIFF files at the resolution required by PLOS ONE. Text, line weights, and panel labels were also enlarged to improve readability.

Minor Comment 3

Improve terminology consistency.

Response

The terminology has been standardized throughout the manuscript.
The revised manuscript consistently uses:

- effective mean reversion,
- stochastic variability,
- drift-to-noise ratio,
- effective dynamical characteristics.

Mechanistic terminology has been substantially reduced.

Minor Comment 4

Better distinguish prediction, reconstruction, and mechanistic interpretation.

Response
A dedicated Discussion subsection now explicitly distinguishes these concepts and explains their different interpretations.

Minor Comment 5
Quantify posterior interval coverage.

Response
The Results now report mean coverage of approximately 94% for the nominal 95% posterior predictive interval and median patient-level coverage of 100% (page 24, lines 572–579). Patient-level, group-level, and correlation-based diagnostics are provided in Fig 5 and Tables S10–S12.

Ethics statement
The ethics statement should be consistent.

Response
We corrected the online submission metadata so that it is fully consistent with the manuscript. The study used previously published, publicly available data and involved no new participant recruitment or collection of identifiable private information. The revised ethics statement now accurately reflects the status of the secondary data analysis.

Reviewer #2
We sincerely thank the reviewer for the encouraging evaluation and constructive suggestions.

Prior distributions
Please clarify prior distributions.

Response
The Methods now explicitly specify the prior distributions, hyperparameters, parameter constraints, hierarchical parameterization, and residual transition-scale prior on pages 11–13, lines 270–305.

MCMC convergence
Please report convergence metrics.

Response
We now report four chains, 2,500 tuning iterations and 2,000 retained draws per chain, the *jitter+adapt_diag* initialization strategy, target acceptance probability, $\hat{R}$, bulk and tail effective sample sizes, divergent transitions, and tree-depth events on pages 13–14, lines 307–333. Complete convergence summaries are also provided as S13 Data, with run metadata in S14 Data.

The Results report a maximum $\hat{R}$ of 1.003, minimum bulk and tail ESS values exceeding 4,100, no divergent transitions, and no maximum tree-depth events (page 21, lines 503–510).

Data scaling

Clarify within-patient scaling.

Response

We clarified that no within-patient centering, z-score transformation, unit-variance standardization, or other within-patient rescaling was applied. The longitudinal x and n variables were constructed deterministically from the clinical annotations according to the stated rules and entered the analysis without additional rescaling (pages 6–7, lines 127–158).

Additional Journal Requirements

Formatting

The manuscript has been revised to conform to the current PLOS ONE formatting requirements.

Code availability

The complete computational pipeline is now openly available through GitHub.

A permanent archived version has also been deposited on Zenodo.

Data availability

The online Data Availability Statement has been revised to provide the DOI and available direct source information for the original KMT2A-rearranged leukemia cohort. The permanent Zenodo archive contains the processed model inputs, simulation outputs, posterior summaries, convergence diagnostics, and reproducibility files generated for the present study, while the unrestricted analysis code is available through GitHub.

Related manuscript

The previously mentioned related manuscript is:

“Kim SH. Hierarchical Bayesian OU–Branching Models Capture Stochastic and Deterministic Forces in Bacterial Mutation Evolution.”

This manuscript is currently under consideration at Bioinformatics Advances and has not been published.

The present manuscript remains entirely distinct.

Specifically:

- it analyzes an independent pediatric leukemia dataset,
- it addresses a different biological question,
- it contains different figures,
- it contains different simulations,
- it develops a substantially expanded hierarchical Bayesian OU framework focused on stage-associated tumor evolution rather than bacterial mutation dynamics.

Accordingly, there is no overlap in datasets, analyses, figures, or conclusions, and the present manuscript does not constitute duplicate publication.

We again sincerely thank the Academic Editor and both reviewers for their careful reading and highly constructive suggestions. Their comments have substantially strengthened the manuscript. We hope that the revised version satisfactorily addresses all concerns and will now be suitable for publication in **PLOS ONE**.

Sincerely,

Seung-Hwan Kim
