# Supplementary material for: Stage-specific evolutionary dynamics in pediatric leukemia inferred using Bayesian stochastic modeling
Source: PLoS One. 2026 Sep 11;21(9):e0357755. doi: 10.1371/journal.pone.0357755 (PMC13568502; doi:10.1371/journal.pone.0357755)
Supplement: Supplementary file 5 [file pone.0357755.s031.pdf]

Response to Reviewers

Manuscript ID: PONE-D-26-18451R2

Title: *Stage-specific evolutionary dynamics in pediatric leukemia inferred using Bayesian stochastic modeling*

Dear Dr. Fujii,

We sincerely thank you and the reviewers for your careful evaluation of our revised manuscript. We greatly appreciate the reviewers' positive assessment that our previous revisions had addressed the major concerns, as well as the constructive suggestions that further improved the manuscript.

In response to the remaining comments, we have revised the manuscript to improve clarity, readability, and the presentation of the methodological framework. No additional analyses were required, and the scientific conclusions remain unchanged. All changes have been incorporated into both the marked-up and clean versions of the manuscript.

Our detailed responses are provided below.

Academic Editor
We sincerely thank the Academic Editor for the careful evaluation of our revised manuscript and for the opportunity to submit a further minor revision. We appreciate the Editor's assessment that the manuscript has merit and that the remaining issues primarily concern clarification and presentation. We have carefully addressed all comments raised by Reviewer #1.

Reviewer #1
We sincerely thank the reviewer for the thoughtful evaluation of our revised manuscript and for recognizing that the major comments from the previous review had been satisfactorily addressed. We appreciate these constructive suggestions, which have further improved the clarity and accessibility of the manuscript.

Comment 1
"Add a summary table describing the notation of the major model parameters to improve readability."

Response
We appreciate this helpful suggestion.

To improve readability, we have added **Table 1 ("Summary of notation used throughout the hierarchical OU model")** immediately following the Study Overview section in the Methods (page 5, lines 110-114). The table summarizes the principal mathematical symbols used throughout the manuscript, including the modeled longitudinal state, observation variables, equilibrium state, mean-reversion rate, diffusion scale, residual transition parameter, observation interval, and Wiener process.

In addition, we now explicitly refer readers to this table at the end of the Study Overview.

Comment 2

“The Discussion appropriately acknowledges alternative stochastic processes, but the manuscript does not clearly explain why the OU process is particularly appropriate for the present biological problem beyond its mean-reverting property. A more balanced comparison of the modeling assumptions, advantages, and limitations of the OU process relative to alternative approaches would strengthen the methodological justification.”

Response

Thank you for this valuable suggestion.

We have expanded the Discussion to provide a more explicit justification for selecting the Ornstein–Uhlenbeck process for sparse longitudinal leukemia data (page 27, lines 638-645). The revised text now explains that leukemia progression is expected to evolve under biological constraints imposed by developmental programs, therapeutic intervention, immune surveillance, and related regulatory processes rather than unrestricted random diffusion. We further contrast the OU process with Brownian motion and discuss why its parsimonious formulation is advantageous under sparse and irregular sampling.

We have also expanded the discussion of alternative stochastic modeling approaches, including Brownian motion, hidden Markov models, nonlinear stochastic differential equations, Lévy jump processes, and branching processes (page 31, lines 745-752). The revised manuscript emphasizes that these approaches should be viewed as complementary rather than competing methodologies and that model selection should ultimately be guided by the biological question, data availability, and statistical identifiability rather than by any single universally optimal stochastic formulation.

Comment 3

“The study is based on a single pediatric KMT2A-rearranged leukemia cohort consisting of 52 patients. Although the authors appropriately acknowledge the limitations of sparse sampling, the Discussion could more explicitly address whether the proposed framework is expected to generalize to other leukemia subtypes, adult malignancies, or longitudinal cancer datasets with different sampling structures.”

Response

We appreciate this important suggestion.

To address this point, we have added a new subsection entitled **“Generalizability**” (pages 31-32, lines 754-765). In this section, we clarify that although the present application focuses on pediatric KMT2A-rearranged leukemia, the Bayesian Ornstein–Uhlenbeck framework itself is not disease specific. We discuss its potential applicability to other pediatric leukemias, adult hematologic malignancies, solid tumors, and additional longitudinal biological datasets that exhibit constrained stochastic dynamics.

At the same time, we explicitly note that successful application to other disease settings will require independent validation and careful evaluation of the underlying modeling assumptions, sampling density, and observation process.

Comment 4

“Throughout the manuscript, the authors emphasize that the study is intended primarily as a statistical modeling framework rather than a predictive model. Nevertheless, the Discussion occasionally shifts between describing methodological advances and biological interpretation. A clearer separation between (i) methodological contributions, (ii) biological observations supported

by the current data, and (iii) future applications would improve the overall narrative and help readers understand the principal contribution of the study."

Response
We thank the reviewer for this excellent recommendation.

To improve the overall organization of the Discussion, we have substantially reorganized the Discussion to provide a clearer distinction between methodological contributions, biological observations, future applications, limitations, and the concluding interpretation (pages 32-35, lines 767-863). The revised manuscript now clearly separates:

- **Methodological implications**, emphasizing the statistical contributions of the Bayesian hierarchical OU framework and uncertainty-aware inference;
- **Biological observations**, summarizing the descriptive findings supported by the present dataset while carefully avoiding mechanistic overinterpretation;
- **Future applications**, discussing potential extensions to additional disease settings and more flexible stochastic modeling frameworks;
- **Limitations**, presenting the remaining methodological constraints of the current study; and
- **Conclusion**, emphasizing that the principal contribution of this work is an interpretable and uncertainty-aware statistical framework rather than a predictive or mechanistic model.

We believe that this revised organization substantially improves the clarity of the manuscript and more clearly distinguishes methodological contributions from biological interpretation, as suggested.

Reviewer #2
We sincerely thank the reviewer for the positive evaluation of our revised manuscript and for indicating that all previous comments had been satisfactorily addressed. We greatly appreciate the reviewer's careful assessment and encouraging recommendation.

Once again, we sincerely thank the Academic Editor and both reviewers for their thoughtful evaluation of our work. We believe that the revisions have substantially improved the clarity and accessibility of the manuscript, and we hope that the revised version is now suitable for publication in **PLOS ONE**.

Sincerely,

Seung-Hwan Kim, Ph.D.
Department of Biology, Fisher College
Department of Pediatric Oncology, Dana-Farber Cancer Institute
