# Supplementary material for: Stage-specific evolutionary dynamics in pediatric leukemia inferred using Bayesian stochastic modeling
Source: PLoS One. 2026 Sep 11;21(9):e0357755. doi: 10.1371/journal.pone.0357755 (PMC13568502; doi:10.1371/journal.pone.0357755)
Supplement: Peer review history

# Peer review: Decision Letter 0

**DOI:** 10.1371/journal.pone.0357755.r001

**Authors:** Hodaka Fujii

15 Jul 2026

Dear Dr. Kim,

Thank you for submitting your manuscript to PLOS ONE. After careful consideration, we feel that it has merit but does not fully meet PLOS ONE’s publication criteria as it currently stands. Therefore, we invite you to submit a revised version of the manuscript that addresses the points raised during the review process.

Please submit your revised manuscript by Aug 29 2026 11:59PM. If you will need more time than this to complete your revisions, please reply to this message or contact the journal office at plosone@plos.org. When you're ready to submit your revision, log on to https://www.editorialmanager.com/pone/ and select the 'Submissions Needing Revision' folder to locate your manuscript file.

- A letter that responds to each point raised by the academic editor and reviewer(s). You should upload this letter as a separate file labeled 'Response to Reviewers'.
- A marked-up copy of your manuscript that highlights changes made to the original version. You should upload this as a separate file labeled 'Revised Manuscript with Track Changes'.
- An unmarked version of your revised paper without tracked changes. You should upload this as a separate file labeled 'Manuscript'.

If you would like to make changes to your financial disclosure, please include your updated statement in your cover letter. Guidelines for resubmitting your figure files are available below the reviewer comments at the end of this letter.

If applicable, we recommend that you deposit your laboratory protocols in protocols.io to enhance the reproducibility of your results. Protocols.io assigns your protocol its own identifier (DOI) so that it can be cited independently in the future. For instructions see: https://journals.plos.org/plosone/s/submission-guidelines#loc-laboratory-protocols. Additionally, PLOS ONE offers an option for publishing peer-reviewed Lab Protocol articles, which describe protocols hosted on protocols.io. Read more information on sharing protocols at https://plos.org/protocols?utm_medium=editorial-email&utm_source=authorletters&utm_campaign=protocols.

As the corresponding author, your ORCID iD is verified in the submission system and will appear in the published article. PLOS supports the use of ORCID, and we encourage all coauthors to register for an ORCID iD and use it as well. Please encourage your coauthors to verify their ORCID iD within the submission system before final acceptance, as unverified ORCID iDs will not appear in the published article. *Only*  the individual author can complete the verification step; PLOS staff *cannot*  verify ORCID iDs on behalf of authors.

We look forward to receiving your revised manuscript.

Kind regards,

Hodaka Fujii, M.D., Ph.D.

Academic Editor

PLOS One

Journal Requirements:

When submitting your revision, we need you to address these additional requirements.

1. Please ensure that your manuscript meets PLOS ONE's style requirements, including those for file naming. The PLOS ONE style templates can be found at https://journals.plos.org/plosone/s/file?id=wjVg/PLOSOne_formatting_sample_main_body.pdf and https://journals.plos.org/plosone/s/file?id=ba62/PLOSOne_formatting_sample_title_authors_affiliations.pdf

2. Please note that PLOS One has specific guidelines on code sharing for submissions in which author-generated code underpins the findings in the manuscript. In these cases, all author-generated code must be made available without restrictions upon publication of the work. Please review our guidelines at https://journals.plos.org/plosone/s/materials-and-software-sharing#loc-sharing-code and ensure that your code is shared in a way that follows best practice and facilitates reproducibility and reuse.

3. Please note that your Data Availability Statement in the online submission form is currently missing the DOI/accession number of each dataset, OR a direct link to access each database. If your manuscript is accepted for publication, you will be asked to provide these details on a very short timeline. We therefore suggest that you provide this information now, though we will not hold up the peer review process if you are unable.

If the reviewer comments include a recommendation to cite specific previously published works, please review and evaluate these publications to determine whether they are relevant and should be cited. There is no requirement to cite these works unless the editor has indicated otherwise.

[Note: HTML markup is below. Please do not edit.]

Reviewers' comments:

Reviewer's Responses to Questions

**Comments to the Author**

1. Is the manuscript technically sound, and do the data support the conclusions?

Reviewer #1: Partly

Reviewer #2: Yes

**********

2. Has the statistical analysis been performed appropriately and rigorously?

Reviewer #1: No

Reviewer #2: Yes

**********

3. Have the authors made all data underlying the findings in their manuscript fully available?

The PLOS Data policy

Reviewer #1: No

Reviewer #2: Yes

**********

4. Is the manuscript presented in an intelligible fashion and written in standard English?

Reviewer #1: Yes

Reviewer #2: Yes

**********

Reviewer #1: The manuscript presents a Bayesian Ornstein–Uhlenbeck (OU) stochastic modeling framework to characterize longitudinal tumor evolution dynamics in pediatric KMT2A-rearranged leukemia using sparse clinical time-series data. The topic is potentially interesting and the manuscript is generally well organized, but several methodological and interpretational issues require clarification before the work can be considered for publication.

Major Comments:

1. The statistical framework is described largely conceptually, but the manuscript lacks the mathematical and computational details necessary for rigorous evaluation and reproducibility. Key Bayesian modelling details are missing (e.g., prior distribution of all parameters, parameter constraints, initialization strategies, convergence diagnostics, etc.)

2. The manuscript repeatedly interprets OU parameters biologically, for example associating stronger mean reversion with “restoration of stabilizing constraints” and larger stochasticity with “evolutionary instability.” However, the presented analyses remain largely descriptive and correlative. The current evidence does not establish that the inferred parameters correspond to mechanistic biological processes rather than statistical artifacts induced by sampling density, scaling, or treatment timing.

It is recommended the authors either substantially temper these mechanistic interpretations or provide stronger justification.

3. A major challenge in OU modeling with sparse longitudinal data is parameter identifiability, especially for θ, the mean reversion strength and σ, diffusion term. Although the manuscript acknowledges this issue, the simulation study remains too high-level and does not quantitatively demonstrate identifiability boundaries. The reviewer suggest the authors to report: the distribution of timepoints per patient, sampling interval heterogeneity, simulation settings used for validation, parameter recovery bias and variance, etc.

4. The manuscript stratifies patients into groups such as “early,” “late,” “remission,” and “early/refractory,” but the definitions of these categories are insufficiently described. The authors should clarify how each clinical stage is defined, sample size per group, and whether these groups are mutually exclusive.

5. The manuscript acknowledges poor predictive performance, including negative Bayesian R² values and under-dispersed posterior predictive intervals.

6. Negative Bayesian R² values suggest the model may explain less variability than a baseline model for some patients, raising questions about whether the OU process is appropriate for these data. The manuscript should discuss why OU dynamics remain preferable despite weak predictive fit/whether alternative stochastic processes were considered, and whether nonlinear or switching-state models may better capture leukemia evolution.

Minor Comments:

1. Method Section: The manuscript would benefit from explicitly presenting the OU stochastic differential equation in the main text rather than relying on conceptual descriptions alone.

2. All figures have very low resolution. Please present results in higher resolution.

3. The manuscript occasionally uses terminology inconsistently, alternating between “stochastic variability,” “drift-to-noise,” “evolutionary instability,” and “phenotypic dispersion.” A clearer terminology framework would improve readability.

4. The Discussion could better distinguish between “prediction,” “trajectory reconstruction,” and “mechanistic interpretation,” which are currently somewhat conflated.

5. The statement “posterior interval coverage approached nominal levels” should ideally include quantitative values or references to supplementary results.

The ethics statement in the submission metadata lists “N/A,” whereas the manuscript later includes a detailed ethics statement. These should be made fully consistent.

Reviewer #2: This is an impressive and well-written manuscript. I really appreciate the thought and effort that went into developing this Bayesian framework to model tumor evolution using sparse, noisy longitudinal data. The application of the Ornstein-Uhlenbeck process is conceptually elegant and provides valuable biological interpretations regarding stage-specific disease dynamics. The validation through simulation is also greatly appreciated.

Given the clear value of your methodology, I have just a few very minor points for clarification to help round out the methods section:

Prior Distributions: Could you please briefly add a sentence or two to the methods section clarifying the exact functional forms and hyperparameters used for the prior distributions of the OU parameters (µ, θ, and σ)? This will just ensure readers are fully confident in how the priors inform the results in the context of sparse data.

MCMC Convergence: A quick sentence confirming your MCMC convergence metrics (for instance, just noting that R-hat values were near 1.0 and effective sample sizes were sufficient) would be a great, reassuring addition to the Bayesian calibration section.

Data Scaling: In the "Data sources" section, the text mentions "within-patient scaling." Adding a brief half-sentence to specify the exact scaling method used (e.g., standardizing to mean zero and unit variance) would be a helpful detail for reproducibility.

Otherwise, fantastic work.

**********

what does this mean?). If published, this will include your full peer review and any attached files.

If you choose “no”, your identity will remain anonymous but your review may still be made public.

**Do you want your identity to be public for this peer review?**  For information about this choice, including consent withdrawal, please see our Privacy Policy

Reviewer #1: **Yes:** Yuhang Liu

Reviewer #2: No

**********

[NOTE: If reviewer comments were submitted as an attachment file, they will be attached to this email and accessible via the submission site. Please log into your account, locate the manuscript record, and check for the action link "View Attachments". If this link does not appear, there are no attachment files.]

To ensure your figures meet our technical requirements, please review our figure guidelines: https://journals.plos.org/plosone/s/figures

You may also use PLOS’s free figure tool, NAAS, to help you prepare publication quality figures: https://journals.plos.org/plosone/s/figures#loc-tools-for-figure-preparation.

NAAS will assess whether your figures meet our technical requirements by comparing each figure against our figure specifications.

Attachment Submitted filename: Review_Comments_PONE-D-26-18451.pdf

# Author comment: Author response to Decision Letter 1

**DOI:** 10.1371/journal.pone.0357755.r002

20 Jul 2026

Response to Reviewers

Manuscript ID: PONE-D-26-18451

Title: Stage-specific evolutionary dynamics in pediatric leukemia inferred using Bayesian stochastic modeling

Dear Dr. Fujii,

We sincerely thank you and the reviewers for your careful evaluation of our manuscript and for the constructive comments. We greatly appreciate the opportunity to revise the manuscript. We have carefully considered every comment and substantially revised the manuscript to improve methodological transparency, reproducibility, statistical interpretation, and biological caution.

Major revisions include:

substantially expanded Methods with complete mathematical specification of the hierarchical Bayesian OU model,

explicit presentation of the OU stochastic differential equation and exact transition density,

complete description of prior distributions, parameter constraints, posterior computation, initialization, convergence diagnostics, and software environment,

extensive simulation-based calibration and estimator robustness analyses,

detailed characterization of longitudinal sampling density and irregularity,

expanded posterior predictive diagnostics,

substantially tempered biological interpretation of OU parameters,

revised figures, supplementary information, and reproducibility documentation,

public release of the complete analysis pipeline through GitHub and a permanent Zenodo archive.

All reviewer comments are reproduced below in bold, followed by our responses.

Reviewer #1

We sincerely thank the reviewer for the thoughtful and detailed comments. The suggestions substantially improved the clarity and transparency of the manuscript.

Major Comment 1

The statistical framework lacks mathematical and computational details necessary for rigorous evaluation and reproducibility.

Response

We appreciate this important suggestion.

The Methods section has been extensively expanded on pages 4–19, lines 80–467.

Specifically, we have now added:

the complete Ornstein–Uhlenbeck stochastic differential equation,

the exact OU transition density used for irregular sampling,

the hierarchical Bayesian model formulation,

explicit likelihood equations,

complete prior distributions including hyperparameters,

parameter constraints,

residual transition-scale parameter,

posterior computation details,

NUTS configuration,

initialization strategy,

convergence diagnostics,

software versions,

Conda environment,

GitHub repository,

permanent Zenodo archive,

complete computational workflow.

These revisions substantially improve methodological transparency and reproducibility.

Major Comment 2

Biological interpretation of OU parameters should be tempered.

Response

We fully agree.

The statistical interpretation of the OU parameters is clarified in the Methods on pages 9–11, lines 200–268, and the biological limitations are discussed on pages 25–26, lines 614–642.

Throughout the manuscript we now consistently describe

μ as the effective equilibrium state,

θ as an effective mean-reversion parameter,

σ as effective stochastic variability.

We removed or substantially softened previous mechanistic language such as

“restoration of stabilizing constraints,”

“evolutionary instability,”

“phenotypic dispersion.”

Instead, we now explicitly state throughout the Methods, Results, and Discussion that these parameters are effective statistical summaries under the assumed OU model rather than direct measurements of biological mechanisms or causal evolutionary forces.

Major Comment 3

Parameter identifiability should be demonstrated more quantitatively.

Response

We substantially expanded both the empirical sampling characterization and the simulation benchmark. The distribution of observation counts, follow-up durations, consecutive sampling intervals, and patient-level sampling irregularity is now described on pages 7–8, lines 170–198, and summarized in Fig S1 and Table S1. The simulation design, estimation methods, performance metrics, and prespecified reliability criteria are described on pages 14–15, lines 335–368. Quantitative recovery and reliability results are reported on pages 20–21, lines 471–501, in Fig 2 and Tables S2–S3. The Discussion now explicitly distinguishes computational convergence from statistical identifiability on pages 28–29, lines 693–711.

Major Comment 4

Clinical-stage definitions should be clarified.

Response

We agree that the origin and use of the clinical-stage categories required clarification. These groups were not newly defined in the present study. Rather, we adopted the predefined clinical-group labels reported in the original KMT2A-rearranged leukemia cohort of Ahlgren et al. [1]. We have now clarified this in the Methods and state explicitly that the present analysis did not redefine or independently reconstruct the Early, Early/refractory, Late, Remission, Very early, and Very early/refractory categories. Each modeled patient retained the single group label provided in the source dataset for hierarchical Bayesian inference. The complete patient-to-group mapping is provided in S4 Data, and group-specific sample sizes are summarized in Table S4 (page 7, lines 160–168).

Major Comments 5–6

Poor predictive performance and alternative stochastic processes should be discussed more carefully.

Response

We agree that the distinction among parameter inference, uncertainty calibration, and point prediction required clearer treatment. We therefore added a cohort-wide posterior predictive analysis for all 47 modeled patients. Mean coverage of the nominal 95% posterior predictive interval was approximately 94%, and median patient-level coverage was 100%, although coverage varied across groups. Predictive R^2 was negative for many patients, indicating that the posterior-mean trajectory often reconstructed the observed series less accurately than the patient-specific observed mean. We now explicitly state that this finding limits point-trajectory reconstruction but does not imply failed MCMC convergence or invalidate the posterior distributions of the OU parameters (pages 24–25, lines 570–610, Fig 5, and Tables S10–S12).

We also added a dedicated Discussion subsection that distinguishes posterior parameter inference, posterior predictive coverage, and point-trajectory reconstruction on pages 27–28, lines 666–691. Alternative stochastic formulations—including Brownian motion, switching OU models, hidden Markov models, nonlinear stochastic differential equations, jump or Lévy processes, and explicit branching models—are discussed on pages 29-30, lines 715–730.

Minor Comment 1

Present the OU stochastic differential equation explicitly.

Response

We have added the complete OU stochastic differential equation and exact conditional transition distribution to the Methods (pages 9–11, lines 200–268).

The complete stochastic differential equation is now presented in the Methods together with its biological interpretation and exact transition density.

Minor Comment 2

Improve figure resolution.

Response

All figures were regenerated and will be submitted as separate publication-quality TIFF files at the resolution required by PLOS ONE. Text, line weights, and panel labels were also enlarged to improve readability.

Minor Comment 3

Improve terminology consistency.

Response

The terminology has been standardized throughout the manuscript.

The revised manuscript consistently uses:

effective mean reversion,

stochastic variability,

drift-to-noise ratio,

effective dynamical characteristics.

Mechanistic terminology has been substantially reduced.

Minor Comment 4

Better distinguish prediction, reconstruction, and mechanistic interpretation.

Response

A dedicated Discussion subsection now explicitly distinguishes these concepts and explains their different interpretations.

Minor Comment 5

Quantify posterior interval coverage.

Response

The Results now report mean coverage of approximately 94% for the nominal 95% posterior predictive interval and median patient-level coverage of 100% (page 24, lines 572–579). Patient-level, group-level, and correlation-based diagnostics are provided in Fig 5 and Tables S10–S12.

Ethics statement

The ethics statement should be consistent.

Response

We corrected the online submission metadata so that it is fully consistent with the manuscript. The study used previously published, publicly available data and involved no new participant recruitment or collection of identifiable private information. The revised ethics statement now accurately reflects the status of the secondary data analysis.

Reviewer #2

We sincerely thank the reviewer for the encouraging evaluation and constructive suggestions.

Prior distributions

Please clarify prior distributions.

Response

The Methods now explicitly specify the prior distributions, hyperparameters, parameter constraints, hierarchical parameterization, and residual transition-scale prior on pages 11–13, lines 270–305.

MCMC convergence

Please report convergence metrics.

Response

We now report four chains, 2,500 tuning iterations and 2,000 retained draws per chain, the jitter+adapt_diag initialization strategy, target acceptance probability, R̂, bulk and tail effective sample sizes, divergent transitions, and tree-depth events on pages 13–14, lines 307–333. Complete convergence summaries are also provided as S13 Data, with run metadata in S14 Data.

The Results report a maximum R̂ of 1.003, minimum bulk and tail ESS values exceeding 4,100, no divergent transitions, and no maximum tree-depth events (page 21, lines 503–510).

Data scaling

Clarify within-patient scaling.

Response

We clarified that no within-patient centering, z-score transformation, unit-variance standardization, or other within-patient rescaling was applied. The longitudinal x and n variables were constructed deterministically from the clinical annotations according to the stated rules and entered the analysis without additional rescaling (pages 6–7, lines 127–158).

Additional Journal Requirements

Formatting

The manuscript has been revised to conform to the current PLOS ONE formatting requirements.

Code availability

The complete computational pipeline is now openly available through GitHub.

A permanent archived version has also been deposited on Zenodo.

Data availability

The online Data Availability Statement has been revised to provide the DOI and available direct source information for the original KMT2A-rearranged leukemia cohort. The permanent Zenodo archive contains the processed model inputs, simulation outputs, posterior summaries, convergence diagnostics, and reproducibility files generated for the present study, while the unrestricted analysis code is available through GitHub.

Related manuscript

The previously mentioned related manuscript is:

“Kim SH. Hierarchical Bayesian OU–Branching Models Capture Stochastic and Deterministic Forces in Bacterial Mutation Evolution.”

This manuscript is currently under consideration at Bioinformatics Advances and has not been published.

The present manuscript remains entirely distinct.

Specifically:

it analyzes an independent pediatric leukemia dataset,

it addresses a different biological question,

it contains different figures,

it contains different simulations,

it develops a substantially expanded hierarchical Bayesian OU framework focused on stage-associated tumor evolution rather than bacterial mutation dynamics.

Accordingly, there is no overlap in datasets, analyses, figures, or conclusions, and the present manuscript does not constitute duplicate publication.

We again sincerely thank the Academic Editor and both reviewers for their careful reading and highly constructive suggestions. Their comments have substantially strengthened the manuscript. We hope that the revised version satisfactorily addresses all concerns and will now be suitable for publication in PLOS ONE.

Sincerely,

Seung-Hwan Kim

Attachment Submitted filename: Response_to_Reviewers.pdf

# Peer review: Decision Letter 1

**DOI:** 10.1371/journal.pone.0357755.r003

**Authors:** Hodaka Fujii

6 Aug 2026

Dear Dr. Kim,

Thank you for submitting your manuscript to PLOS ONE. After careful consideration, we feel that it has merit but does not fully meet PLOS ONE’s publication criteria as it currently stands. Therefore, we invite you to submit a revised version of the manuscript that addresses the points raised during the review process.

Please submit your revised manuscript by Sep 20 2026 11:59PM. If you will need more time than this to complete your revisions, please reply to this message or contact the journal office at plosone@plos.org. When you're ready to submit your revision, log on to https://www.editorialmanager.com/pone/ and select the 'Submissions Needing Revision' folder to locate your manuscript file.

- A letter that responds to each point raised by the academic editor and reviewer(s). You should upload this letter as a separate file labeled 'Response to Reviewers'.
- A marked-up copy of your manuscript that highlights changes made to the original version. You should upload this as a separate file labeled 'Revised Manuscript with Track Changes'.
- An unmarked version of your revised paper without tracked changes. You should upload this as a separate file labeled 'Manuscript'.

If you would like to make changes to your financial disclosure, please include your updated statement in your cover letter. Guidelines for resubmitting your figure files are available below the reviewer comments at the end of this letter.

If applicable, we recommend that you deposit your laboratory protocols in protocols.io to enhance the reproducibility of your results. Protocols.io assigns your protocol its own identifier (DOI) so that it can be cited independently in the future. For instructions see: https://journals.plos.org/plosone/s/submission-guidelines#loc-laboratory-protocols. Additionally, PLOS ONE offers an option for publishing peer-reviewed Lab Protocol articles, which describe protocols hosted on protocols.io. Read more information on sharing protocols at https://plos.org/protocols?utm_medium=editorial-email&utm_source=authorletters&utm_campaign=protocols.

As the corresponding author, your ORCID iD is verified in the submission system and will appear in the published article. PLOS supports the use of ORCID, and we encourage all coauthors to register for an ORCID iD and use it as well. Please encourage your coauthors to verify their ORCID iD within the submission system before final acceptance, as unverified ORCID iDs will not appear in the published article. *Only*  the individual author can complete the verification step; PLOS staff *cannot*  verify ORCID iDs on behalf of authors.

We look forward to receiving your revised manuscript.

Kind regards,

Hodaka Fujii, M.D., Ph.D.

Academic Editor

PLOS One

Journal Requirements:

If the reviewer comments include a recommendation to cite specific previously published works, please review and evaluate these publications to determine whether they are relevant and should be cited. There is no requirement to cite these works unless the editor has indicated otherwise.

Please review your reference list to ensure that it is complete and correct. If you have cited papers that have been retracted, please include the rationale for doing so in the manuscript text, or remove these references and replace them with relevant current references. Any changes to the reference list should be mentioned in the rebuttal letter that accompanies your revised manuscript. If you need to cite a retracted article, indicate the article’s retracted status in the References list and also include a citation and full reference for the retraction notice.

[Note: HTML markup is below. Please do not edit.]

Reviewers' comments:

Reviewer's Responses to Questions

**Comments to the Author**

Reviewer #1: (No Response)

Reviewer #2: All comments have been addressed

**********

2. Is the manuscript technically sound, and do the data support the conclusions?

Reviewer #1: Yes

Reviewer #2: Yes

**********

3. Has the statistical analysis been performed appropriately and rigorously?

Reviewer #1: No

Reviewer #2: Yes

**********

4. Have the authors made all data underlying the findings in their manuscript fully available?

The PLOS Data policy

Reviewer #1: Yes

Reviewer #2: Yes

**********

5. Is the manuscript presented in an intelligible fashion and written in standard English?

Reviewer #1: Yes

Reviewer #2: Yes

**********

Reviewer #1: The manuscript is generally well written following revision. Here're several additional comments:

1. Add a summary table describing the notation of the major model parameters to improve readability.

2. The Discussion appropriately acknowledges alternative stochastic processes but the manuscript does not clearly explain why the OU process is particularly appropriate for the present biological problem beyond its mean-reverting property. A more balanced comparison of the modeling assumptions, advantages, and limitations of the OU process relative to alternative approaches would strengthen the methodological justification.

3. The study is based on a single pediatric KMT2A-rearranged leukemia cohort consisting of 52 patients. Although the authors appropriately acknowledge the limitations of sparse sampling, the Discussion could more explicitly address whether the proposed framework is expected to generalize to other leukemia subtypes, adult malignancies, or longitudinal cancer datasets with different sampling structures. This would better position the broader applicability of the proposed methodology.

4. Throughout the manuscript, the authors emphasize that the study is intended primarily as a statistical modeling framework rather than a predictive model. Nevertheless, the Discussion occasionally shifts between describing methodological advances and biological interpretation. A clearer separation between (i) methodological contributions, (ii) biological observations supported by the current data, and (iii) future applications would improve the overall narrative and help readers understand the principal contribution of the study

Reviewer #2: (No Response)

**********

what does this mean?). If published, this will include your full peer review and any attached files.

If you choose “no”, your identity will remain anonymous but your review may still be made public.

**Do you want your identity to be public for this peer review?**  For information about this choice, including consent withdrawal, please see our Privacy Policy

Reviewer #1: **Yes:** Yuhang Liu

Reviewer #2: No

**********

[NOTE: If reviewer comments were submitted as an attachment file, they will be attached to this email and accessible via the submission site. Please log into your account, locate the manuscript record, and check for the action link "View Attachments". If this link does not appear, there are no attachment files.]

To ensure your figures meet our technical requirements, please review our figure guidelines: https://journals.plos.org/plosone/s/figures

You may also use PLOS’s free figure tool, NAAS, to help you prepare publication quality figures: https://journals.plos.org/plosone/s/figures#loc-tools-for-figure-preparation.

NAAS will assess whether your figures meet our technical requirements by comparing each figure against our figure specifications.

# Author comment: Author response to Decision Letter 2

**DOI:** 10.1371/journal.pone.0357755.r004

6 Aug 2026

Response to Reviewers

Manuscript ID: PONE-D-26-18451R2

Title: Stage-specific evolutionary dynamics in pediatric leukemia inferred using Bayesian stochastic modeling

Dear Dr. Fujii,

We sincerely thank you and the reviewers for your careful evaluation of our revised manuscript. We greatly appreciate the reviewers’ positive assessment that our previous revisions had addressed the major concerns, as well as the constructive suggestions that further improved the manuscript.

In response to the remaining comments, we have revised the manuscript to improve clarity, readability, and the presentation of the methodological framework. No additional analyses were required, and the scientific conclusions remain unchanged. All changes have been incorporated into both the marked-up and clean versions of the manuscript.

Our detailed responses are provided below.

Academic Editor

We sincerely thank the Academic Editor for the careful evaluation of our revised manuscript and for the opportunity to submit a further minor revision. We appreciate the Editor’s assessment that the manuscript has merit and that the remaining issues primarily concern clarification and presentation. We have carefully addressed all comments raised by Reviewer #1.

Reviewer #1

We sincerely thank the reviewer for the thoughtful evaluation of our revised manuscript and for recognizing that the major comments from the previous review had been satisfactorily addressed. We appreciate these constructive suggestions, which have further improved the clarity and accessibility of the manuscript.

Comment 1

“Add a summary table describing the notation of the major model parameters to improve readability.”

Response

We appreciate this helpful suggestion.

To improve readability, we have added Table 1 (“Summary of notation used throughout the hierarchical OU model”) immediately following the Study Overview section in the Methods (page 5, lines 110-114). The table summarizes the principal mathematical symbols used throughout the manuscript, including the modeled longitudinal state, observation variables, equilibrium state, mean-reversion rate, diffusion scale, residual transition parameter, observation interval, and Wiener process.

In addition, we now explicitly refer readers to this table at the end of the Study Overview.

Comment 2

“The Discussion appropriately acknowledges alternative stochastic processes, but the manuscript does not clearly explain why the OU process is particularly appropriate for the present biological problem beyond its mean-reverting property. A more balanced comparison of the modeling assumptions, advantages, and limitations of the OU process relative to alternative approaches would strengthen the methodological justification.”

Response

Thank you for this valuable suggestion.

We have expanded the Discussion to provide a more explicit justification for selecting the Ornstein–Uhlenbeck process for sparse longitudinal leukemia data (page 27, lines 638-645). The revised text now explains that leukemia progression is expected to evolve under biological constraints imposed by developmental programs, therapeutic intervention, immune surveillance, and related regulatory processes rather than unrestricted random diffusion. We further contrast the OU process with Brownian motion and discuss why its parsimonious formulation is advantageous under sparse and irregular sampling.

We have also expanded the discussion of alternative stochastic modeling approaches, including Brownian motion, hidden Markov models, nonlinear stochastic differential equations, Lévy jump processes, and branching processes (page 31, lines 745-752). The revised manuscript emphasizes that these approaches should be viewed as complementary rather than competing methodologies and that model selection should ultimately be guided by the biological question, data availability, and statistical identifiability rather than by any single universally optimal stochastic formulation.

Comment 3

“The study is based on a single pediatric KMT2A-rearranged leukemia cohort consisting of 52 patients. Although the authors appropriately acknowledge the limitations of sparse sampling, the Discussion could more explicitly address whether the proposed framework is expected to generalize to other leukemia subtypes, adult malignancies, or longitudinal cancer datasets with different sampling structures.”

Response

We appreciate this important suggestion.

To address this point, we have added a new subsection entitled “Generalizability” (pages 31-32, lines 754-765). In this section, we clarify that although the present application focuses on pediatric KMT2A-rearranged leukemia, the Bayesian Ornstein–Uhlenbeck framework itself is not disease specific. We discuss its potential applicability to other pediatric leukemias, adult hematologic malignancies, solid tumors, and additional longitudinal biological datasets that exhibit constrained stochastic dynamics.

At the same time, we explicitly note that successful application to other disease settings will require independent validation and careful evaluation of the underlying modeling assumptions, sampling density, and observation process.

Comment 4

“Throughout the manuscript, the authors emphasize that the study is intended primarily as a statistical modeling framework rather than a predictive model. Nevertheless, the Discussion occasionally shifts between describing methodological advances and biological interpretation. A clearer separation between (i) methodological contributions, (ii) biological observations supported by the current data, and (iii) future applications would improve the overall narrative and help readers understand the principal contribution of the study.”

Response

We thank the reviewer for this excellent recommendation.

To improve the overall organization of the Discussion, we have substantially reorganized the Discussion to provide a clearer distinction between methodological contributions, biological observations, future applications, limitations, and the concluding interpretation (pages 32-35, lines 767-863). The revised manuscript now clearly separates:

• Methodological implications, emphasizing the statistical contributions of the Bayesian hierarchical OU framework and uncertainty-aware inference;

• Biological observations, summarizing the descriptive findings supported by the present dataset while carefully avoiding mechanistic overinterpretation;

• Future applications, discussing potential extensions to additional disease settings and more flexible stochastic modeling frameworks;

• Limitations, presenting the remaining methodological constraints of the current study; and

• Conclusion, emphasizing that the principal contribution of this work is an interpretable and uncertainty-aware statistical framework rather than a predictive or mechanistic model.

We believe that this revised organization substantially improves the clarity of the manuscript and more clearly distinguishes methodological contributions from biological interpretation, as suggested.

Reviewer #2

We sincerely thank the reviewer for the positive evaluation of our revised manuscript and for indicating that all previous comments had been satisfactorily addressed. We greatly appreciate the reviewer’s careful assessment and encouraging recommendation.

Once again, we sincerely thank the Academic Editor and both reviewers for their thoughtful evaluation of our work. We believe that the revisions have substantially improved the clarity and accessibility of the manuscript, and we hope that the revised version is now suitable for publication in PLOS ONE.

Sincerely,

Seung-Hwan Kim, Ph.D.

Department of Biology, Fisher College

Department of Pediatric Oncology, Dana-Farber Cancer Institute

Attachment Submitted filename: Response_to_Reviewers_auresp_2.pdf

# Editor report: Decision Letter 2

**DOI:** 10.1371/journal.pone.0357755.r005

**Authors:** Hodaka Fujii

23 Aug 2026

Stage-specific evolutionary dynamics in pediatric leukemia inferred using Bayesian stochastic modeling

PONE-D-26-18451R2

Dear Dr. Kim,

We’re pleased to inform you that your manuscript has been judged scientifically suitable for publication and will be formally accepted for publication once it meets all outstanding technical requirements.

Within one week, you’ll receive an e-mail detailing the required amendments. When these have been addressed, you’ll receive a formal acceptance letter and your manuscript will be scheduled for publication.

An invoice will be generated when your article is formally accepted. Please note, if your institution has a publishing partnership with PLOS and your article meets the relevant criteria, all or part of your publication costs will be covered. Please make sure your user information is up-to-date by logging into Editorial Manager at Editorial Manager® and clicking the ‘Update My Information' link at the top of the page. For questions related to billing, please contact billing support.

If your institution or institutions have a press office, please notify them about your upcoming paper to help maximize its impact. If they’ll be preparing press materials, please inform our press team as soon as possible -- no later than 48 hours after receiving the formal acceptance. Your manuscript will remain under strict press embargo until 2 pm Eastern Time on the date of publication. For more information, please contact onepress@plos.org.

Kind regards,

Hodaka Fujii, M.D., Ph.D.

Academic Editor

PLOS One

Additional Editor Comments (optional):

Reviewers' comments:

# Editor report: Acceptance letter

**DOI:** 10.1371/journal.pone.0357755.r006

**Authors:** Hodaka Fujii

PONE-D-26-18451R2

PLOS One

Dear Dr. Kim,

I'm pleased to inform you that your manuscript has been deemed suitable for publication in PLOS One. Congratulations! Your manuscript is now being handed over to our production team.

At this stage, our production department will prepare your paper for publication. This includes ensuring the following:

* All references, tables, and figures are properly cited

* All relevant supporting information is included in the manuscript submission,

* There are no issues that prevent the paper from being properly typeset

You will receive further instructions from the production team, including instructions on how to review your proof when it is ready. Please keep in mind that we are working through a large volume of accepted articles, so please give us a few days to review your paper and let you know the next and final steps.

Lastly, if your institution or institutions have a press office, please let them know about your upcoming paper now to help maximize its impact. If they'll be preparing press materials, please inform our press team within the next 48 hours. Your manuscript will remain under strict press embargo until 2 pm Eastern Time on the date of publication. For more information, please contact onepress@plos.org.

You will receive an invoice from PLOS for your publication fee after your manuscript has reached the completed accept phase. If you receive an email requesting payment before acceptance or for any other service, this may be a phishing scheme. Learn how to identify phishing emails and protect your accounts at https://explore.plos.org/phishing.

If we can help with anything else, please email us at customercare@plos.org.

Thank you for submitting your work to PLOS One and supporting open access.

Kind regards,

PLOS One Editorial Office Staff

on behalf of

Dr. Hodaka Fujii

Academic Editor

PLOS One
